# Supplementary material for: Building a Tool Kit for Medical and Dental Students: Addressing Microaggressions and Discrimination on the Wards
Source: MedEdPORTAL. 2020 Apr 3;16:10893. doi: 10.15766/mep_2374-8265.10893 (PMC7187912; doi:10.15766/mep_2374-8265.10893)
Supplement: Supplementary file 1 — PowerPoint Presentation.pptxCases.docxRole Cards.docxFramework Handout.docxFacilitator Guide.docxAbridged Facilitator Guide.docxPreworkshop Survey.docxPostworkshop Survey.docxText Exercise Criteria.docx [file mep-16-10893-s001.zip › _Educational_Summary_Report_10893.pdf]

MedEdPORTAL® | The AAMC Journal of Teaching and Learning Resources

Original Publication

Open Access

Building a Tool Kit for Medical and Dental Students: Addressing Microaggressions and Discrimination on the Wards

Raquel Sofia Sandoval*, Titilayo Afolabi, Jordan Said, Spencer Dunleavy, MSc, Avik Chatterjee, MD, MPH, Daniele Ölveczky, MD, MS

*Corresponding Author: raquelsofia_sandoval@hms.harvard.edu

Abstract

Introduction: Microaggressions, subtle slights related to characteristics such as race, gender, or sexual orientation, in a clinical setting can sabotage the therapeutic alliance. Curricula tailored specifically towards medical students that raise awareness of microaggressions and aim to change behavior are absent. **Methods:** We created a 2-hour workshop to prepare preclinical medical and dental students to recognize and respond to microaggressions in clinical practice. The workshop consisted of a didactic portion describing microaggressions and strategies for responding to them and a case-based small-group portion to practice strategies. Participants completed electronic pre- and postworkshop surveys. **Results:** Of 163 students participating in the workshop, 121 (74%) completed the preworkshop survey, 105 (64%) completed the postworkshop survey, and 81 (50%) completed both. Preworkshop, 48% reported female gender, and 36% reported underrepresented in medicine status. The majority (77%) had witnessed or experienced microaggressions in the clinical setting, and 69% reported very good or excellent familiarity with the concept of microaggressions. The curriculum appeared to significantly mitigate challenges associated with microaggressions, including reductions in perceived difficulty in identifying microaggressions ($p < .001$), being unsure what to do or say ($p < .001$), improvements in familiarity with institutional support systems ($p < .001$), and awareness of the clinical relevance of microaggressions ($p < .001$). **Discussion:** Given the high self-reported prevalence of microaggressions in the clinical setting, students need the skills to respond. This innovative session improves readiness to address microaggressions by helping participants build and practice these skills in a supportive environment.

Keywords
Racism, Microaggression, Discrimination, Tool Kit, Dentist, Physician, Communication Skills, Cultural Competence, Curriculum Development, Diversity, Inclusion, Health Equity, Leadership Development/Skills, Professionalism, Case-Based Learning

Educational Objectives

By the end of this session, learners will be able to:

1. Recognize instances of microaggressions and discrimination in the clinical setting.
2. Describe the impact of microaggressions and discrimination on clinical care.
3. Explain challenges to responding to microaggressions in the clinical setting.
4. Apply the presented frameworks to clinical cases in observed role-plays.

Citation:
Sandoval RS, Afolabi T, Said J, Dunleavy S, Chatterjee A, Ölveczky D. Building a tool kit for medical and dental students: addressing microaggressions and discrimination on the wards. *MedEdPORTAL*. 2020;16:10893. https://doi.org/10.15766/mep_2374-8265.10893

Introduction

Health inequities based on race, gender identity, sexual orientation, socioeconomic status, nationality, and other factors have persisted over decades, though exactly how to eliminate these inequities at their source is unknown. While structural issues—such as housing discrimination and criminal justice—indisputably contribute to these inequities, wide health disparities persist (e.g., hospital segregation) despite the dismantling of parts of these systems.[1] Attention has turned toward mounting evidence suggesting that at the individual level, implicit bias is widespread in medicine and may negatively affect patient care.[2-7] As a result, one possible strategy for eliminating health inequities has been to better characterize and mitigate implicit bias among health care providers.

Historically, individual provider bias has been managed by providing training around cultural competency.[8] Cultural competency mainly focuses on knowledge acquisition about the differing values and belief systems underrepresented

Copyright © 2020 Sandoval et al. This is an open-access publication distributed under the terms of the Creative Commons Attribution license.

groups hold.[8] Yet, without recognition of the role of systemic racism and providers' own biases, this approach can lead to oversimplification of culture and perpetuation of stereotypes. In response, experts have proposed that the concept of cultural humility replace cultural competency in order to shift the narrative to include providers' self-awareness of their own unconscious and conscious biases.[9,10] However, both models have been critiqued for their failure to address the structural inequalities that underlie disparities.[11] Additionally, both models fail to fully demonstrate the way biases manifest explicitly in patient care.

Recently, there has been growing support for investigating microaggressions as a target for mitigating individual provider bias. Sue and colleagues implicate microaggressions as a manifestation of bias from health care providers towards patients.[12] They further explain how microaggressions create barriers to clinical practice by sabotaging the creation of a therapeutic alliance and thus recommend trainee education about microaggressions.[12] Microaggressions can be defined as casual debasements of any group.[13,14] They occur as commonplace verbal, behavioral, and environmental indignities, whether intentional or unintentional, that communicate hostile, derogatory, or negative slights and insults.[13] They can be intersectional and simultaneously encompass more than one axis, such as income, social capital, religion, ableness, race, gender, sexual orientation, and more.[13,14] Discrimination, in contrast to microaggressions, is the explicit, often systems-level, unfair or prejudicial treatment of people and groups based on characteristics such as race, gender, age, or sexual orientation.

Both patients and providers can be on the receiving end of microaggressions. Repeatedly experiencing microaggressions and discrimination can lead to both mental and physical health effects, including post-traumatic stress.[14-16] For physicians, these types of effects can in turn contribute to emotional exhaustion, a characteristic of burnout.[17] Additionally, those more likely to experience microaggressions and discrimination are physicians with marginalized identities, particularly those with multiple marginalized identities.[18,19] For patients, physicians' biases can influence diagnosis and treatment options, perpetuating health care disparities.[2,20-22] Development of curricula to help physicians and physicians in training address and ultimately prevent these instances is critical to the sustainability of the physician workforce and, ultimately, high-quality patient care.[23-25]

Different institutions and programs have attempted to address microaggressions in the clinical setting. Models by Whitgob, Blankenburg, and Bogetz[26] and Paul-Emile, Smith, Lo, and Fernández[27] provide guidance for how to approach situations where the patient and/or family is being discriminatory. These frameworks emphasize assessment of illness acuity, depersonalization of the situation, and building alliance through negotiation. Another framework, Mostow and colleagues' RESPECT, is a communication tool that puts emphasis on the building of trust across differences.[28] Most recently, Wheeler, Zapata, Davis, and Chou have provided 12 tips for responding to microaggressions and discrimination either seen or experienced on the wards.[29] However, all of these frameworks and tips have been created for faculty use, not necessarily for medical student use. Within *MedEdPORTAL,* there also exist publications with curricula for workshops to address bias and/or build resilience in the clinical setting.[11,24,30] However, these too mainly have a target audience of residents and team leaders, rarely focusing on medical students. Furthermore, curricula intended for medical students often prioritize building skills for patient interviewing rather than cultivating skills for navigating interpersonal problems in the workplace or classroom.[31,32]

One framework designed for medical students is the Georgetown University School of Medicine's Stop, Talk, Roll.[33] The model provides an algorithm for addressing tough communication exchanges within the clinical setting. It also offers suggested phrases to carry out each step, as well as information about Georgetown-specific policies and support offices. Furthermore, it breaks down interactions into those happening between student and patient as well as between student and other health care team members.

Recognizing the gap in skills-based education on microaggressions for medical students, we developed a workshop to (1) introduce the concepts and effects of microaggressions and discrimination to first-year medical and dental students enrolled in a professional development longitudinal course and (2) provide a framework (based on Stop, Talk, Roll) for how to address these instances when they occur in the clinical setting regardless of whether the student directly experiences or witnesses them. This innovative workshop employs educational methods appropriate for adult learners in order to build the necessary skills to begin to address microaggressions and discrimination in the clinical setting. It also provides a complementary and practical curricular technique to help students practice encountering these scenarios and develop their own tool kit of phrases and resources to navigate them.

Methods

This workshop was developed by a team of students and faculty and based on a case-study approach, which has been shown to

Copyright © 2020 Sandoval et al. This is an open-access publication distributed under the terms of the Creative Commons Attribution license.

be a highly effective way to teach medical students, particularly for higher-level skills such as analysis.[34-36] We integrated the workshop into the third of a set of 3 longitudinal professional development weeks during the preclinical curriculum, before students began their clinical clerkships. The session provided the setting for students to reflect on and practice how they would address certain clinical and professional situations. The professional development weeks included workshops and lectures on different subjects relating to being a physician, such as working on interprofessional teams, time management, and building resilience through self-care and reflection. Weekly clinical sessions over the course of the preclinical training allowed students to have a context, such as the hierarchical nature of the medical team, for the cases used during the workshop.

We created a workshop with a two-part presentation format: (1) a short and interactive large-group didactic session via PowerPoint (PPT) presentation that introduced students to key terms and frameworks for addressing these issues in the clinical setting and (2) a small-group session during which students worked through two cases by applying the presented frameworks and role-playing scenarios.

- Time line of workshop (total time: 2 hours).
  - 30 minutes: large-group didactic.
  - 15 minutes: transition time.
  - 1 hour and 15 minutes: small group.
    - 10 minutes: introduction to session, group norms.
    - 30 minutes: case 1.
    - 30 minutes: case 2.
    - 5 minutes: closing, postsurvey completion.

The PPT (Appendix A) presented two main frameworks: Sue and colleagues' categories of and relationships among racial microaggressions[12] and the Georgetown University School of Medicine's Stop, Talk, Roll.[33] The first model was used to identify types of microaggressions, and the second was used as a framework for how to act and what to say when these encounters occur, both between a provider and patient and between providers. The PPT also included a short case used to model the application of the frameworks. Additionally, the PPT contained interactive components through the use of Poll Everywhere to gage audience response to the case. Finally, data that had been collected through the preworkshop survey were summarized on a few slides that drove home the message that microaggressions had been widely witnessed or experienced by audience members. The PPT (Appendix A) contains detailed notes about its presentation, including where to include Poll Everywhere and preworkshop survey data.

The small groups comprised eight to 12 students and had two cofacilitators: a faculty member from a clinical site where medical and dental students rotate and either a resident, staff member, or fourth-year medical student. The small size of the groups was intended to create a safer and more intimate space, as well as to allow for proper small-group facilitation. We recruited facilitators with prior experience or interest in leading discussions around issues of justice, diversity, and inclusion via existing networks within the medical school and affiliated hospitals. The small groups went over cases (Appendix B) and used role cards with instructions to guide the discussion of the cases (Appendix C). Students were also given a copy of the frameworks presented in the PPT to reference in the session and take home (Appendix D). To prepare for the session, the cofacilitators had a detailed facilitator guide (Appendix E), which was emailed out 1 week prior to the workshop. The facilitator guide provided information regarding how to run the small-group portion, including how to work through the cases, what particular points to highlight, and additional discussion questions for the small group to consider. The abridged facilitator guide (Appendix F) had key logistic information and a time line for the facilitators to reference during the session itself. In the small-group portion of the workshop, each group had its own room apart from the other groups. There was no technology needed to carry out the small-group session; all the materials used (Appendices B-D) were printed and distributed to the cofacilitators of the small groups before the session. The materials were assembled into small packets that contained one copy of the cases, one role card for case 1 and another for case 2, and one copy of the frameworks handout. The small packets were distributed by the cofacilitators to the students in their small groups.

The cases used were adapted from lived and witnessed experiences of the students and faculty organizing the curriculum. Case 1 discussed an elderly patient making inappropriate and hostile comments toward female-presenting trainees and a supervising attending commenting on the team's professionalism in not addressing the situation. It explored topics including racism, sexual harassment, and the model minority myth.[37,38] Case 2 depicted a medical student being asked to take on extra roles and tasks because of shared identities with the patient. It explored topics including the minority tax, racism, and internalized bias.[39]

Copyright © 2020 Sandoval et al. This is an open-access publication distributed under the terms of the Creative Commons Attribution license.

This workshop, though originally designed for medical and dental students, can also be implemented with medical residents. While participants are not required to have clinical experience, they should have had some exposure to the clinical setting so that the cases seem more relevant and real. Exposure to the clinical setting preferably would include dedicated clinical experiences that are already part of the individual medical school's curriculum rather than solely relying on students' prior personal clinical encounters. The lead facilitators should be faculty members with MD or DO degrees and ideally have interest in equity and justice work/scholarship. If two coleaders arise, an effort should be made for them to divide up the work and meet periodically to ensure the work is divided equally and in an integrative manner. Other small-group facilitators could include faculty, residents, staff, and senior medical and dental students. The original timing of the workshop was 2 hours: 30 minutes for the large-group didactic portion, 15 minutes for transition time/buffer zone, and 75 minutes for the small-group portion. However, 75 minutes may not be enough time to fully discuss both cases. Thus, it would be optimal to allot more time to the small-group portion or divide the small-group portion into two sessions, one for each case, to maximize the opportunity to take a deep dive into discussions and nuances of each case. If this is done, we recommend scheduling one small-group session directly after the large group and then staggering another small-group session in the following weeks.

We invited all participants to complete pre- and postworkshop surveys (Appendices G and H, respectively). Review of the literature revealed that a validated instrument to assess participants' responses to microaggression training did not exist. Previous existing microaggression scales (e.g., the Racial and Ethnic Microaggressions Scale[40] and the Racial Microaggressions Scale[41]) were thought to be unwieldy and did not address the microaggressions that trainees commonly face. To create the surveys, we conducted a literature review and interviewed students. This information was then synthesized to develop themes. Expert validation was performed by faculty at the Shapiro Institute for Education. Prior to administering the surveys, we reviewed them using cognitive interviewing to ensure that respondents would interpret items in the manner that they should. We designed the surveys to determine (1) respondents' level of familiarity with the concept of microaggressions, (2) their understanding of the definition of a microaggression, (3) their ability to identify microaggressions in a case study, (4) their self-rating of perceived barriers to addressing microaggressions in the clinical setting, and (5) demographics such as respondents' self-identified gender and underrepresented in medicine (URM) status. The Association of American Medical Colleges defines URM as "those racial and ethnic populations that are underrepresented in the medical profession relative to their numbers in the general population."[42] However, we chose to use a broader definition that included other aspects of identity, such as socioeconomic status and sexual orientation, and also gave space for other underrepresented groups, such as first generation in medicine. We wanted to be as inclusive as possible in how we defined URM in order to better capture how widely microaggressions occur and that they happen to people with different URM identities not exclusive to race and ethnicity. The preworkshop survey was sent out via email a few days before the workshop, with periodic email reminders to students to submit a response. The data collected from this survey were summarized into a few slides on the PPT to share during the large-group portion of the workshop, as mentioned above. The postworkshop survey was sent during the small-group portion of the workshop, and students were encouraged to spend the last minutes of the small-group portion filling it out. Participants were also asked to rate on a Likert scale how significant they felt certain barriers were to addressing microaggressions happening on the wards. Postworkshop surveys repeated the items asking participants to define microaggressions, as well as the self-rating of perceived barriers. We linked pre- and postworkshop survey responses with a unique identifier that participants created themselves to preserve anonymity and allow for paired analysis.

We also reported participants' self-reported exposure to and familiarity with microaggressions and their self-rated perception of barriers to addressing microaggressions. Two of the authors independently assessed accuracy of definition of the term microaggression and performance on the text exercise based on prespecified criteria (Appendix I). Scorers were not blinded to pre- or postworkshop status. We evaluated changes in paired pre- and postworkshop definitions and perceived barriers using paired-signed rank testing. We compared responses between subgroups by gender, self-identified URM status, and whether participants had previously experienced or witnessed microaggressions (EMA) or not (never experienced or witnessed microaggressions [NEMA]). Comparisons for distributions of responses across subgroups were tested using Kolmogorov-Smirnov two-sample tests, and overall distribution test statistics are reported.

This study was determined to be exempt from review by the Harvard Medical School Institutional Review Board.

Copyright © 2020 Sandoval et al. This is an open-access publication distributed under the terms of the Creative Commons Attribution license.

Results

Respondent Characteristics

Of 163 first-year medical and dental students who took part in the workshop, 121 respondents (74%) completed the preworkshop survey, 105 (64%) completed the postworkshop survey, and 81 completed both. On the preworkshop survey, 58 out of 121 respondents (48%) reported female gender, and 44 out of 116 (36%) reported URM status (Table 1). The sample of students who completed both preworkshop and postworkshop surveys did not appear to significantly differ from the overall class of first-year medical students in 2018 with respect to gender ($p = .42$) or URM status ($p = .09$).

Encountering Microaggressions

Preworkshop, 95 out of 121 respondents (77%) had encountered some form of a microaggression during a clinical experience at least once. Sixty-five respondents (53%) reported experiencing a microaggression themselves, 80 (66%) reported witnessing a colleague experience a microaggression, and 62 (51%) reported witnessing a patient experiencing one. Self-identified URM students were not more likely to report a microaggression. Female respondents were significantly more likely to report experiencing microaggressions (odds ratio: 3.51, 95% confidence interval [CI], 1.60-7.69) or witnessing microaggressions towards a patient (odds ratio: 2.99, 95% CI, 1.38-6.45; Table 2). Accordingly, men were therefore more likely to have never experienced or witnessed microaggressions. Prior experiences with microaggressions before and during the first year of medical school were not separately examined.

Knowledge of and Familiarity With Microaggressions

In terms of preworkshop familiarity with microaggressions (Table 3), over half of the participants reported very good or excellent familiarity with microaggressions (69%). Across URM, gender, and EMA groups, over half from each group also reported very good or excellent familiarity with microaggressions (63%, 75%, and 69%, respectively). Preworkshop experiences of microaggressions were significantly associated with greater confidence in the ability to identify ($p < .001$) and address ($p = .005$) microaggressions.

Table 1. Characteristics of Medical and Dental Student Respondents ($N = 121$) to the Microaggressions Workshop Presurvey

Descriptor	No. (%)
Gender	
Male	54 (45%)
Female	58 (48%)
Other/declined to respond	9 (7%)
URM status	
URM	44 (36%)
Non-URM	72 (59%)
Other/declined to respond	5 (4%)

Abbreviation: URM, underrepresented in medicine.

Preworkshop, 59 respondents (48%) provided the correct definition for microaggressions, and postworkshop, 53 respondents (52%) provided the correct definition. Though a higher percentage of students responded correctly after the workshop, the distribution of definition scores did not appear to significantly change after the workshop ($Q = 2.61$, $p = .11$). Using Wilcoxon signed rank tests to assess individual changes in accuracy among 81 paired responses, the workshop did not appear to improve individual accuracy ($Z = 1.68$, $p = .09$). Changes in accuracy did not differ across URM, gender, and EMA groups.

Barriers to Responding to Microaggressions

Prior to the workshop, the barriers that respondents cited as the most challenging for addressing microaggressions were “Not sure what to do or say” (52% rating this as very challenging or extremely challenging) and “Fear of retribution” (43% rating this as very challenging or extremely challenging). For addressing episodes of frank discrimination, “Not sure what to do or say” and “Fear of retribution” were similarly rated the most challenging barriers.

Preworkshop, there were differences in perceived barriers to responding to microaggressions when participants’ responses were stratified by gender, URM status, or whether respondents had experienced or witnessed microaggressions (EMA) or not (NEMA). NEMA students reported significantly fewer challenges in responding to microaggressions compared to EMA students, including lower responses for “Not sure what to do or say” (overall $K = 1.42$, $p = .03$) and “Lack of familiarity with institutional support system” (overall $K = 1.51$, $p = .02$). NEMA students were less likely to cite “Not sure what to do or say” as a barrier, despite not having experienced a microaggression (66% reporting this as not at all challenging or slightly challenging compared to 21% of EMA respondents). NEMA students also thought “Fear of retribution” was not an important barrier, with 50% reporting this as not at all or slightly challenging compared to 18% of students who had experienced a microaggression, though the distributions did not appear to significantly differ (overall $K = 1.18$, $p = .12$).

Though Kolmogorov-Smirnov independent-samples tests revealed no significant differences in the distributions of respondents across gender and URM status on any items, there remained potentially noteworthy trends toward difference. URM

Copyright © 2020 Sandoval et al. This is an open-access publication distributed under the terms of the Creative Commons Attribution license.

Table 2. Reports of Having Witnessed Microaggressions in Preworkshop Surveys Among 121 Medical and Dental Student Survey Respondents

		URM ($n = 116$)			Female ($n = 112$)		
**Report**	**Overall No. (%)**	**No. (%)**	**OR (vs. Non-URM)**	**95% CI**	**No. (%)**	**OR (vs. Male)**	**95% CI**
At least one instance of:							
You experienced microaggressions	65 (53%)	26 (59%)	1.29	0.61-2.76	41 (70%)	3.51[a]	1.60-7.69
Colleague or peer experienced microaggressions	80 (66%)	29 (65%)	0.91	0.41-2.01	42 (72%)	1.54	0.70-3.43
Patient experienced microaggressions	62 (51%)	25 (56%)	1.32	0.62-2.80	38 (65%)	2.99[a]	1.38-6.45
Ever experienced any microaggressions	94 (77%)	36 (81%)	1.39	0.54-3.56	50 (86%)	2.40	0.93-6.25

Abbreviations: CI, confidence interval; OR, odds ratio; URM, underrepresented in medicine.
[a]$p < .01$.

students were more concerned about "Fear of retribution" (44% reported this as very challenging or extremely challenging compared to 21% of non-URM students). Similarly, male respondents were less likely to consider "Fear of retribution" to be a barrier (33% saying this was not at all challenging or slightly challenging compared to 20% of female respondents). Male respondents were also more likely to report lack of certainty around clinical relevance of microaggressions as a challenge (11% of female respondents reporting this as very or extremely challenging compared to 28% of male respondents).

Postworkshop Changes

Postworkshop, perceived challenges to addressing microaggressions and episodes of discrimination on the wards decreased for all of the barriers in the full sample and for all subgroups (for female respondents, URM respondents, and students who had experienced microaggressions), except "Fear of retribution," which remained a barrier for all respondents even after the workshop (Table 4). These improvements did not vary by subgroup in a statistically significant manner.

Discussion

To address the lack of existing curricula on microaggressions and discrimination in the clinical setting for medical trainees, we developed a 2-hour interactive workshop for preclinical medical and dental students. Our results demonstrate that participation in the workshop significantly improved students' self-perceived ability to recognize and respond to microaggressions; we also report a prevalence of students having witnessed or experienced at least one microaggression in the clinical setting, and we found self-identification as female to be associated with increased exposure to microaggressions.

Overall, this workshop significantly improved ($p = .003$) participant confidence in identifying and addressing microaggressions and discrimination in the clinical setting. The increase in participants' self-reported capability to recognize and respond to clinical microaggressions demonstrates that a 2-hour educational workshop can increase student confidence in handling these complex issues. We also found that the overwhelming majority of participants (77%) reported having encountered some form of a microaggression during a clinical encounter, with many having experienced one themselves, witnessed one towards a colleague, or witnessed one towards a patient. This statistic is particularly concerning given that participants surveyed were first-year medical and dental students with very limited clinical experience. This staggering majority also highlights the importance of developing curricula to help prepare students for addressing these encounters in the clinical setting. While no formal data on the prevalence of microaggressions in the clinical setting exist, several anecdotal summaries support our finding of their prevalence.[13,43,44] Additionally, existing frameworks for attendings and residents about how to manage and address these encounters further support the idea that the occurrence of microaggressions is prevalent enough to require formal training in how to address them.[26-29,33]

Table 3. Self-Reported Familiarity With Microaggressions and Ability to Define Microaggressions and Identify Microaggressions in a Case-Based Exercise

		Definition Correct		
**Group**	**Preworkshop Self-Reported Familiarity With Microaggressions: No. (%) Saying Very Good or Excellent**	**Preworkshop ($n = 121$): No. (%)**	**Postworkshop ($n = 101$): No. (%)**	**Changes in Paired Data ($n = 81$): p[a]**
Overall	56 (69%)	59 (48%)	53 (52%)	.38
URM	17 (63%)	16 (59%)	14 (51%)	.63
Female	27 (75%)	19 (52%)	24 (66%)	.18
EMA	43 (69%)	32 (52%)	35 (57%)	.61

Abbreviations: EMA, experienced microaggressions; URM, underrepresented in medicine.
[a]Based on McNemar's related samples test.

Copyright © 2020 Sandoval et al. This is an open-access publication distributed under the terms of the Creative Commons Attribution license.

Table 4. Mean Pre- and Postworkshop Medical and Dental Student Comfort With Addressing Microaggressions and Discrimination, With Tests of Change[a]

Types	Preworkshop *M*	Postworkshop *M*	p[b]
Microaggressions			
Fear of retribution			
Overall	3.32	3.29	.87
URM	3.44	3.26	.45
Female	3.51	3.49	>.99
EMA	3.50	3.41	.72
Difficulty recognizing			
Overall	2.51	1.79	<.001[e]
URM	2.22	1.70	.02[c]
Female	2.31	1.71	.001[d]
EMA	2.55	1.78	<.001[e]
Not sure what to say or do			
Overall	3.41	2.50	<.001[e]
URM	3.19	2.37	.02[c]
Female	3.51	2.51	<.001[e]
EMA	3.60	2.62	<.001[e]
Lack of allies			
Overall	2.97	2.55	.002[d]
URM	3.22	2.59	.02[c]
Female	3.00	2.54	.04[c]
EMA	3.09	2.67	.003[d]
Lack of familiarity with institutional support system			
Overall	2.97	2.02	<.001[e]
URM	2.96	1.96	.001[d]
Female	3.00	1.89	<.001[e]
EMA	2.90	2.12	<.001[e]
Lack of certainty of its clinical relevance			
Overall	2.46	1.85	<.001[e]
URM	2.36	1.73	.01[c]
Female	2.33	1.73	.02[c]
EMA	2.67	1.90	<.001[e]
Discrimination			
Fear of retribution			
Overall	2.96	3.01	.68
URM	3.12	2.85	.443
Female	3.11	3.28	.69
EMA	3.14	3.12	.88
Difficulty recognizing			
Overall	1.95	1.65	.003[d]
URM	1.92	1.42	.006[d]
Female	1.92	1.58	.02[c]
EMA	2.05	1.62	.001[d]
Not sure what to say or do			
Overall	2.95	2.24	<.001[e]
URM	2.88	1.96	<.001[e]
Female	3.08	2.39	<.001[e]
EMA	3.16	2.36	<.001[e]
Lack of allies			
Overall	2.64	2.19	.004[d]
URM	2.77	2.23	.05
Female	2.64	2.19	.08
EMA	2.76	2.28	.001[d]
Lack of familiarity with institutional support system			
Overall	2.01	1.95	<.001[e]
URM	1.92	1.81	.04[c]
Female	1.97	1.92	.007[d]
EMA	2.10	2.02	<.001[e]
Lack of certainty of its clinical relevance			
Overall	2.03	1.61	<.001[e]
URM	1.92	1.52	.04[c]
Female	1.97	1.46	.007[d]
EMA	2.15	1.65	<.001[e]

Abbreviations: EMA, experienced microaggressions; URM, underrepresented in medicine.
[a]Comparisons for non-URM, male, and NEMA (never experienced microaggressions) subgroups are not reported here.
[b]Value for Wilcoxon signed rank test comparing pre- and postworkshop.
[c]$p < .05$.
[d]$p < .01$.
[e]$p < .001$.

Copyright © 2020 Sandoval et al. This is an open-access publication distributed under the terms of the Creative Commons Attribution license.

Additionally, we found that those who reported female gender were more likely to report both experiencing and seeing microaggressions. This could be due to the fact that women, irrespective of their other intersectional identities, are more likely to experience gender-based differences in how they are treated by their medical faculty and colleagues.[45-47] Furthermore, given the increased burden of microaggressions for women, female students' lived experiences may predispose them to recognize microaggressions that they and others experience more easily than male students do.

We were also able to target some of the most prevalent perceived barriers to addressing microaggressions and moreover found that this workshop helped to decrease all of them—difficulty recognizing, not sure what to say or do, lack of allies, and lack of familiarity with institutional supports—except for fear of retribution. While the curriculum has been designed to help participants better recognize and address microaggressions, enumerate institutional support systems, and identify allies through the facilitators and fellow participants, this workshop does not explicitly guide participants through negotiating the perceived fear of retaliation. Medicine is hierarchically structured, and thus, for trainees, speaking up to call out social issues like microaggressions, regardless of the exact point of concern, has been reported to be one of the most significant challenges across clinical scenarios and physician career stages.[48,49] More explicitly, fear of retribution seems to be one of the most salient reasons medical students give for not reporting abuse they face on the wards.[50]

Our data further highlighted that URM students were more concerned about fear of retribution versus non-URM students. This finding is important to consider with our other finding that URM students are not more likely to report microaggressions. The assumption that URM students underreport because they less frequently experience microaggressions cannot be made given that it has been shown that people with marginalized identities are more likely to experience microaggressions.[18,19] What perhaps is leading to underreporting is instead fear of retribution. This significant barrier thus requires further investigation as to what interventions can be made to minimize it. Anecdotally, some potential interventions that could help include having an anonymous reporting system for students to report inappropriate behavior, discrimination, microaggressions, and so on.[51,52] Making the reporting system anonymous may decrease students' fear of retribution as their comments/reports will not be attached to their names. Other means include having a dedicated office or support staff for URM students; although this may not directly decrease the fear of retribution, it has been recommended in the literature to help health professional students feel more supported and empowered during their training.[53,54]

In terms of perceived challenges, a closer examination of the group that had never experienced a microaggression found that NEMA students underestimated the challenges of addressing microaggressions compared to those who had experienced microaggressions. NEMA students reported feeling less challenged by fear of retribution, allyship recognition, and surety of what to do. Additionally, our data imply that students in the NEMA group were more likely to be male and overrepresented in medicine rather than URM. These data demonstrate a lack of education surrounding microaggressions and discrimination—as a phenomenon to be understood and combatted—amongst NEMA students. In this vein, our workshop aimed to educate NEMA students to better understand the complexities of addressing microaggressions and discrimination. Furthermore, NEMA students statistically comprise the majority of clinical trainees and are more likely to occupy positions of power.[55,56] By educating NEMA students, future generations of clinicians can be expected to be more conscious of the impacts of microaggressions in clinical and everyday contexts. Among the many benefits that this increased consciousness could have on improving patient care is the reduction of the undue burden that microaggressions place on clinicians who identify as female and/or arise from underrepresented backgrounds.

Some of the more prominent scholarly critiques of microaggression training programs have asserted that education about microaggressions suppresses controversial speech and creates victimhood culture by oversensitizing groups of people often targeted by microaggressions.[57-59] Yet our finding that a majority of our respondents, even non-URM respondents, had experienced or witnessed microaggressions, including microaggressions affecting patients, suggests that such episodes are commonplace and clinically relevant. In one small group, for example, a white male participant described how a white patient refused to speak to a black classmate of his and how that negatively impacted the learning and clinical environment for everyone. Thus, rather than hypersensitizing students, this workshop provides actionable skills for situations they are already experiencing on the wards. Importantly, because we were able to make this workshop mandatory, it serves in particular to educate those who have never been the targets of these slights in the first place, as our data demonstrate that those least likely to experience microaggressions were most likely to underestimate the challenges that they pose.

Copyright © 2020 Sandoval et al. This is an open-access publication distributed under the terms of the Creative Commons Attribution license.

Common challenges in medical education studies, particularly those that use comparative survey analysis, include small sample size and absence of paired pre- and postintervention samples for statistical testing. Our cohort originally involved 163 students, with 121 (74%) completing the preworkshop survey and 81 paired postworkshop responses (66% of those who completed the preworkshop survey), which provided our study with a relatively high response rate that lost only 34% of preworkshop survey respondents to follow-up. In addition, we were able to validate the self-assessment of participants' comfort understanding and addressing microaggressions by comparing individuals' self-reported comfort with their success in identifying and responding to a microaggression case.

One limitation of this study is generalizability, as our sample is limited to first-year medical and dental students from only one institution. Furthermore, our study assessed the participants only in the short term before and after completing the workshop. At this point, we cannot ascertain the long-term impact of the workshop on participant confidence, self-perceived competence, or actual behavior in clinical settings. Another limitation in terms of implementation of the workshop is that the frameworks rely on guiding students towards institutional support systems and faculty who have the capacity to support students faced with these situations. Given this, institutional resources and faculty must be identified prior to implementing the workshop, along with recognition of where additional resources are needed.

Future directions for this workshop could include reassessing this cohort of participants after their core clinical experience to evaluate the sustainability of the workshop's impact and its ability to translate into behavioral change. Additional future directions could include revisions to the existing workshop, such as focusing on only one case to allow for more extensive discussions, changing cases to cover additional topics, and including active bystander training.[60] Additionally, more time could be spent on the role-play in the small groups so that students can strategize and better prepare goals for the discussions with their attending supervisors as well as practice those conversations. Finally, the workshop could be incorporated into simulations with standardized patients or objective structured clinical exams.

Appendices

A. PowerPoint Presentation.pptx

B. Cases.docx

C. Role Cards.docx

D. Framework Handout.docx

E. Facilitator Guide.docx

F. Abridged Facilitator Guide.docx

G. Preworkshop Survey.docx

H. Postworkshop Survey.docx

I. Text Exercise Criteria.docx

All appendices are peer reviewed as integral parts of the Original Publication.

Raquel Sofia Sandoval: Medical Student, Harvard Medical School

Titilayo Afolabi: Medical Student, Harvard Medical School

Jordan Said: Medical Student, Harvard Medical School

Spencer Dunleavy, MSc: Medical Student, Columbia University College of Physicians and Surgeons

Avik Chatterjee, MD, MPH: Physician, Boston Health Care for the Homeless Program; Associate Epidemiologist, Division of Global Health Equity, Brigham and Women's Hospital; Instructor, Harvard Medical School

Daniele Ölveczky, MD, MS: Assistant Professor of Medicine, Harvard Medical School; Inclusion Officer, Department of Medicine, Beth Israel Deaconess Medical Center

Acknowledgments
We would like to acknowledge the following for their support in the development of the workshop: Dr. Anita Cheng, Gilianne Jawahir, Dr. Barbara Cockrill, Dr. John Dalrymple, Dean Fidencio Saldaña, and Dean Edward Hundert. We would also like to express our gratitude to Dr. Amy Sullivan from the Carl J. Shapiro Institute for Education and Research at Beth Israel Deaconess Center for her expert advice and feedback on the creation of our surveys.

Disclosures
None to report.

Funding/Support
None to report.

Prior Presentations
Sandoval RS, Chatterjee A. Building a toolkit for residents: addressing microaggressions and discrimination on the wards. Pilot workshop presented at: Cambridge Health Alliance; October 7, 2018; Boston, MA.

Sandoval RS, Said J, Olveczky D. Building a toolkit for residents: addressing microaggressions and discrimination on the wards. Poster presented at: The Academy at HMS Medical Education Day; December 7, 2018; Boston, MA.

Copyright © 2020 Sandoval et al. This is an open-access publication distributed under the terms of the Creative Commons Attribution license.

Said J. Building a toolkit for residents: addressing microaggressions and discrimination on the wards. Poster presented at: Q-Med: Building LGBTQI+ Leaders in Health Care; March 30-31, 2019; New Haven, CT.

Sandoval RS, Said J. Building a toolkit for residents: addressing microaggressions and discrimination on the wards. Poster presented at: Cambridge Health Alliance Academic Poster Session; May 2, 2019; Cambridge, MA.

Said J, Chatterjee A. Building a toolkit for residents: addressing microaggressions and discrimination on the wards Poster presented at: 2019 SGIM Annual Meeting; May 8-11, 2019; Washington, DC.

Ethical Approval

The Harvard Medical School Institutional Review Board approved this study.

References

1. Almond D, Chay KY, Greenstone M. Civil rights, the war on poverty, and black-white convergence in infant mortality in the rural South and Mississippi. MIT Department of Economics Working Paper No. 07-04. SSRN website. https://papers.ssrn.com/sol3/papers.cfm?abstract_id=961021. Published February 7, 2007.
2. Chapman EN, Kaatz A, Carnes M. Physicians and implicit bias: how doctors may unwittingly perpetuate health care disparities. *J Gen Intern Med*. 2013;28(11):1504-1510. https://doi.org/10.1007/s11606-013-2441-1
3. Nelson AR. Unequal treatment: report of the Institute of Medicine on racial and ethnic disparities in healthcare. *Ann Thorac Surg*. 2003;76(4):S1377-S1381. https://doi.org/10.1016/S0003-4975(03)01205-0
4. Khan A, Plummer D, Hussain R, Minichiello V. Does physician bias affect the quality of care they deliver? Evidence in the care of sexually transmitted infections. *Sex Transm Infect*. 2008;84(2):150-151. https://doi.org/10.1136/sti.2007.028050
5. Green AR, Carney DR, Pallin DJ, et al. Implicit bias among physicians and its prediction of thrombolysis decisions for black and white patients. *J Gen Intern Med*. 2007;22(9):1231-1238. https://doi.org/10.1007/s11606-007-0258-5
6. Quinn GP, Vadaparampil ST, King L, et al. Impact of physicians' personal discomfort and patient prognosis on discussion of fertility preservation with young cancer patients. *Patient Educ Couns*. 2009;77(3):338-343. https://doi.org/10.1016/j.pec.2009.09.007
7. Sabin JA, Riskind RG, Nosek BA. Health care providers' implicit and explicit attitudes toward lesbian women and gay men. *Am J Public Health*. 2015;105(9):1831-1841. https://doi.org/10.2105/AJPH.2015.302631
8. Kripalani S, Bussey-Jones J, Katz MG, Genao I. A prescription for cultural competence in medical education. *J Gen Intern Med*. 2006;21(10):1116-1120. https://doi.org/10.1111/j.1525-1497.2006.00557.x
9. White AA III, Logghe HJ, Goodenough DA, et al. Self-awareness and cultural identity as an effort to reduce bias in medicine. *J Racial Ethn Health Disparities*. 2018;5(1):34-49. https://doi.org/10.1007/s40615-017-0340-6
10. Yeager KA, Bauer-Wu S. Cultural humility: essential foundation for clinical researchers. *Appl Nurs Res*. 2013;26(4):251-256. https://doi.org/10.1016/j.apnr.2013.06.008
11. Brooks KC, Rougas S, George P. When race matters on the wards: talking about racial health disparities and racism in the clinical setting. *MedEdPORTAL*. 2016;12:10523. https://doi.org/10.15766/mep_2374-8265.10523
12. Sue DW, Capodilupo CM, Torino GC, et al. Racial microaggressions in everyday life: implications for clinical practice. *Am Psychol*. 2007;62(4):271-286. https://doi.org/10.1037/0003-066X.62.4.271
13. Montenegro RE. My name is not "interpreter." *JAMA*. 2016;315(19):2071-2072. https://doi.org/10.1001/jama.2016.1249
14. Bowen EA, Murshid NS. Trauma-informed social policy: a conceptual framework for policy analysis and advocacy. *Am J Public Health*. 2016;106(2):223-229. https://doi.org/10.2105/AJPH.2015.302970
15. Pascoe EA, Smart Richman L. Perceived discrimination and health: a meta-analytic review. *Psychol Bull*. 2009;135(4):531-554. https://doi.org/10.1037/a0016059
16. Huynh VW. Ethnic microaggressions and the depressive and somatic symptoms of Latino and Asian American adolescents. *J Youth Adolesc*. 2012;41(7):831-846. https://doi.org/10.1007/s10964-012-9756-9
17. Maslach C, Jackson SE. The measurement of experienced burnout. *J Organ Behav*. 1981;2(2):99-113. https://doi.org/10.1002/job.4030020205
18. Cheng S-JA. A review of "That's So Gay! Microaggressions and the Lesbian, Gay, Bisexual, and Transgender Community." *J GLBT Fam Stud*. 2014;10(4):422-424. https://doi.org/10.1080/1550428X.2014.897919
19. Sue DW, ed. *Microaggressions and Marginality: Manifestation, Dynamics, and Impact*. Hoboken, NJ; Wiley: 2010.
20. FitzGerald C, Hurst S. Implicit bias in healthcare professionals: a systematic review. *BMC Med Ethics*. 2017;18:19. https://doi.org/10.1186/s12910-017-0179-8
21. Blair IV, Steiner JF, Fairclough DL, et al. Clinicians' implicit ethnic/racial bias and perceptions of care among black and Latino patients. *Ann Fam Med*. 2013;11(1):43-52. https://doi.org/10.1370/afm.1442
22. Cooper LA, Roter DL, Carson KA, et al. The associations of clinicians' implicit attitudes about race with medical visit communication and patient ratings of interpersonal care. *Am J Public Health*. 2012;102(5):979-987. https://doi.org/10.2105/AJPH.2011.300558

Copyright © 2020 Sandoval et al. This is an open-access publication distributed under the terms of the Creative Commons Attribution license.

23. Epstein RM, Krasner MS. Physician resilience: what it means, why it matters, and how to promote it. *Acad Med*. 2013;88(3): 301-303. https://doi.org/10.1097/ACM.0b013e318280cff0

24. Bird A, Pincavage A. A curriculum to foster resident resilience. *MedEdPORTAL*. 2016;12:10439. https://doi.org/10.15766/mep_2374-8265.10439

25. Osseo-Asare A, Balasuriya L, Huot SJ, et al. Minority resident physicians' views on the role of race/ethnicity in their training experiences in the workplace. *JAMA Netw Open*. 2018;1(5): e182723. https://doi.org/10.1001/jamanetworkopen.2018.2723

26. Whitgob EE, Blankenburg RL, Bogetz AL. The discriminatory patient and family: strategies to address discrimination towards trainees. *Acad Med*. 2016;91(11):S64-S69. https://doi.org/10.1097/ACM.0000000000001357

27. Paul-Emile K, Smith AK, Lo B, Fernández A. Dealing with racist patients. *N Engl J Med*. 2016;374(8):708-711. https://doi.org/10.1056/NEJMp1514939

28. Mostow C, Crosson J, Gordon S, et al. Treating and precepting with RESPECT: a relational model addressing race, ethnicity, and culture in medical training. *J Gen Intern Med*. 2010;25(suppl 2): S146-S154. https://doi.org/10.1007/s11606-010-1274-4

29. Wheeler DJ, Zapata J, Davis D, Chou C. Twelve tips for responding to microaggressions and overt discrimination: when the patient offends the learner. *Med Teach*. 2019;41(10): 1112-1117. https://doi.org/10.1080/0142159X.2018.1506097

30. Martinchek M, Bird A, Pincavage AT. Building team resilience and debriefing after difficult clinical events: a resilience curriculum for team leaders. *MedEdPORTAL*. 2017;13:10601. https://doi.org/10.15766/mep_2374-8265.10601

31. Potter LA, Burnett-Bowie SM, Potter J. Teaching medical students how to ask patients questions about identity, intersectionality, and resilience. *MedEdPORTAL*. 2016;12:10422. https://doi.org/10.15766/mep_2374-8265.10422

32. Lee W. Maintaining medical professionalism: promoting balance and preventing student burnout. *MedEdPORTAL*. 2014;10:9878. https://doi.org/10.15766/mep_2374-8265.9878

33. Stop, talk, roll. Georgetown University School of Medicine website. https://som.georgetown.edu/diversityandinclusion/stoptalkroll. Published 2018.

34. Dubey S, Dubey AK. Promotion of higher order of cognition in undergraduate medical students using case-based approach. *J Educ Health Promot*. 2017;6:75.

35. Hashim R, Azam N, Shafi M, Majeed S, Ali S. Perceptions of undergraduate medical students regarding case based learning and tutorial format. *J Pak Med Assoc*. 2015;65(10):1050-1055.

36. Reed S, Shell R, Kassis K, et al. Applying adult learning practices in medical education. *Curr Probl Pediatr Adolesc Health Care*. 2014;44(6):170-181. https://doi.org/10.1016/j.cppeds.2014.01.008

37. Museus SD, Kiang PN. Deconstructing the model minority myth and how it contributes to the invisible minority reality in higher education research. *New Dir Inst Res*. 2009;(142):5-15. https://doi.org/10.1002/ir.292

38. Review of: Chou RS, Feagin JR. *The Myth of the Model Minority: Asian Americans Facing Racism*. *Contemp Sociol*. 2011;40(1): 106. https://doi.org/10.1177/0094306110392157b

39. Rodríguez JE, Campbell KM, Pololi LH. Addressing disparities in academic medicine: what of the minority tax? *BMC Med Educ*. 2015;16:6. https://doi.org/10.1186/s12909-015-0290-9

40. Nadal KL. The Racial and Ethnic Microaggressions Scale (REMS): construction, reliability, and validity. *J Couns Psychol*. 2011;58(4): 470-480. https://doi.org/10.1037/a0025193

41. Torres-Harding SR, Andrade AL Jr, Romero Diaz CE. The Racial Microaggressions Scale (RMAS): a new scale to measure experiences of racial microaggressions in people of color. *Cultur Divers Ethnic Minor Psychol*. 2012;18(2):153-164. https://doi.org/10.1037/a0027658

42. Underrepresented in medicine definition. Association of American Medical Colleges website. https://www.aamc.org/what-we-do/mission-areas/diversity-inclusion/underrepresented-in-medicine. Published 2004.

43. Cyrus KD, Angoff NR, Illuzzi JL, Schwartz ML, Wilkins KM. When patients hurt us. *Med Teach*. 2018;40(12):1308-1309. https://doi.org/10.1080/0142159X.2018.1428291

44. Bynum WE, Lindeman B. Caught in the middle: a resident perspective on influences from the learning environment that perpetuate mistreatment. *Acad Med*. 2016;91(3):301-304. https://doi.org/10.1097/ACM.0000000000001058

45. Babaria P, Bernheim S, Nunez-Smith M. Gender and the pre-clinical experiences of female medical students: a taxonomy. *Med Educ*. 2011;45(3):249-260. https://doi.org/10.1111/j.1365-2923.2010.03856.x

46. Hill E, Vaughan S. The only girl in the room: how paradigmatic trajectories deter female students from surgical careers. *Med Educ*. 2013;47(6):547-556. https://doi.org/10.1111/medu.12134

47. Hinze SW. "Am I being over-sensitive?" Women's experience of sexual harassment during medical training. *Health (London)*. 2004;8(1):101-127. https://doi.org/10.1177/1363459304038799

48. Srivastava R. Speaking up—when doctors navigate medical hierarchy. *N Engl J Med*. 2013;368(4):302-305. https://doi.org/10.1056/NEJMp1212410

49. Martinez W, Etchegaray JM, Thomas EJ, et al. "Speaking up" about patient safety concerns and unprofessional behaviour among residents: validation of two scales. *BMJ Qual Saf*. 2015;24(11):671-680. https://doi.org/10.1136/bmjqs-2015-004253

Copyright © 2020 Sandoval et al. This is an open-access publication distributed under the terms of the Creative Commons Attribution license.

50. Elnicki DM, Curry RH, Fagan M, et al. Medical students' perspectives on and responses to abuse during the internal medicine clerkship. *Teach Learn Med*. 2002;14(2):92-97. https://doi.org/10.1207/S15328015TLM1402_05

51. Anonymous Reporting Hotline. Harvard University website. https://reportinghotline.harvard.edu

52. Harvard Medical School Student Handbook. 4.08. Policy and procedures for consideration of unprofessional conduct. Harvard Medical School website. https://medstudenthandbook.hms.harvard.edu/408-policy-and-procedures-consideration-unprofessional-conduct. Updated August 21, 2017.

53. White C, Louis B, Persky A, et al. Institutional strategies to achieve diversity and inclusion in pharmacy education. *Am J Pharm Educ*. 2013;77(5):97.

54. Young ME, Thomas A, Varpio L, et al. Facilitating admissions of diverse students: a six-point, evidence-informed framework for pipeline and program development. *Perspect Med Educ*. 2017;6(2):82-90. https://doi.org/10.1007/s40037-017-0341-5

55. Total U.S. medical school enrollment by race/ethnicity and sex, 2014-2015 through 2018-2019. Association of American Medical Colleges website. https://www.aamc.org/data/facts/enrollmentgraduate/. Published 2018.

56. Carr PL, Raj A, Kaplan SE, Terrin N, Breeze JL, Freund KM. Gender differences in academic medicine: retention, rank, and leadership comparisons from the National Faculty Survey. *Acad Med*. 2018;93(11):1694-1699. https://doi.org/10.1097/ACM.0000000000002146

57. Thomas KR. Macrononsense in multiculturalism. *Am Psychol*. 2008;63(4):274-275. https://doi.org/10.1037/0003-066X.63.4.274

58. Lukianoff G, Haidt J. The coddling of the American mind. *Atlantic*. September 2015. https://www.theatlantic.com/magazine/archive/2015/09/the-coddling-of-the-american-mind/399356/

59. Powers K. *The Silencing: How the Left Is Killing Free Speech*. Washington, DC: Regnery Publishing; 2015.

60. Tenney L. Being an active bystander: strategies for challenging the emergence of bias. Kirwan Institute for the Study of Race and Ethnicity website. http://kirwaninstitute.osu.edu/wp-content/uploads/2018/07/Being-an-Active-Bystander-2017.pdf. Published 2017.

Received: April 2, 2019
Accepted: September 30, 2019
Published: April 3, 2020

Copyright © 2020 Sandoval et al. This is an open-access publication distributed under the terms of the Creative Commons Attribution license.
