# Supplementary material for: Building a Tool Kit for Medical and Dental Students: Addressing Microaggressions and Discrimination on the Wards
Source: MedEdPORTAL. 2020 Apr 3;16:10893. doi: 10.15766/mep_2374-8265.10893 (PMC7187912; doi:10.15766/mep_2374-8265.10893)
Supplement: Supplementary file 1 — PowerPoint Presentation.pptxCases.docxRole Cards.docxFramework Handout.docxFacilitator Guide.docxAbridged Facilitator Guide.docxPreworkshop Survey.docxPostworkshop Survey.docxText Exercise Criteria.docx [file mep-16-10893-s001.zip › A. PowerPoint Presentation.pptx]

## Slide 1
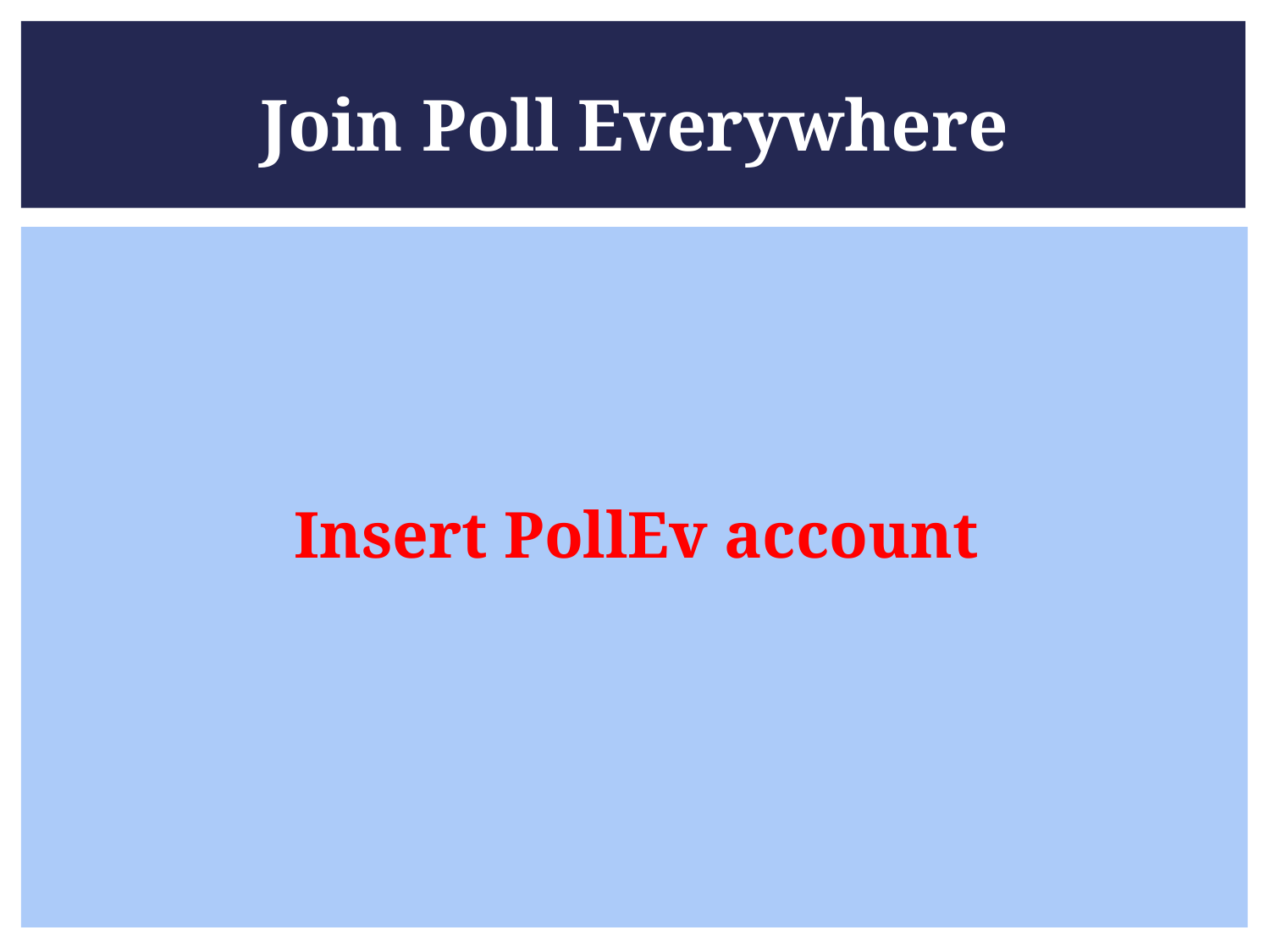

# Join Poll Everywhere
Insert PollEv account

## Slide 2
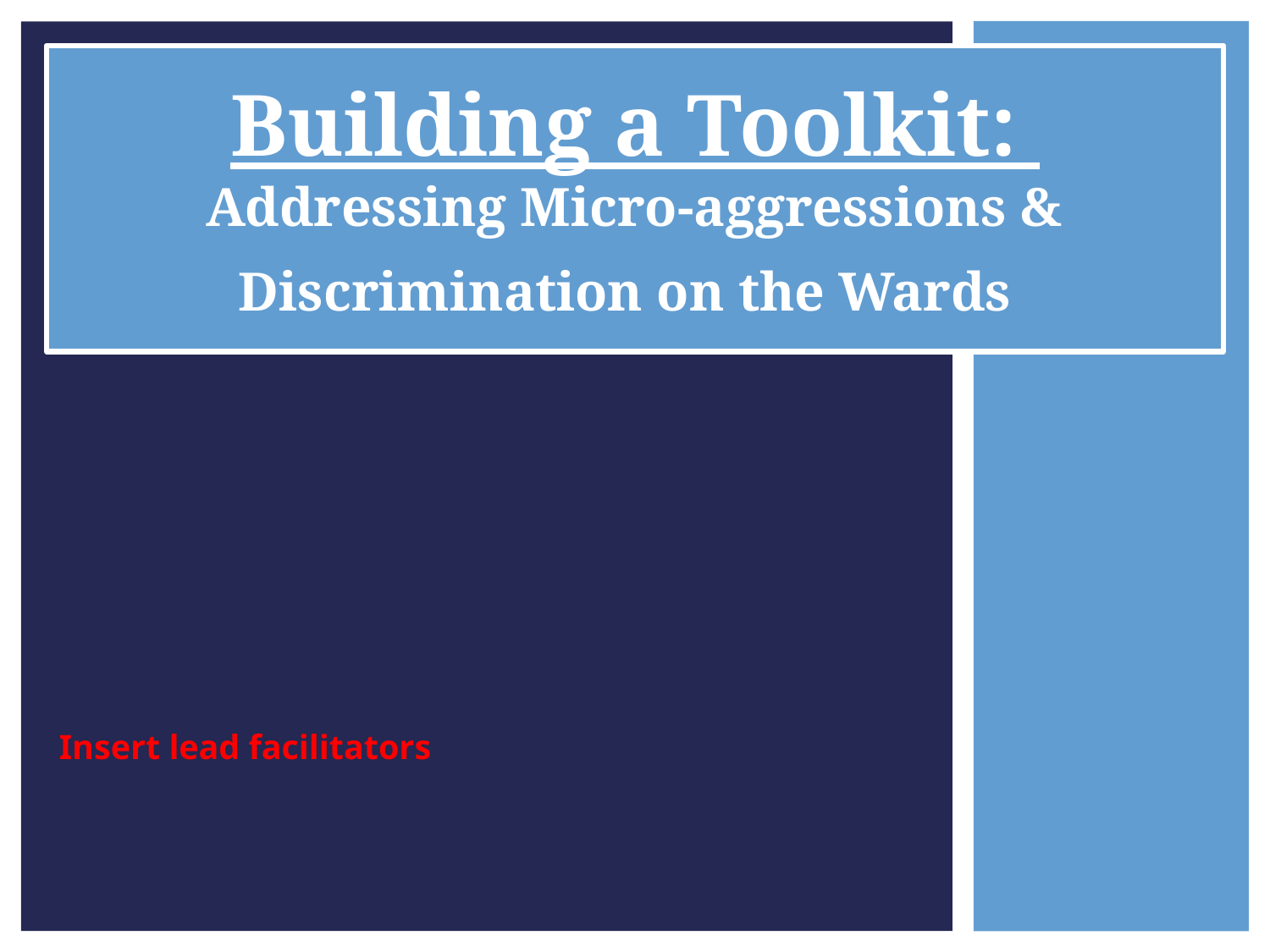

# Building a Toolkit:
Addressing Micro-aggressions & Discrimination on the Wards
Insert lead facilitators

## Slide 3
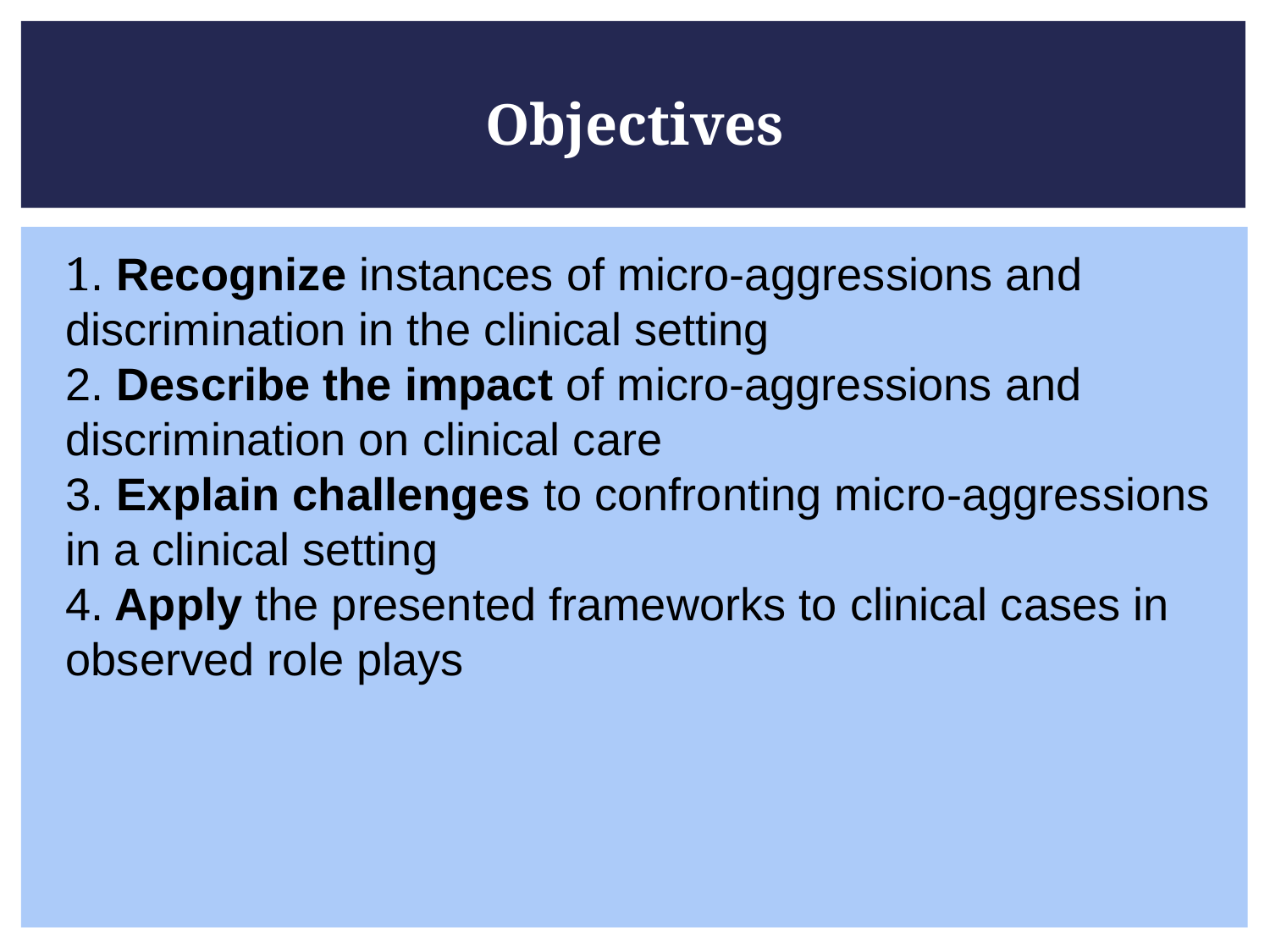

# Objectives
1. Recognize instances of micro-aggressions and discrimination in the clinical setting
2. Describe the impact of micro-aggressions and discrimination on clinical care
3. Explain challenges to confronting micro-aggressions in a clinical setting
4. Apply the presented frameworks to clinical cases in observed role plays

## Slide 4
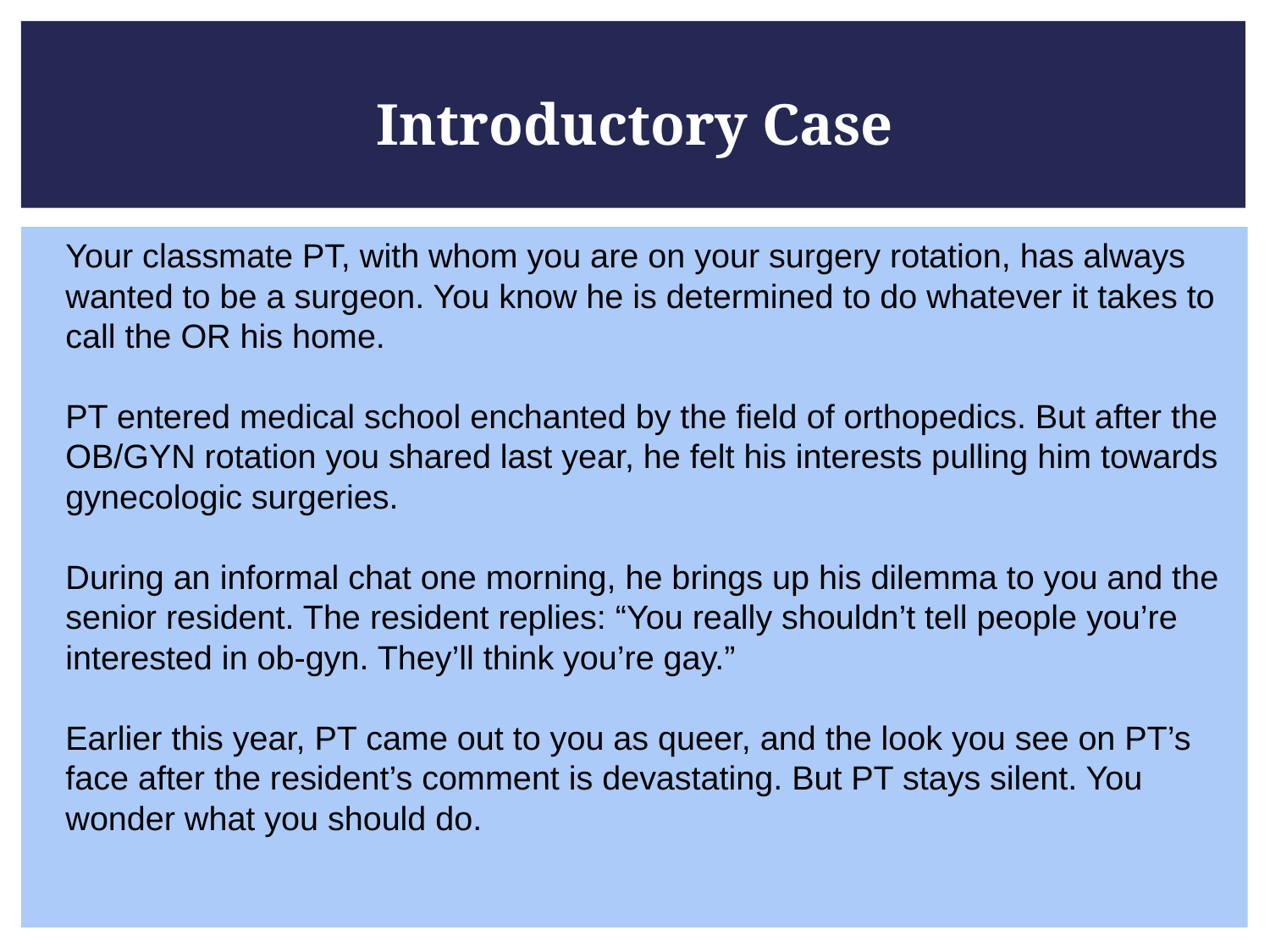

# Introductory Case
Your classmate PT, with whom you are on your surgery rotation, has always wanted to be a surgeon. You know he is determined to do whatever it takes to call the OR his home.
PT entered medical school enchanted by the field of orthopedics. But after the OB/GYN rotation you shared last year, he felt his interests pulling him towards gynecologic surgeries.
During an informal chat one morning, he brings up his dilemma to you and the senior resident. The resident replies: “You really shouldn’t tell people you’re interested in ob-gyn. They’ll think you’re gay.”
Earlier this year, PT came out to you as queer, and the look you see on PT’s face after the resident’s comment is devastating. But PT stays silent. You wonder what you should do.

## Slide 5
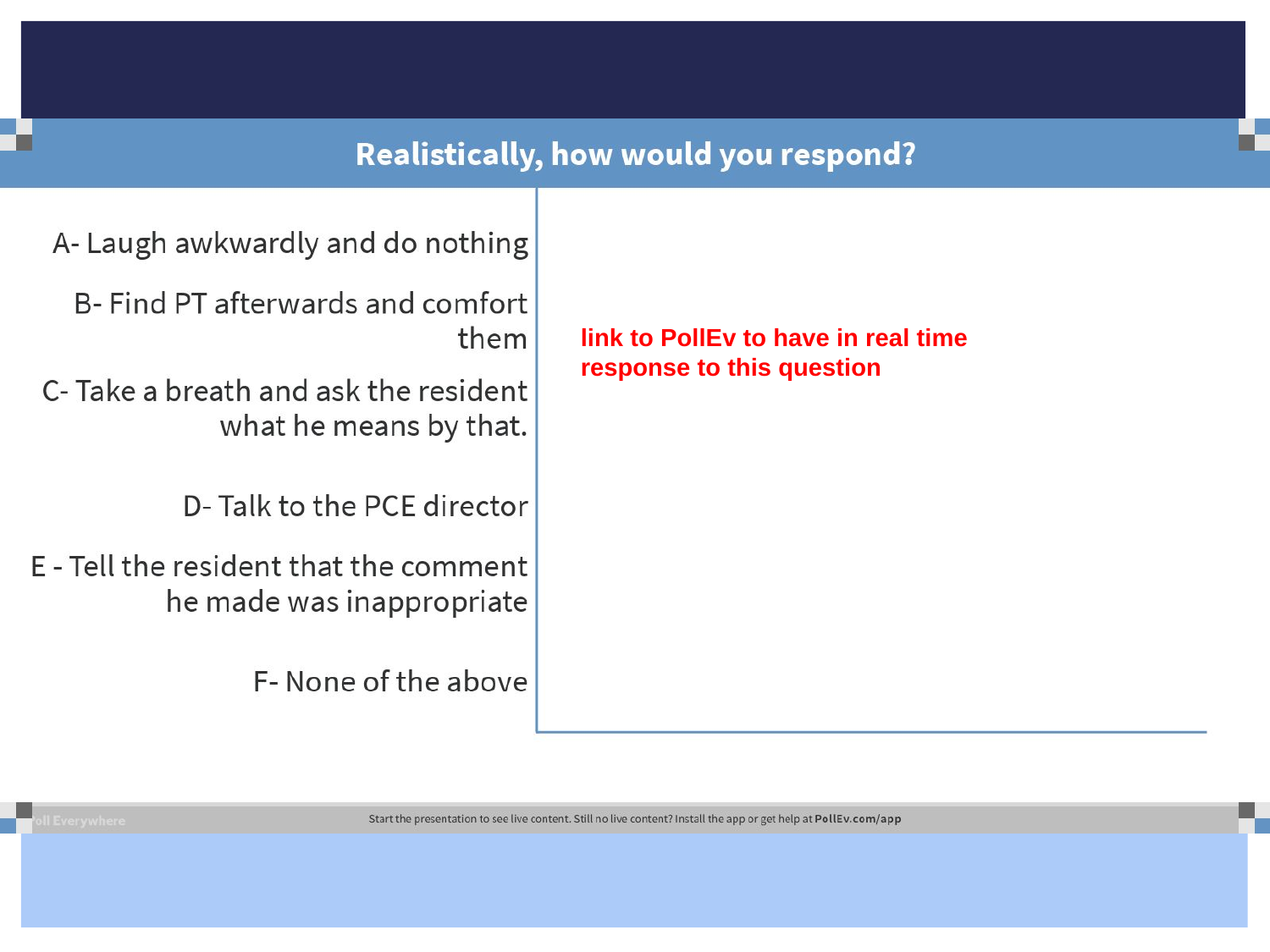

link to PollEv to have in real time response to this question

## Slide 6
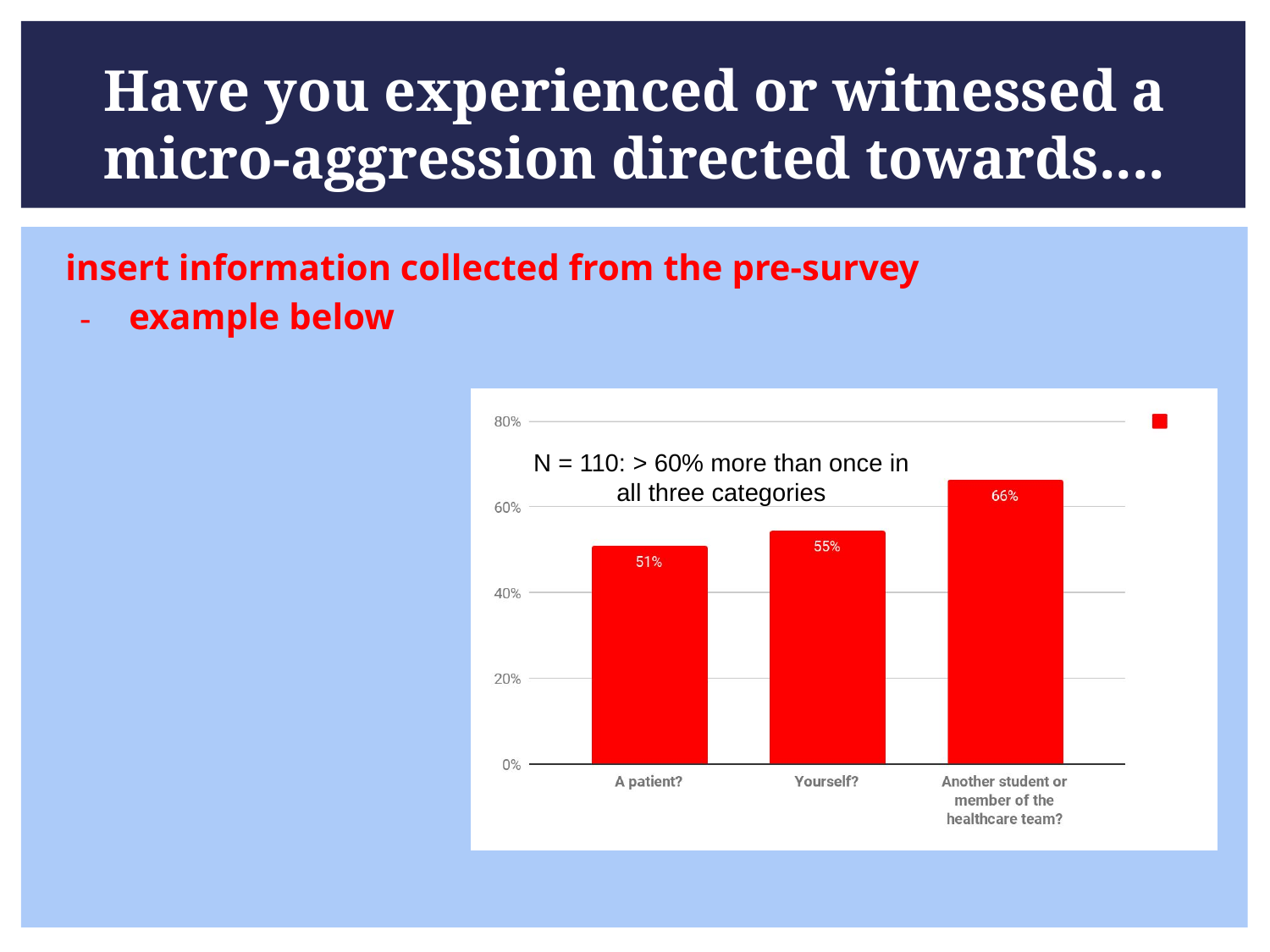

# Have you experienced or witnessed a micro-aggression directed towards....
insert information collected from the pre-survey
example below
N = 110: > 60% more than once in all three categories

## Slide 7
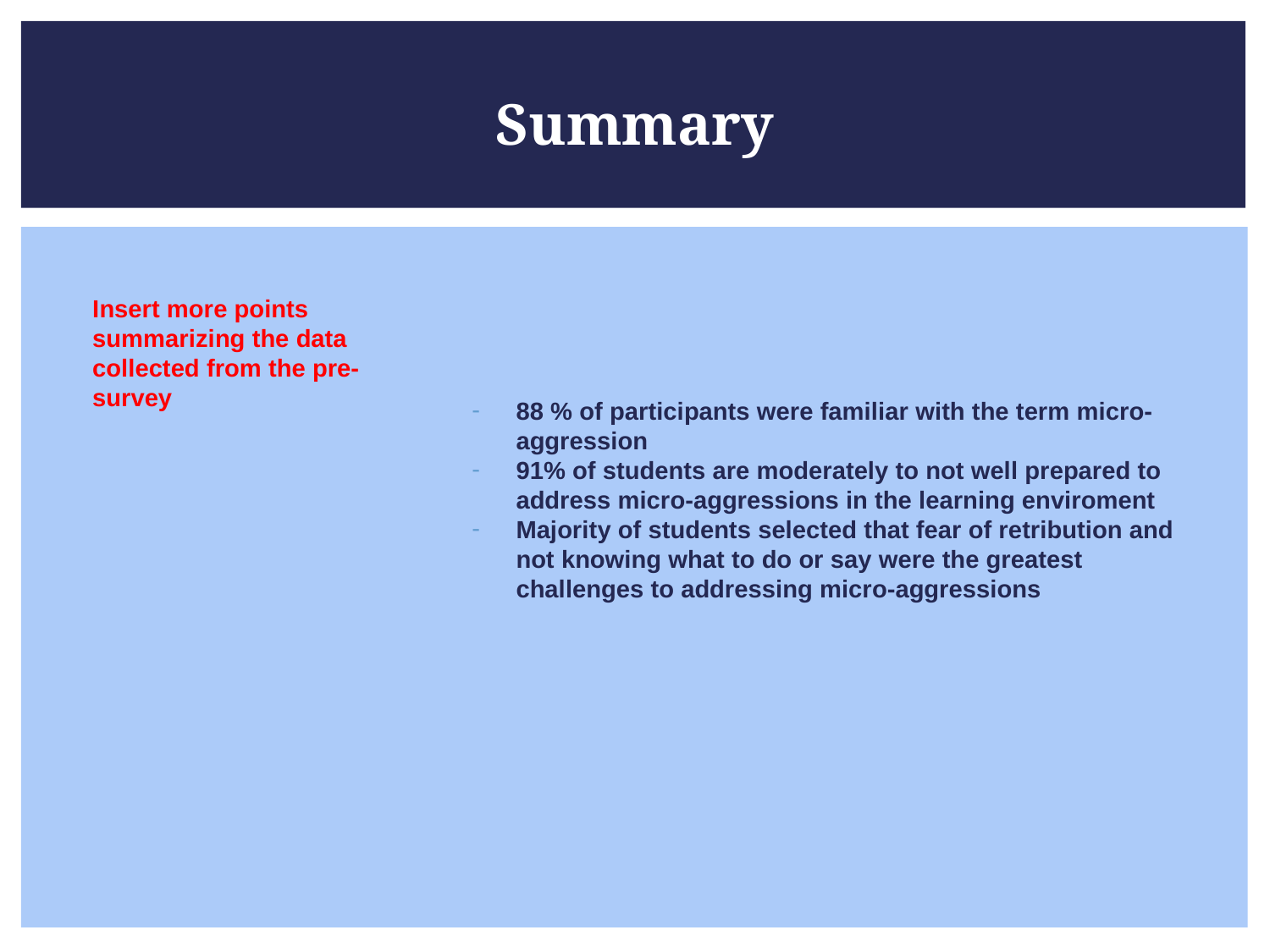

# Summary
Insert more points summarizing the data collected from the pre-survey
88 % of participants were familiar with the term micro-aggression
91% of students are moderately to not well prepared to address micro-aggressions in the learning enviroment
Majority of students selected that fear of retribution and not knowing what to do or say were the greatest challenges to addressing micro-aggressions

## Slide 8
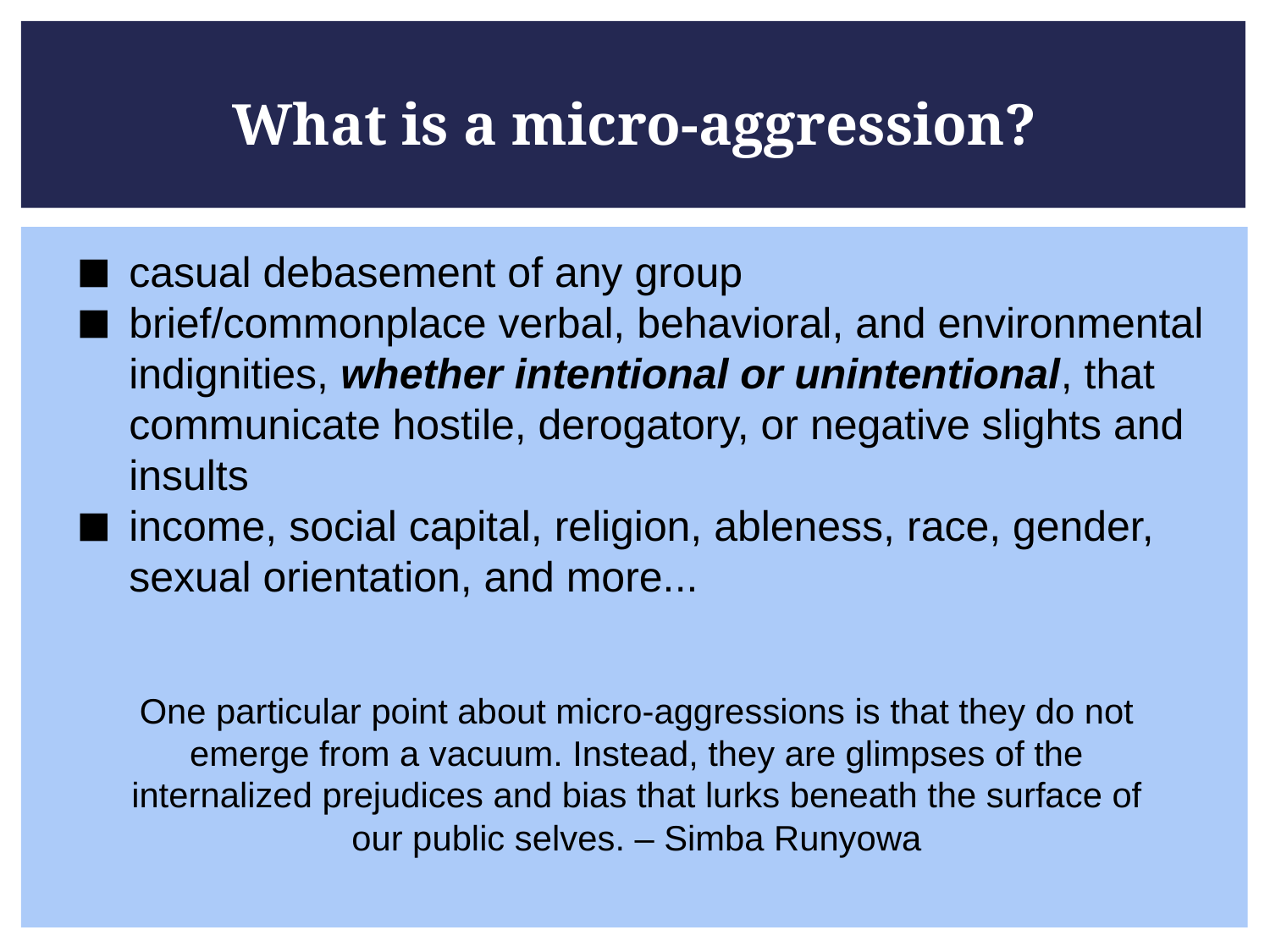

# What is a micro-aggression?
casual debasement of any group
brief/commonplace verbal, behavioral, and environmental indignities, whether intentional or unintentional, that communicate hostile, derogatory, or negative slights and insults
income, social capital, religion, ableness, race, gender, sexual orientation, and more...
One particular point about micro-aggressions is that they do not emerge from a vacuum. Instead, they are glimpses of the internalized prejudices and bias that lurks beneath the surface of our public selves. – Simba Runyowa

## Slide 9
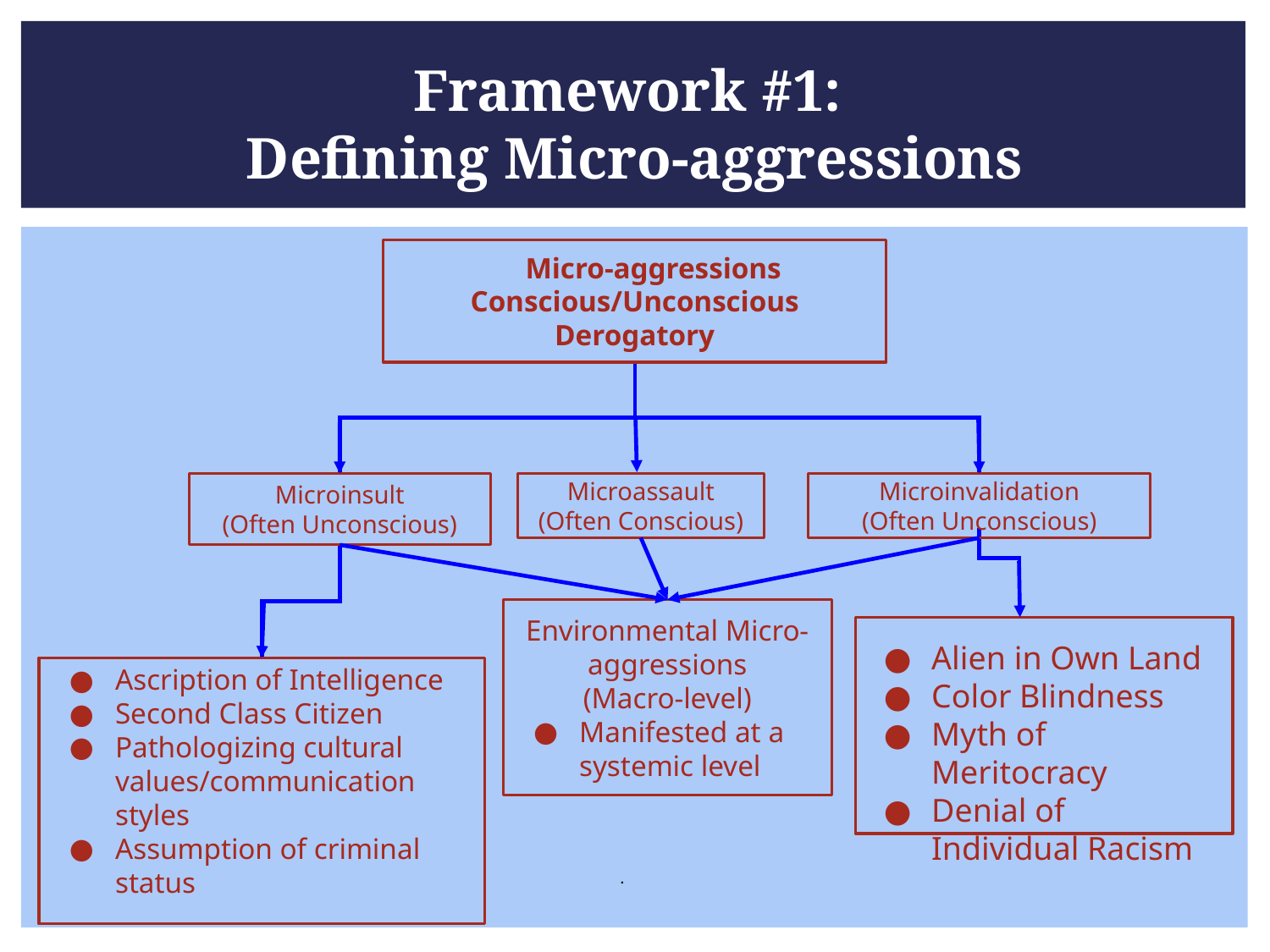

# Framework #1:
Defining Micro-aggressions
 Micro-aggressions
Conscious/Unconscious
Derogatory
Microinsult
(Often Unconscious)
Microassault
(Often Conscious)
Microinvalidation
(Often Unconscious)
Environmental Micro-aggressions
(Macro-level)
Manifested at a systemic level
Alien in Own Land
Color Blindness
Myth of Meritocracy
Denial of Individual Racism
Ascription of Intelligence
Second Class Citizen
Pathologizing cultural values/communication styles
Assumption of criminal status
.

## Slide 10
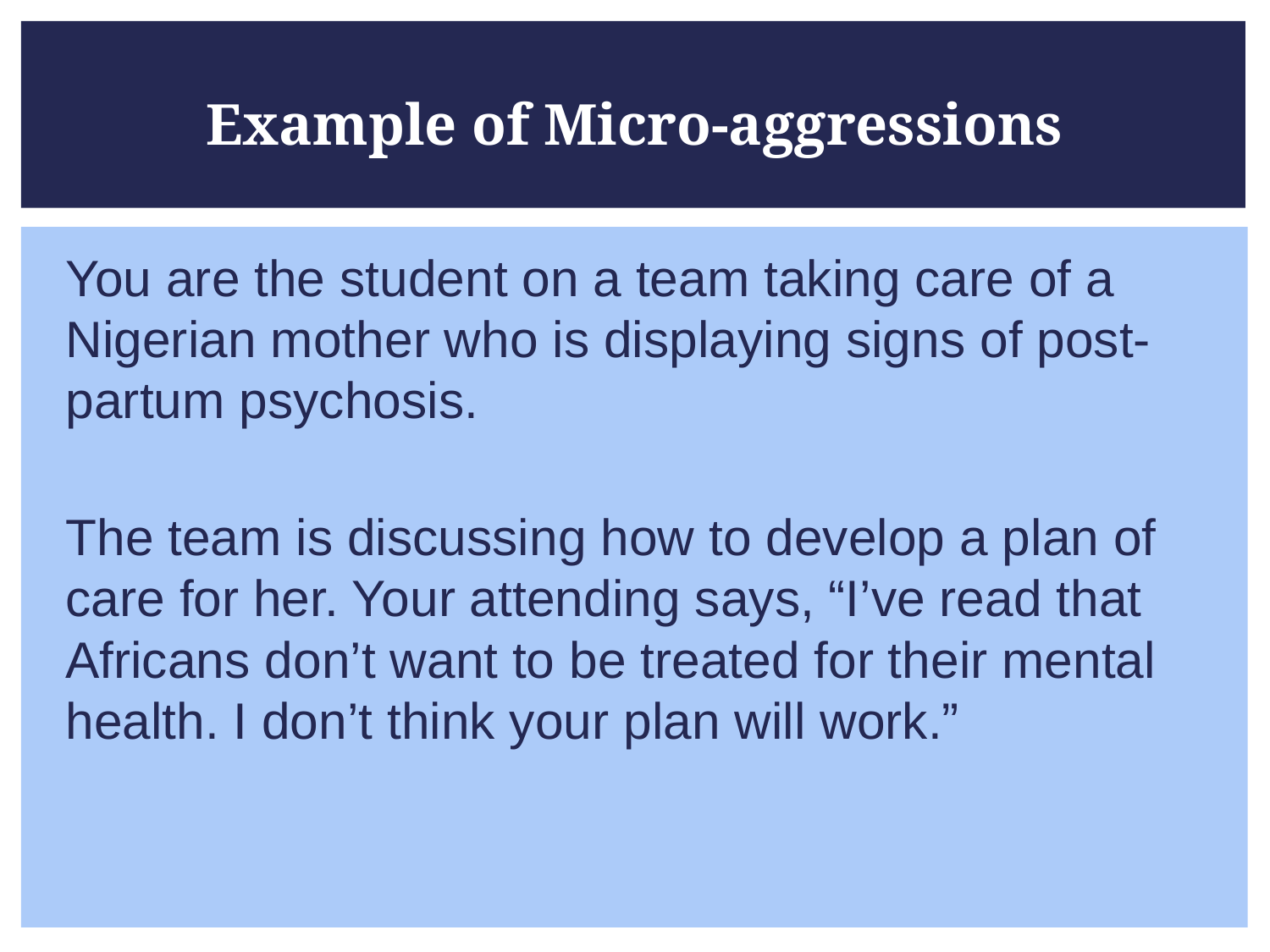

# Example of Micro-aggressions
You are the student on a team taking care of a Nigerian mother who is displaying signs of post-partum psychosis.
The team is discussing how to develop a plan of care for her. Your attending says, “I’ve read that Africans don’t want to be treated for their mental health. I don’t think your plan will work.”

## Slide 11
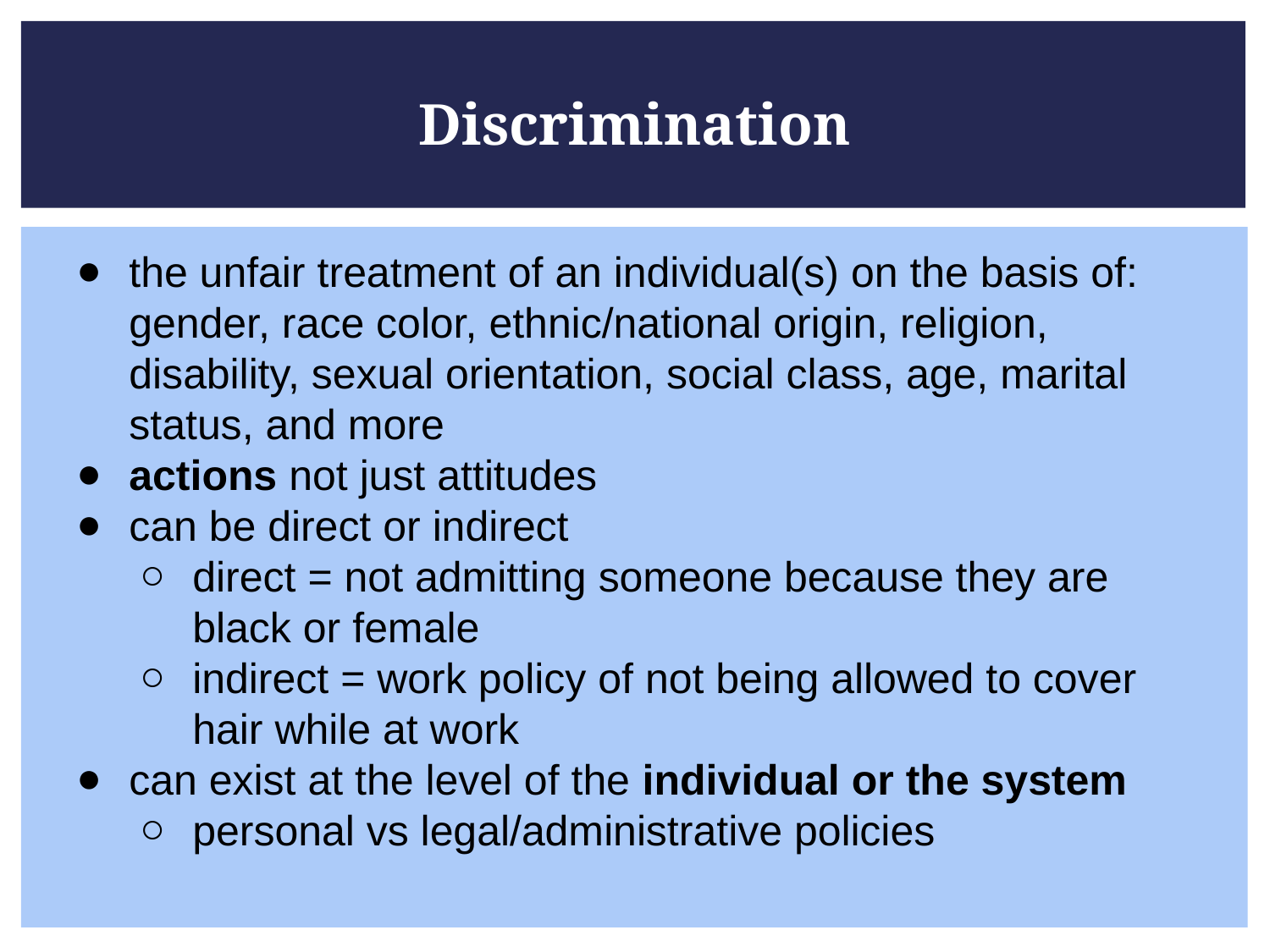

# Discrimination
the unfair treatment of an individual(s) on the basis of: gender, race color, ethnic/national origin, religion, disability, sexual orientation, social class, age, marital status, and more
actions not just attitudes
can be direct or indirect
direct = not admitting someone because they are black or female
indirect = work policy of not being allowed to cover hair while at work
can exist at the level of the individual or the system
personal vs legal/administrative policies

## Slide 12
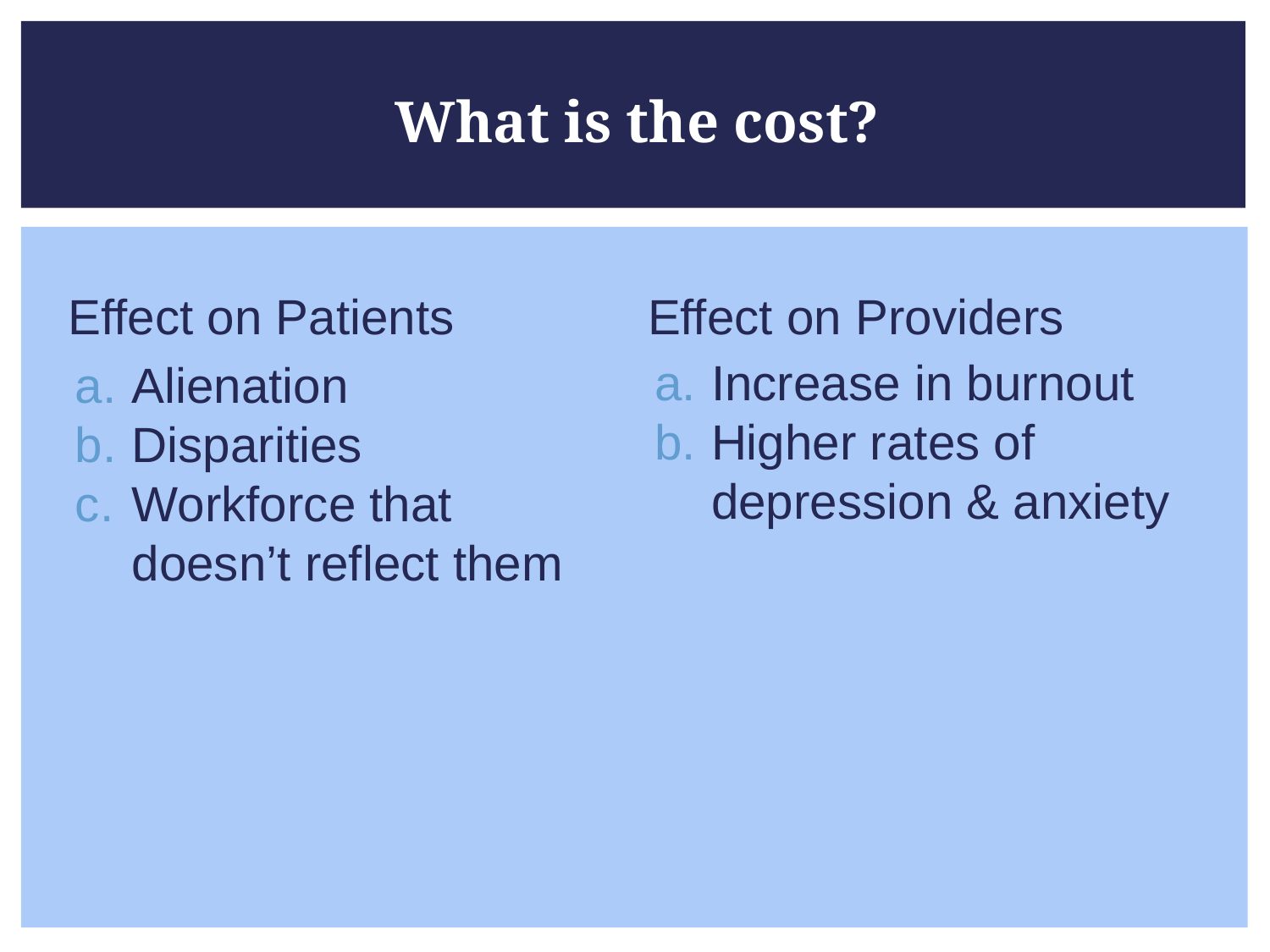

# What is the cost?
Effect on Providers
Increase in burnout
Higher rates of depression & anxiety
Effect on Patients
Alienation
Disparities
Workforce that doesn’t reflect them

## Slide 13
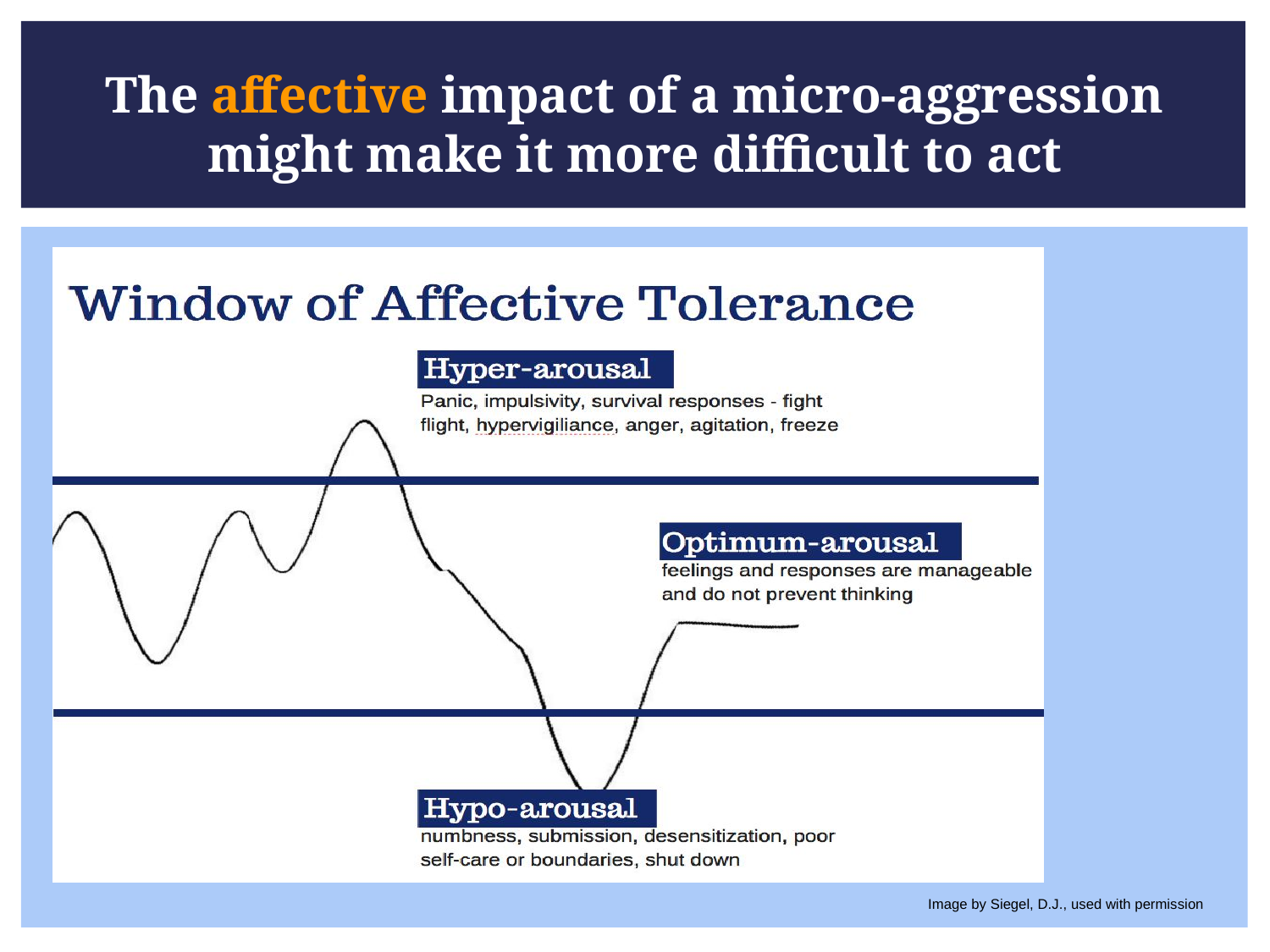

# The affective impact of a micro-aggression might make it more difficult to act
Image by Siegel, D.J., used with permission

## Slide 14
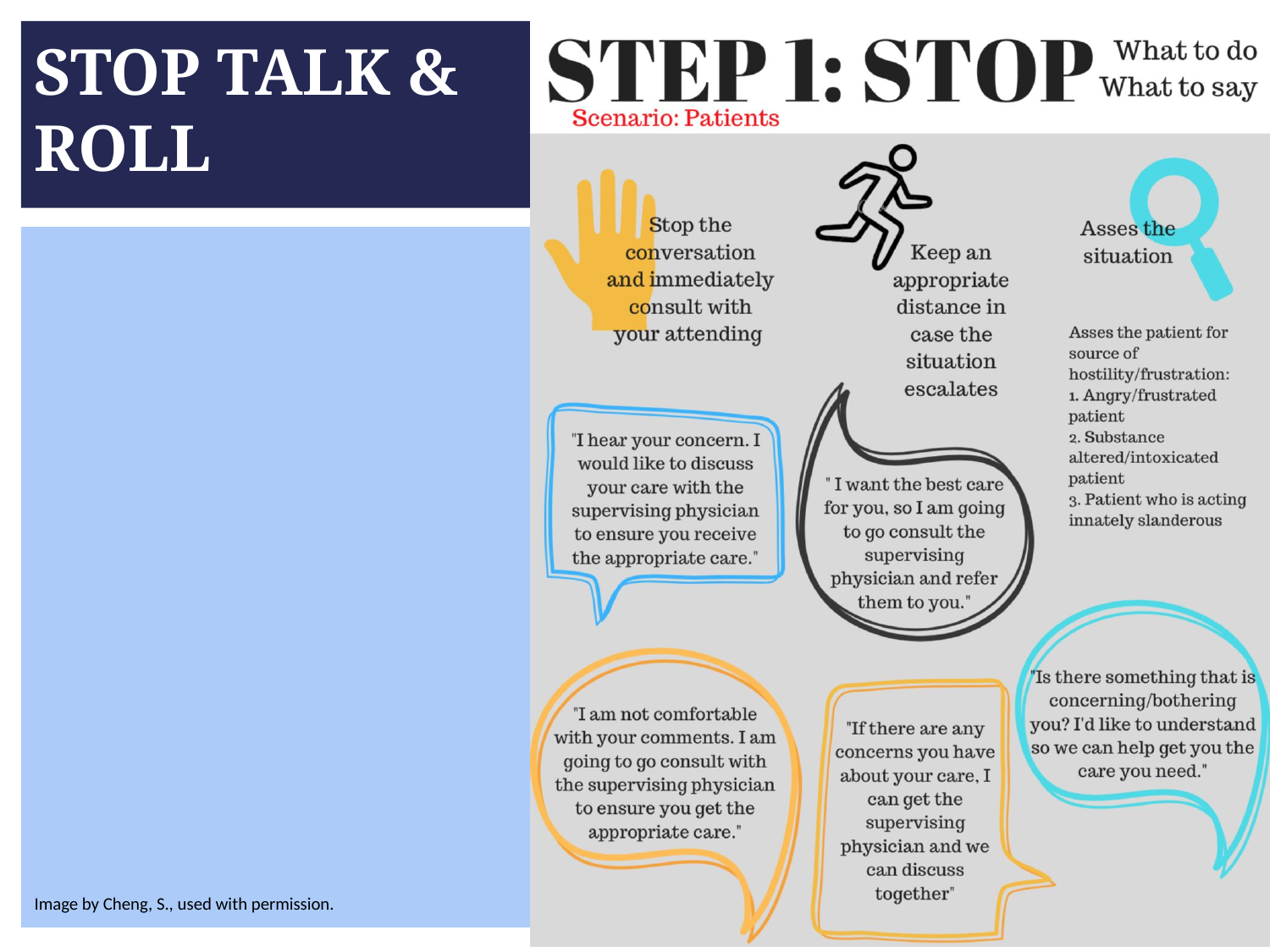

STOP TALK & ROLL
Image by Cheng, S., used with permission.

## Slide 15
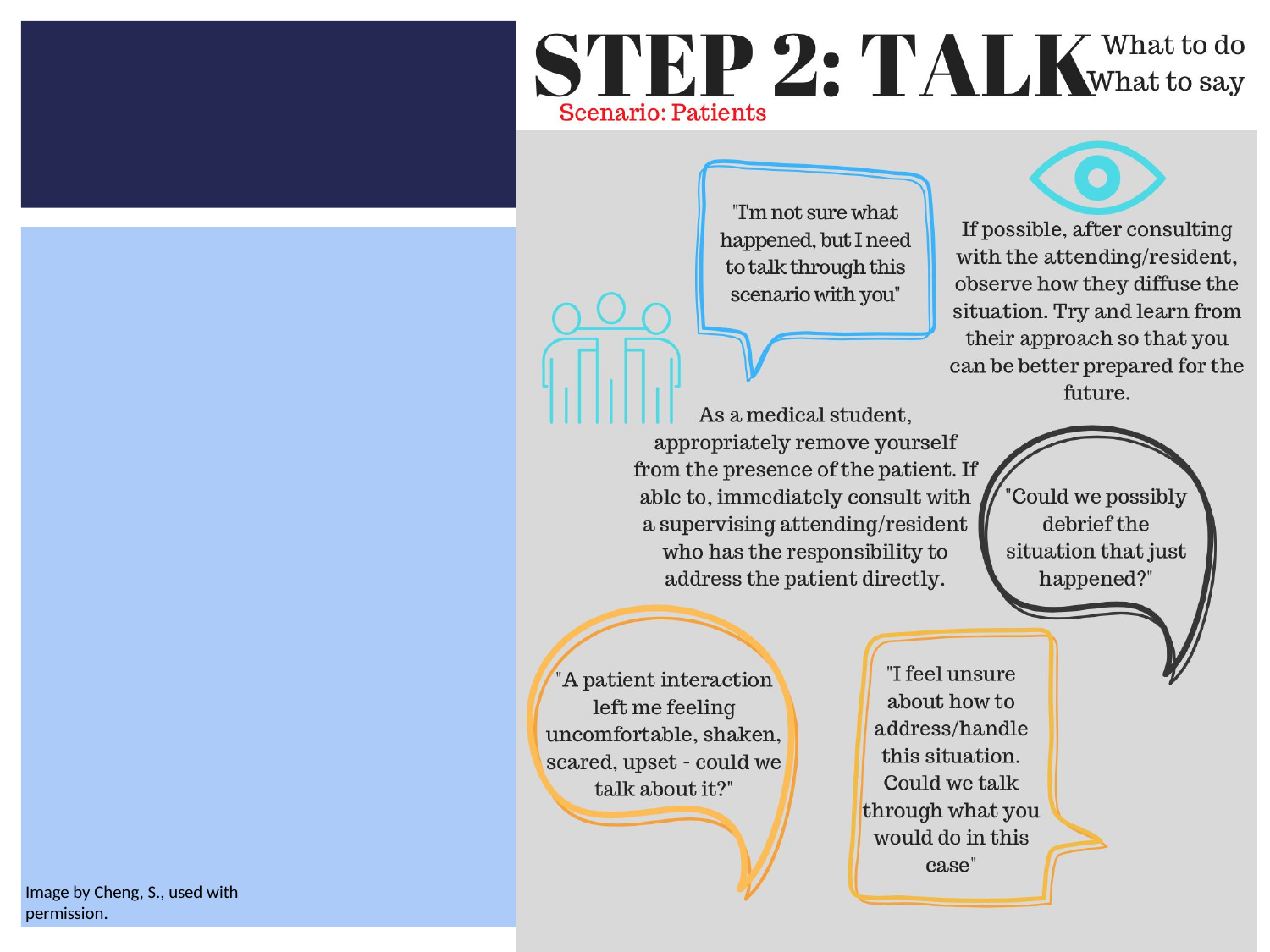

Image by Cheng, S., used with permission.

## Slide 16
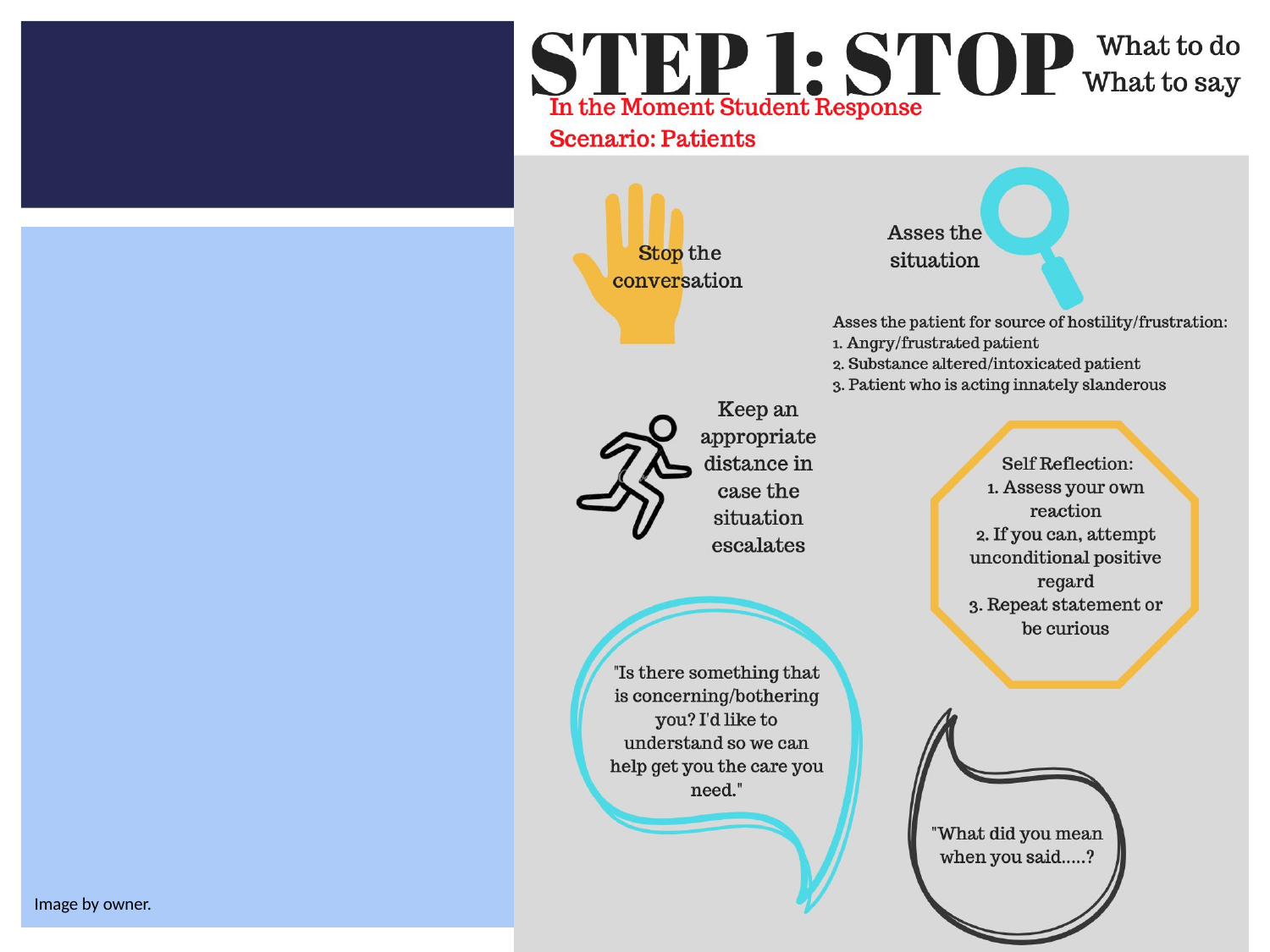

Image by owner.

## Slide 17
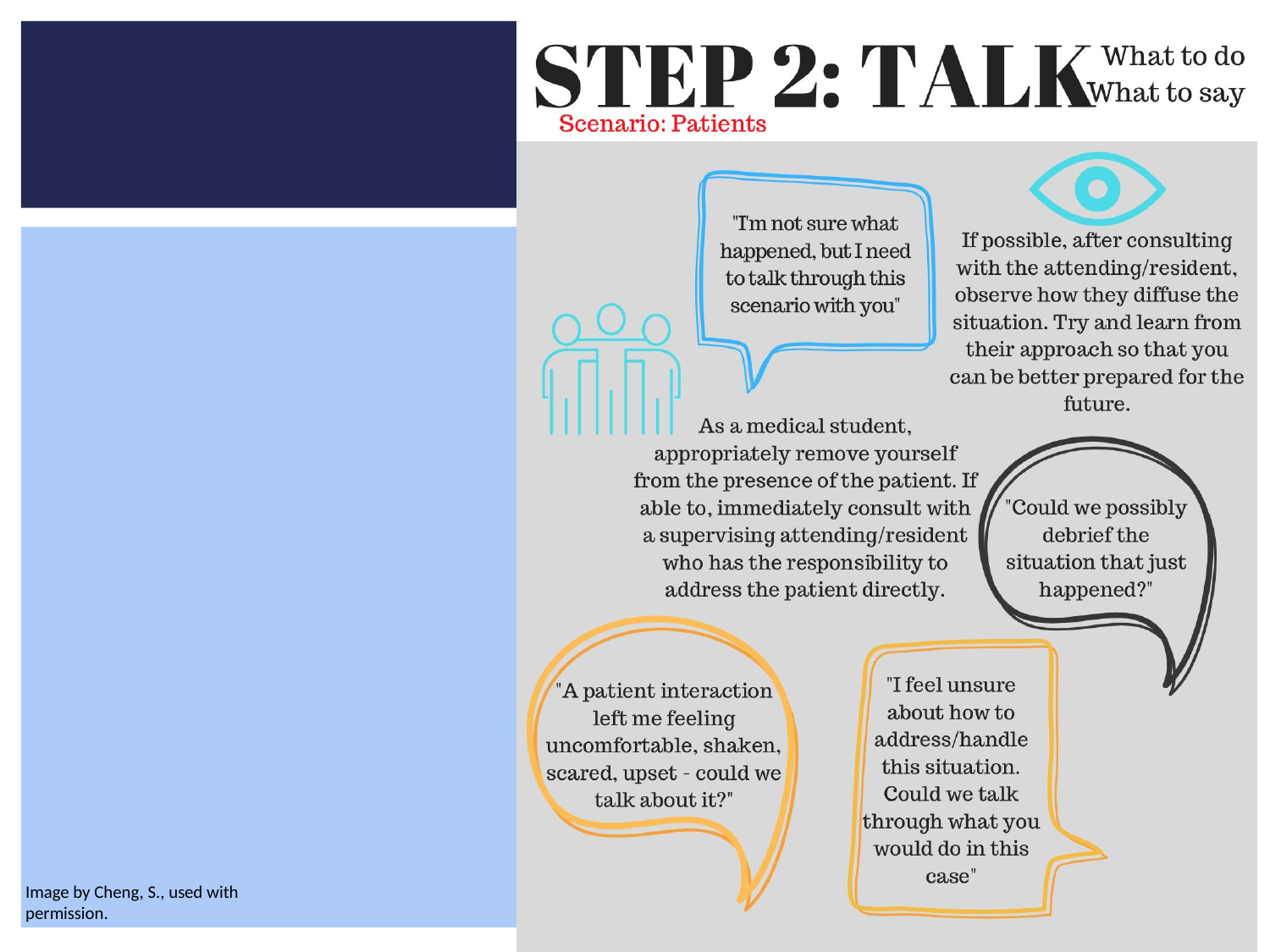

Image by Cheng, S., used with permission.

## Slide 18
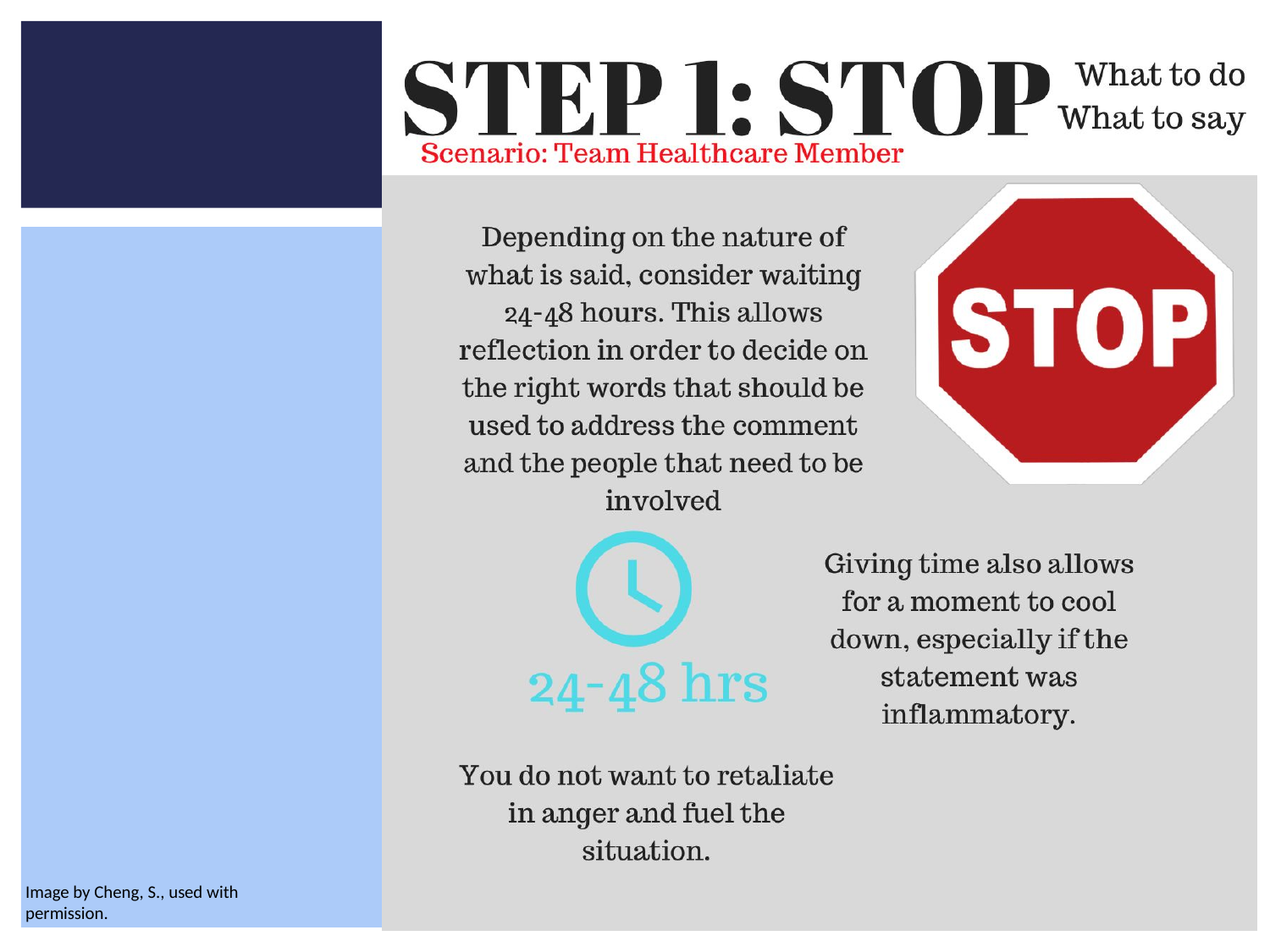

Image by Cheng, S., used with permission.

## Slide 19
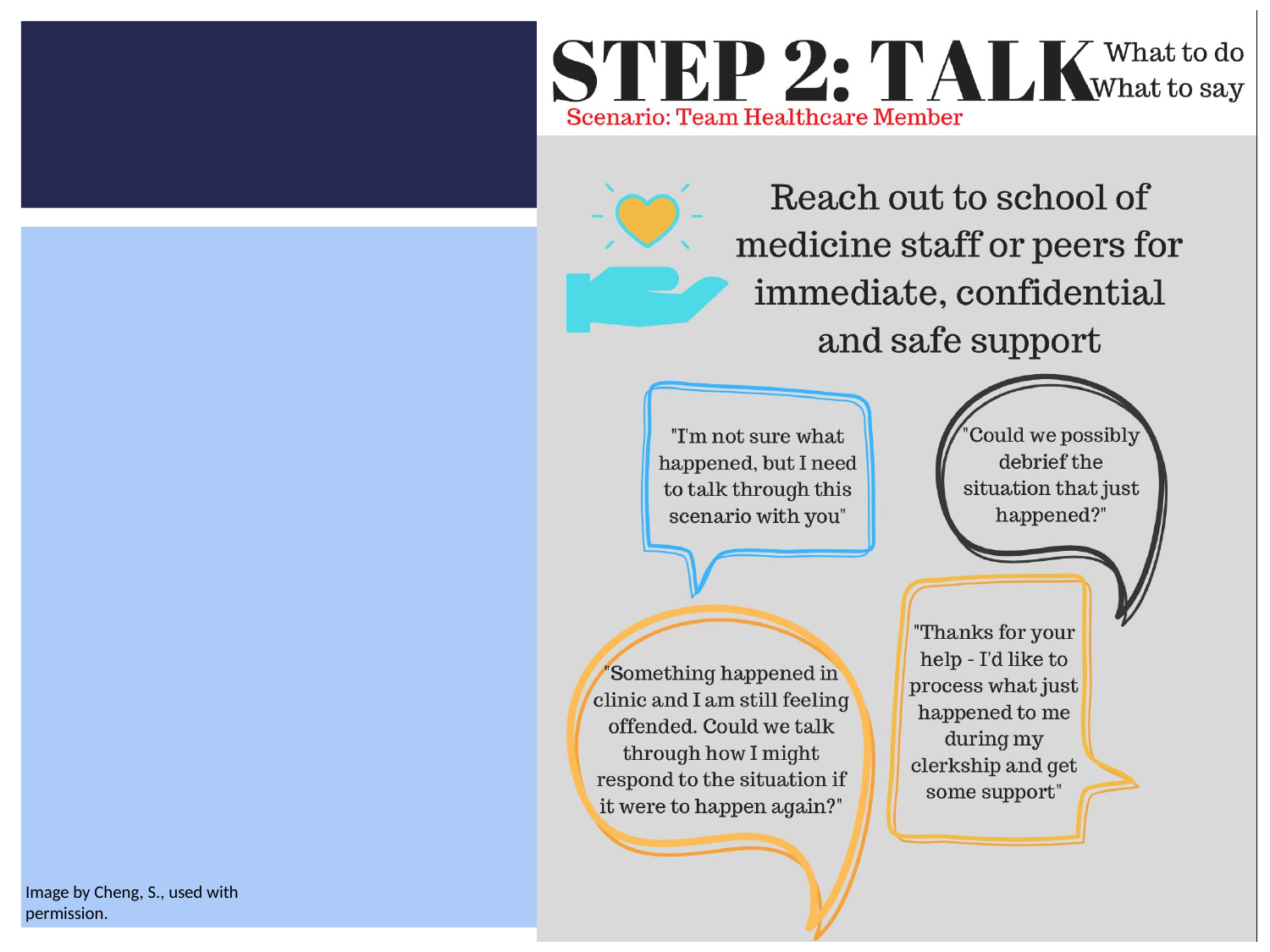

#
Image by Cheng, S., used with permission.

## Slide 20
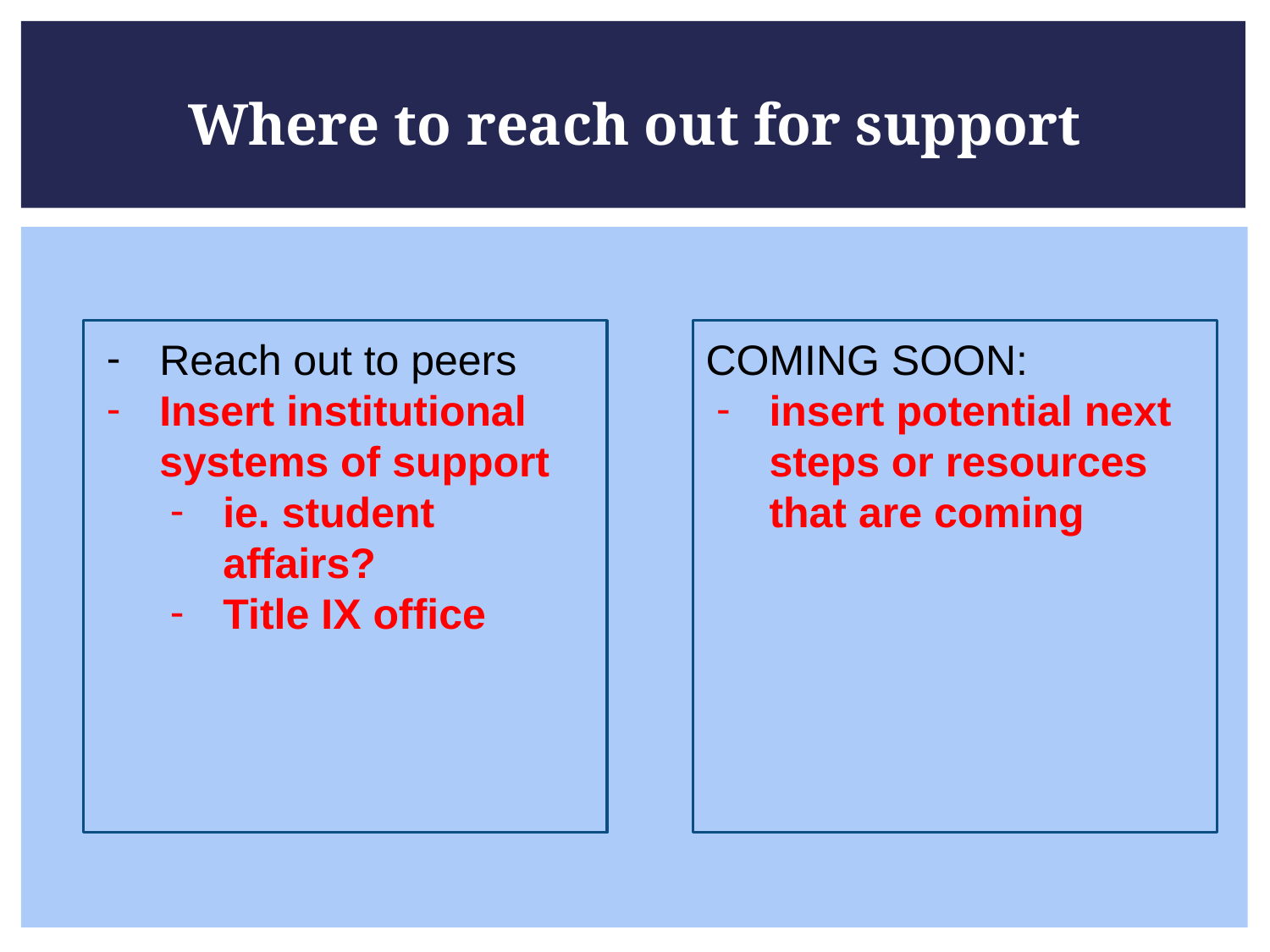

# Where to reach out for support
Reach out to peers
Insert institutional systems of support
ie. student affairs?
Title IX office
COMING SOON:
insert potential next steps or resources that are coming

## Slide 21
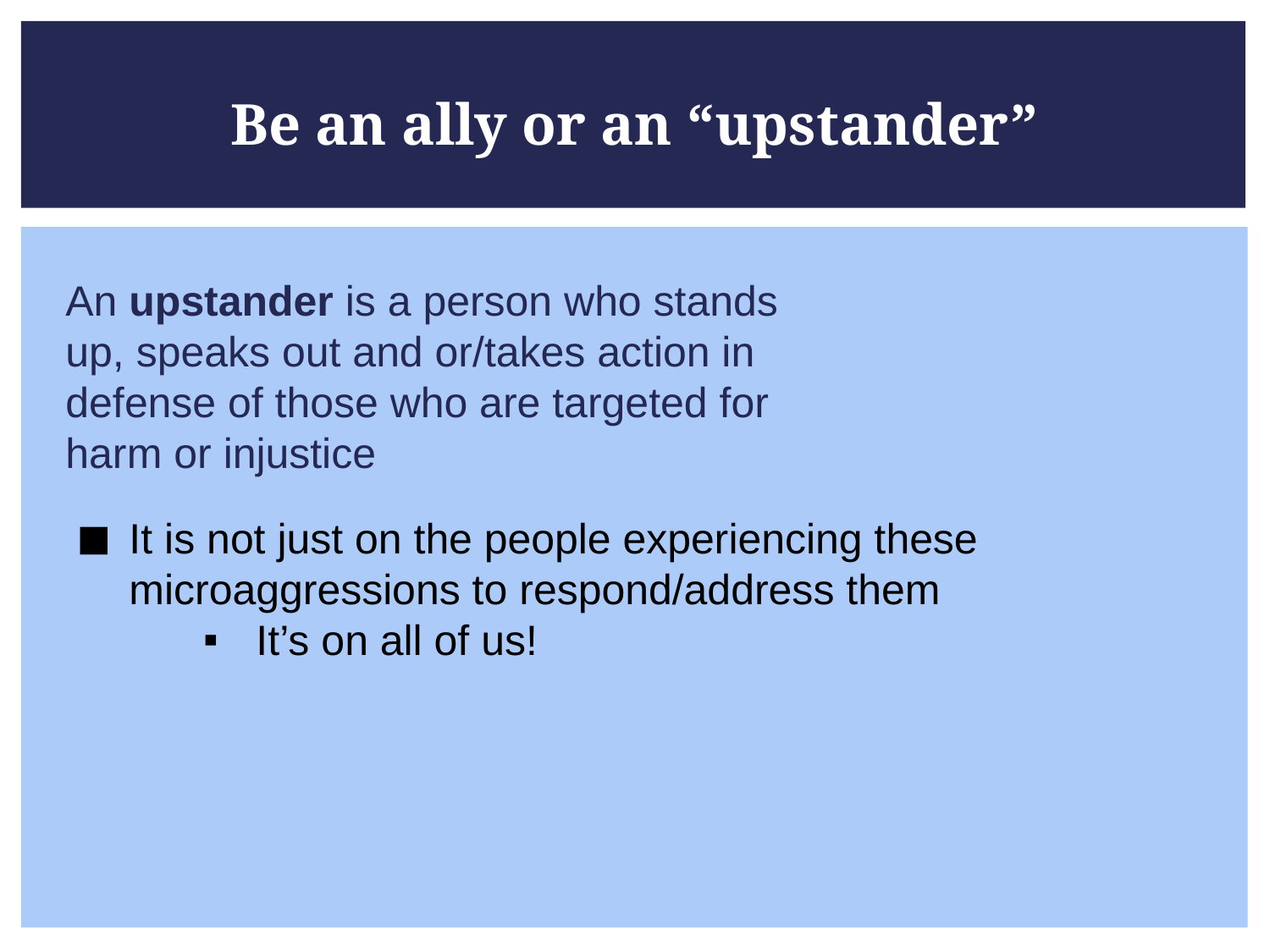

# Be an ally or an “upstander”
An upstander is a person who stands up, speaks out and or/takes action in defense of those who are targeted for harm or injustice
It is not just on the people experiencing these microaggressions to respond/address them
It’s on all of us!

## Slide 22
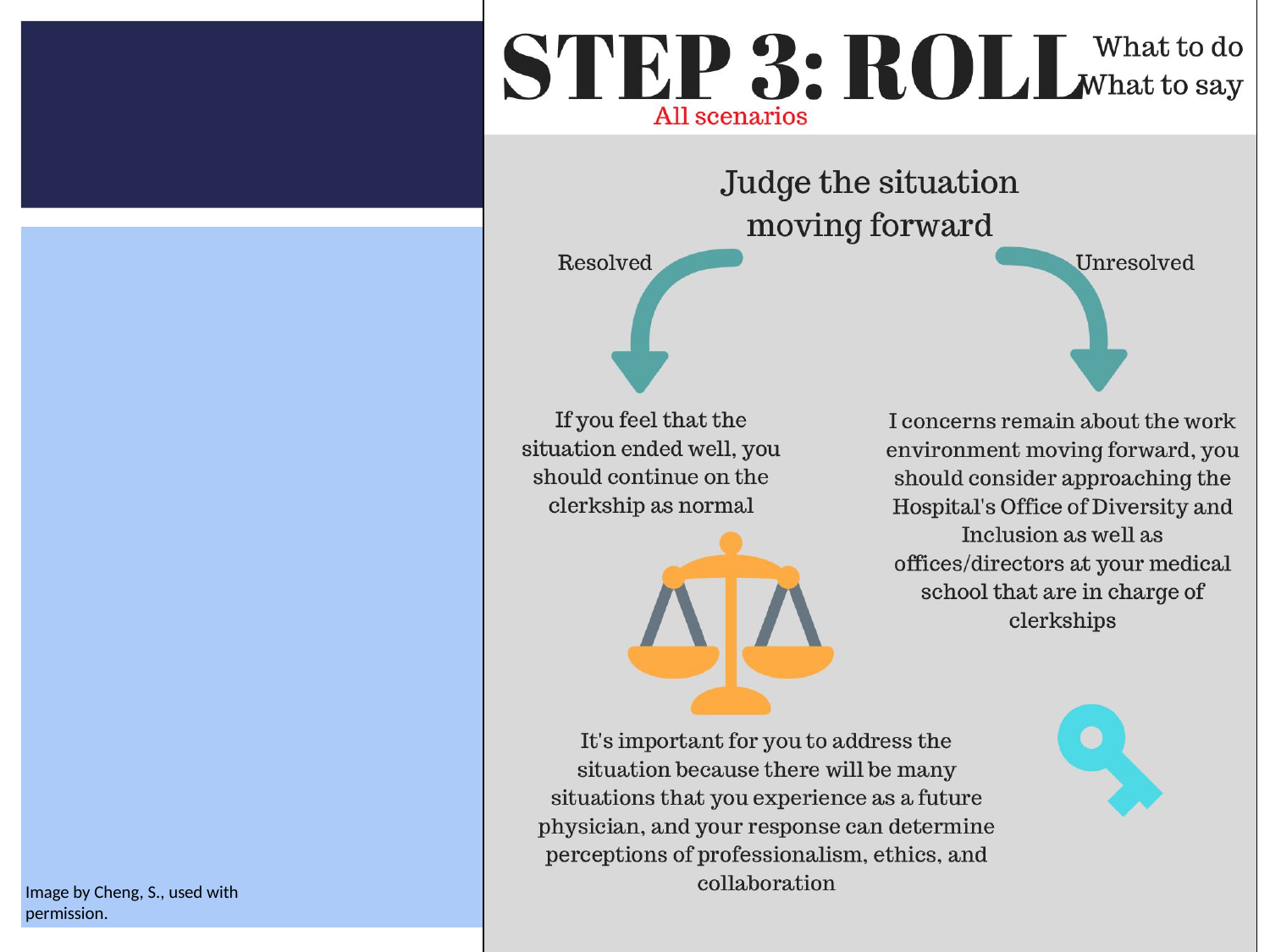

Image by Cheng, S., used with permission.

## Slide 23
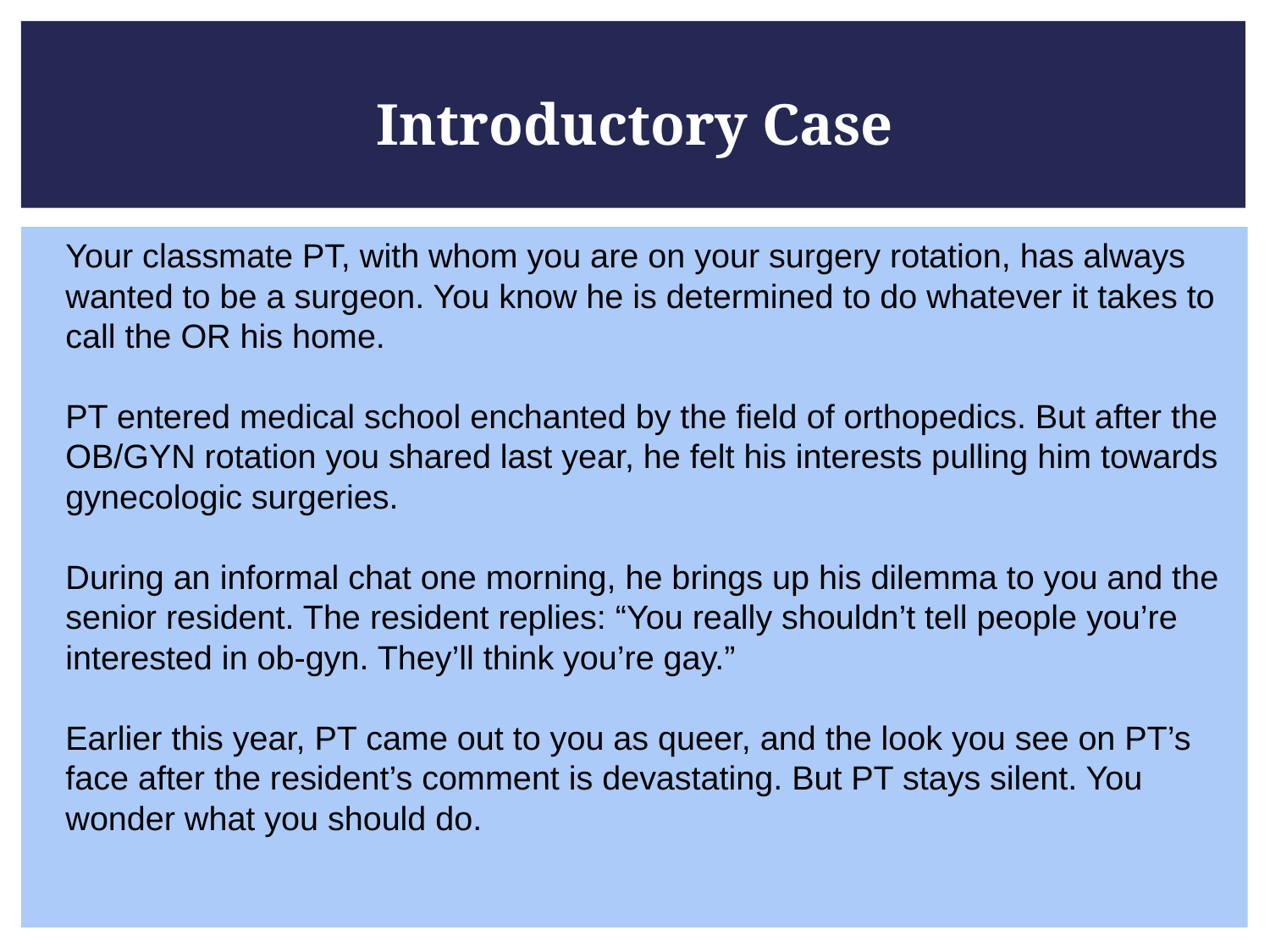

# Introductory Case
Your classmate PT, with whom you are on your surgery rotation, has always wanted to be a surgeon. You know he is determined to do whatever it takes to call the OR his home.
PT entered medical school enchanted by the field of orthopedics. But after the OB/GYN rotation you shared last year, he felt his interests pulling him towards gynecologic surgeries.
During an informal chat one morning, he brings up his dilemma to you and the senior resident. The resident replies: “You really shouldn’t tell people you’re interested in ob-gyn. They’ll think you’re gay.”
Earlier this year, PT came out to you as queer, and the look you see on PT’s face after the resident’s comment is devastating. But PT stays silent. You wonder what you should do.

## Slide 24
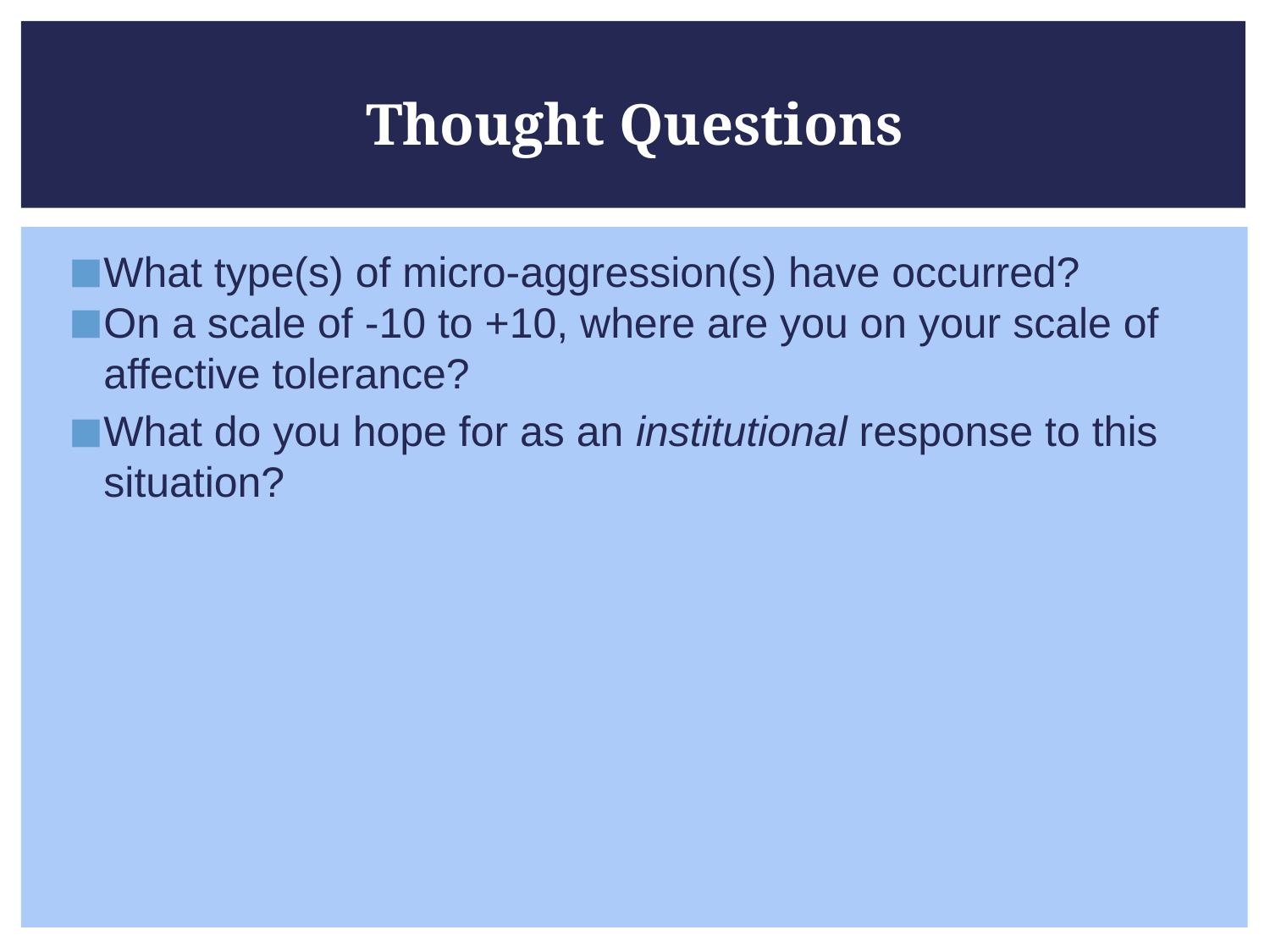

# Thought Questions
What type(s) of micro-aggression(s) have occurred?
On a scale of -10 to +10, where are you on your scale of affective tolerance?
What do you hope for as an institutional response to this situation?

## Slide 25
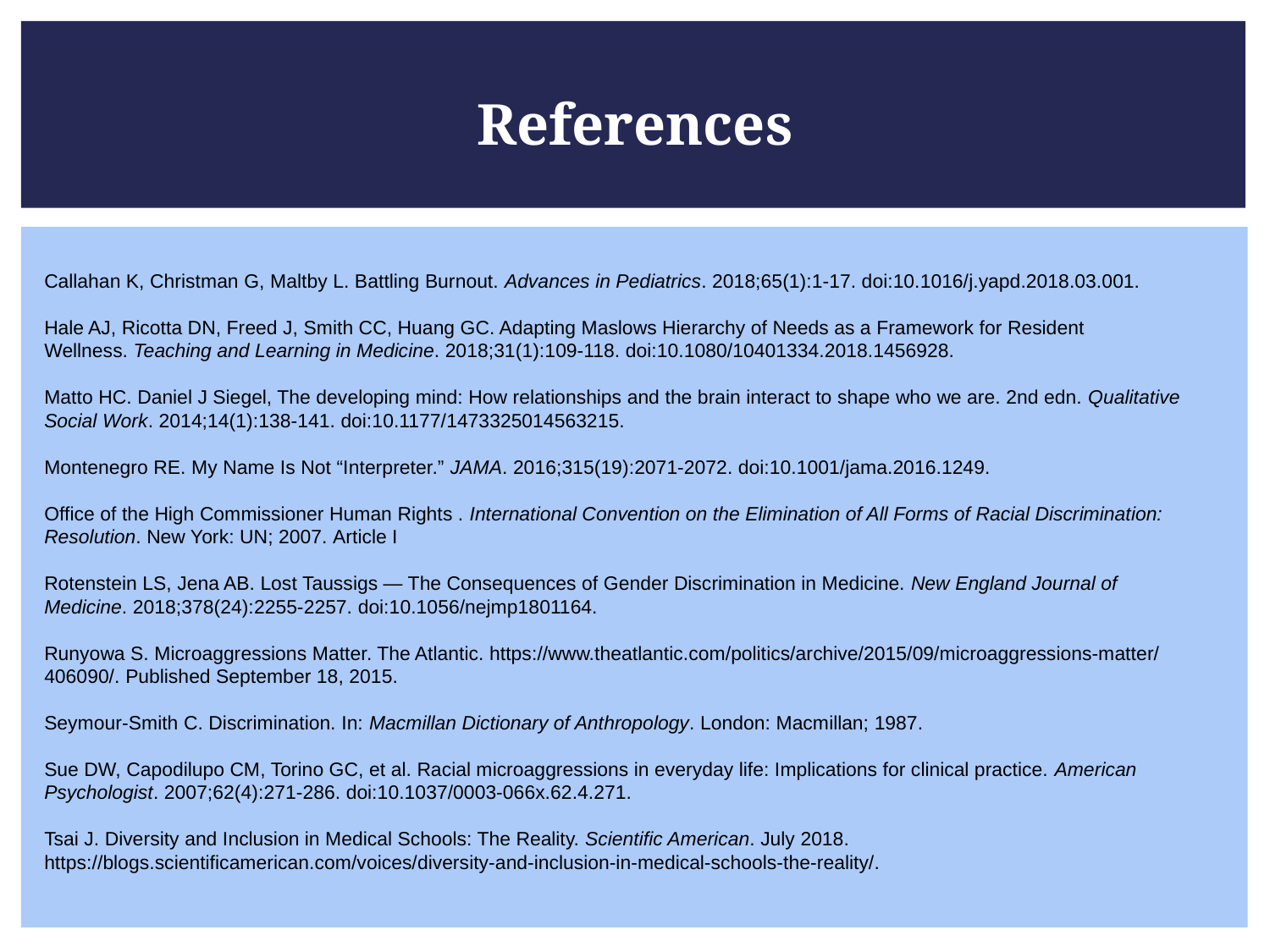

# References
Callahan K, Christman G, Maltby L. Battling Burnout. Advances in Pediatrics. 2018;65(1):1-17. doi:10.1016/j.yapd.2018.03.001.
Hale AJ, Ricotta DN, Freed J, Smith CC, Huang GC. Adapting Maslows Hierarchy of Needs as a Framework for Resident Wellness. Teaching and Learning in Medicine. 2018;31(1):109-118. doi:10.1080/10401334.2018.1456928.
Matto HC. Daniel J Siegel, The developing mind: How relationships and the brain interact to shape who we are. 2nd edn. Qualitative Social Work. 2014;14(1):138-141. doi:10.1177/1473325014563215.
Montenegro RE. My Name Is Not “Interpreter.” JAMA. 2016;315(19):2071-2072. doi:10.1001/jama.2016.1249.
Office of the High Commissioner Human Rights . International Convention on the Elimination of All Forms of Racial Discrimination: Resolution. New York: UN; 2007. Article I
Rotenstein LS, Jena AB. Lost Taussigs — The Consequences of Gender Discrimination in Medicine. New England Journal of Medicine. 2018;378(24):2255-2257. doi:10.1056/nejmp1801164.
Runyowa S. Microaggressions Matter. The Atlantic. https://www.theatlantic.com/politics/archive/2015/09/microaggressions-matter/406090/. Published September 18, 2015.
Seymour-Smith C. Discrimination. In: Macmillan Dictionary of Anthropology. London: Macmillan; 1987.
Sue DW, Capodilupo CM, Torino GC, et al. Racial microaggressions in everyday life: Implications for clinical practice. American Psychologist. 2007;62(4):271-286. doi:10.1037/0003-066x.62.4.271.
Tsai J. Diversity and Inclusion in Medical Schools: The Reality. Scientific American. July 2018. https://blogs.scientificamerican.com/voices/diversity-and-inclusion-in-medical-schools-the-reality/.
