# Supplementary material for: Building a Tool Kit for Medical and Dental Students: Addressing Microaggressions and Discrimination on the Wards
Source: MedEdPORTAL. 2020 Apr 3;16:10893. doi: 10.15766/mep_2374-8265.10893 (PMC7187912; doi:10.15766/mep_2374-8265.10893)
Supplement: Supplementary file 1 — PowerPoint Presentation.pptxCases.docxRole Cards.docxFramework Handout.docxFacilitator Guide.docxAbridged Facilitator Guide.docxPreworkshop Survey.docxPostworkshop Survey.docxText Exercise Criteria.docx [file mep-16-10893-s001.zip › B. Cases.docx]

**CASE 1:**

A 82 year-old male, Mr. RS, is admitted from the ED to general medical services with change in mental status. KP is an Asian-American 4th year on her medicine sub-I rotation and picks up Mr. RS on call. On morning rounds, she presents that his change in mental status was likely due to delirium. Upon entering the room, the team finds a somnolent Mr. RS dozing off while having his breakfast. He becomes more alert and looks around the room at the team: a male attending, a male senior resident, a male intern, and two female medical students (KP and a second-year student ST).

The attending asks Mr. RS how he is doing, but his response is difficult to apprehend. When asked to repeat himself, he points to ST, a female Black student, and states, loudly “I do not want her here!” The attending then asks ST to check on the next patient, and she leaves the room.

Changing the subject, the resident asks the patient if the team can examine him. “Of course! Where’s my favorite nurse?” he states with a smile, motioning to KP. While assessing Mr. RS’s pupillary reaction, the attending asks him to focus on a spot across the room. He makes eye contact with KP and asserts slyly, “I can focus on her all day! Where are you from anyway?”

The team concludes the visit and leaves the room, where they reconnect with ST, who has just visited another patient. While discussing the medical plan for Mr. RS, the attending states to the team he feels they, “handled the interaction professionally.”

**CASE 2:**

PS, a Latinx 2nd year medical student, is on his first day of surgical rounds. The team--the attending, a resident, and two medical students (PS and AB, a white female 2nd year)--is moving through rooms quickly in order to get to the operating room. Their next patient is GG, a Spanish speaking woman status post hernia repair, whose 15 year old son is in the room with her. Neither the attending nor residents speak Spanish. Although they have called and are waiting for the interpreter, they are impatient to continue rounds. Finally the attending physician says to the boy, “Son, could you just ask your mom how she’s doing? How the surgical site is healing? Is she passing gas?”

Both mother and son look concerned. PS, who speaks Spanish steps in and says “no se preocupe - don’t worry” and reassures the patient that they are getting the care they need to be ok. The attending says, “PS, could you translate for us?” PS grew up speaking Spanish at home but doesn’t know a lot of medical terminology. Still, he says “yes” in order to be helpful. At that moment, the interpreter enters, and the visit continues.

Later that day, the resident asks PS if he will translate the discharge documents into Spanish for GG before the next morning--”The translating service takes a long time and we want to get her discharged first thing. I know you’ll do a good job.”

PS spends over an hour that evening meticulously translating the documents that night, which cuts into his study time, but PS is eager to be helpful to the team and the family. He presents the documents to the resident the next morning. The resident doesn’t look up from the computer screen, saying, “Great! Just give them to the nurse.” During team rounds, the attending asks PS some pointed questions about management of post-surgical complications, one of which PS doesn’t know. “You need to spend time learning about your patients in order to take care of them,” the attending says to the team, which PS finds frustrating but acknowledges.

As the team is moving between rooms, the resident turns to the AB and says, “Wow, PS always seems unhappy. He’s not much of a team player, is he?
