# Supplementary material for: Building a Tool Kit for Medical and Dental Students: Addressing Microaggressions and Discrimination on the Wards
Source: MedEdPORTAL. 2020 Apr 3;16:10893. doi: 10.15766/mep_2374-8265.10893 (PMC7187912; doi:10.15766/mep_2374-8265.10893)
Supplement: Supplementary file 1 — PowerPoint Presentation.pptxCases.docxRole Cards.docxFramework Handout.docxFacilitator Guide.docxAbridged Facilitator Guide.docxPreworkshop Survey.docxPostworkshop Survey.docxText Exercise Criteria.docx [file mep-16-10893-s001.zip › C. Role Cards.docx]

**This is the format for the role cards for the small group portion**

**CASE 1:**

One side of the card should have one of the following:

- KP, Asian American 4th year medical student, female
- ST, Black 2nd year medical student, female
- Bystander, 2nd year medical student
- Attending

The other side of each card should have:

Instructions for analyzing cases

1. **Underline the examples of microaggression and discrimination**
2. **How do the microaggressions and discriminations in the case affect team dynamics and patient care?**

**__________________________________________________________________________________________________________________________________________________________**

**____________________________________________________________________________**

**____________________________________________________________________________**

**____________________________________________________________________________**

**___________________________________________________________________________**

**___________________________________________________________________________**

**___________________________________________________________________________**

**____________________________________________________________________________**

**____________________________________________________________________________**

**_____________________________________________________________________________**

**_____________________________________________________________________________**

**_____________________________________________________________________________**

1. **Apply/practice the frameworks for each case**
   1. **Students will role play by assuming the roles given on the notecards and as they do will consider the following questions:**
      1. What actions/words could you say in this scenario?
      2. What is the intended outcome of that?
      3. What are some limitations to doing that?

**CASE 2:**

One side of the card should have one of the following:

- PS, Latinx 2nd year medical student, male
- AB, White 2nd year medical student, female
- Bystander, 2nd year medical student
- Attending/Resident

The other side of each card should have:

Instructions for analyzing cases

1. **Underline the examples of microaggression and discrimination**
2. **How do the microaggressions and discriminations in the case affect team dynamics and patient care?**

**__________________________________________________________________________________________________________________________________________________________**

**____________________________________________________________________________**

**____________________________________________________________________________**

**____________________________________________________________________________**

**___________________________________________________________________________**

**___________________________________________________________________________**

**___________________________________________________________________________**

**____________________________________________________________________________**

**____________________________________________________________________________**

**_____________________________________________________________________________**

**_____________________________________________________________________________**

**_____________________________________________________________________________**

1. **Apply/practice the frameworks for each case**
   1. **Students will role play by assuming the roles given on the notecards and as they do will consider the following questions:**
      1. What actions/words could you say in this scenario?
      2. What is the intended outcome of that?
      3. What are some limitations to doing that?
