# Supplementary material for: Building a Tool Kit for Medical and Dental Students: Addressing Microaggressions and Discrimination on the Wards
Source: MedEdPORTAL. 2020 Apr 3;16:10893. doi: 10.15766/mep_2374-8265.10893 (PMC7187912; doi:10.15766/mep_2374-8265.10893)
Supplement: Supplementary file 1 — PowerPoint Presentation.pptxCases.docxRole Cards.docxFramework Handout.docxFacilitator Guide.docxAbridged Facilitator Guide.docxPreworkshop Survey.docxPostworkshop Survey.docxText Exercise Criteria.docx [file mep-16-10893-s001.zip › D. Framework Handout.docx]

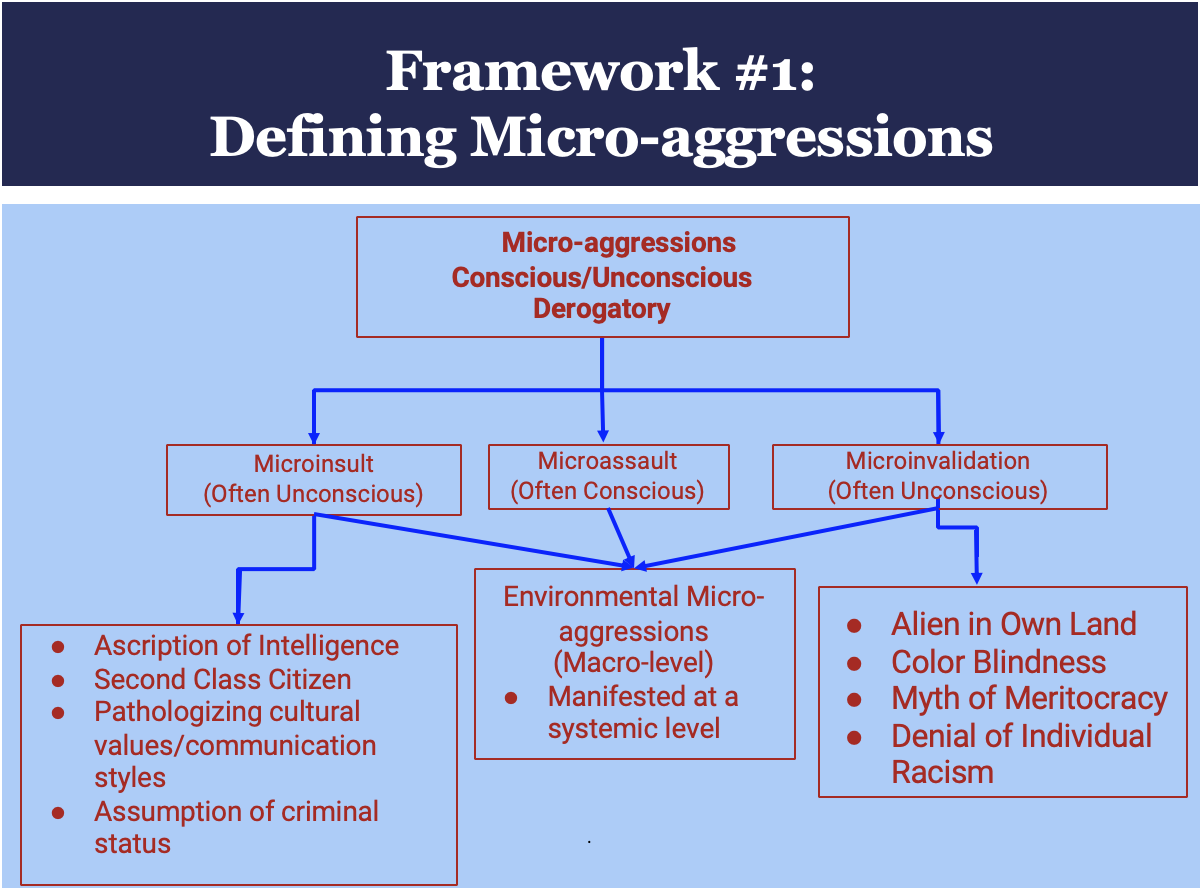


Sue DW, Capodilupo CM, Torino GC, et al. Racial microaggressions in everyday life: Implications for clinical practice. *American Psychologist*. 2007;62(4):271-286. doi:10.1037/0003-066x.62.4.271.


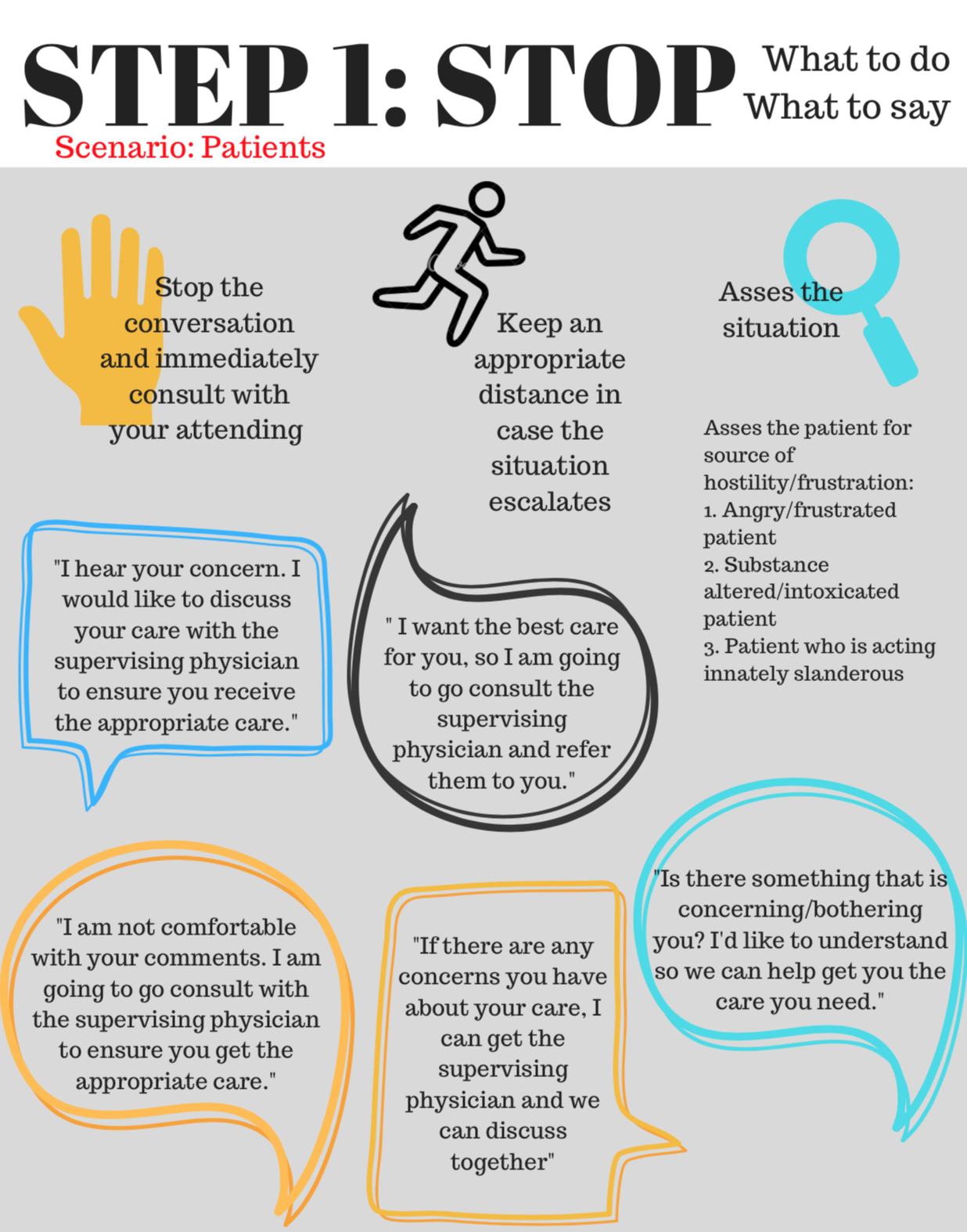
 Image by Cheng, S., used with permission


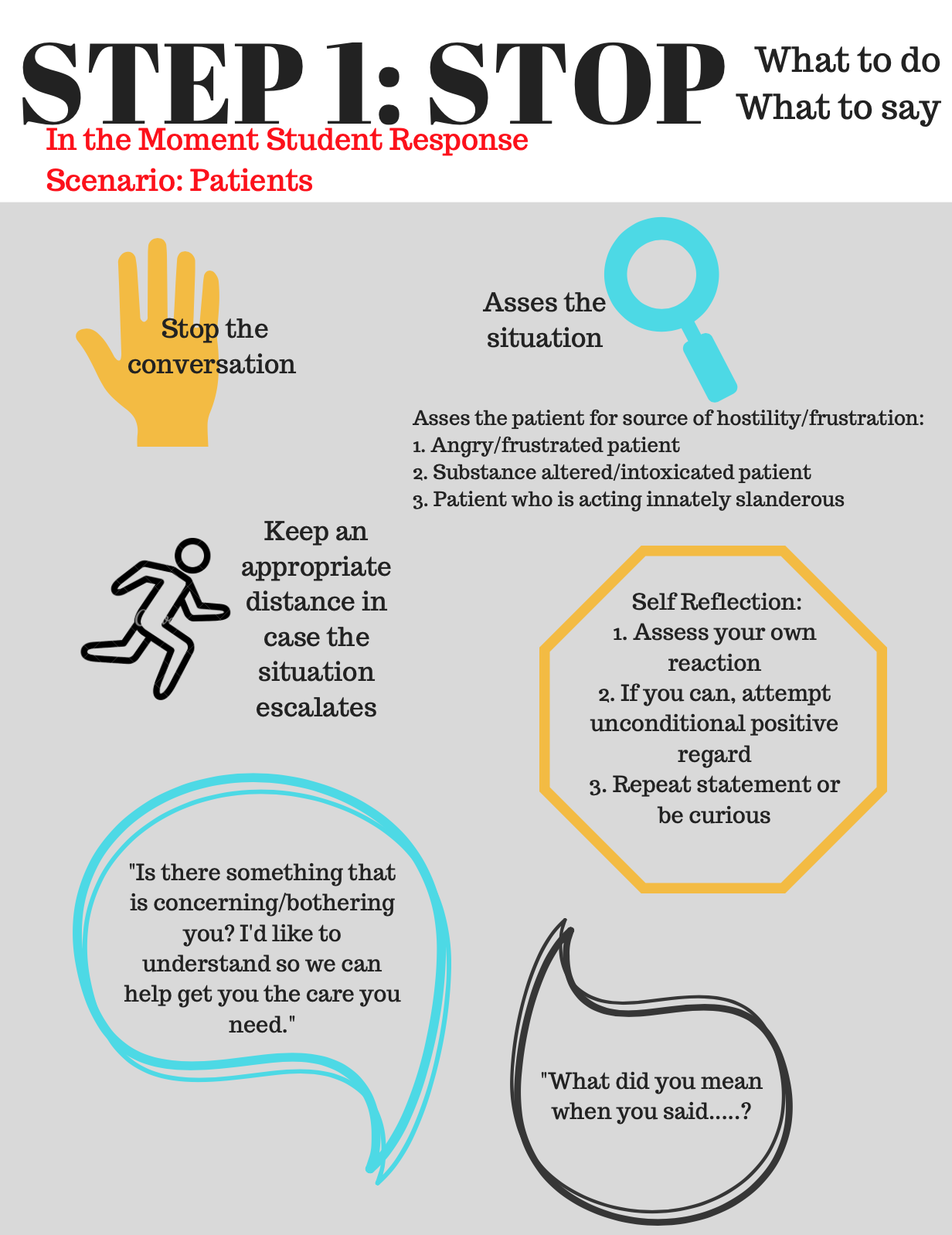


Author owned.


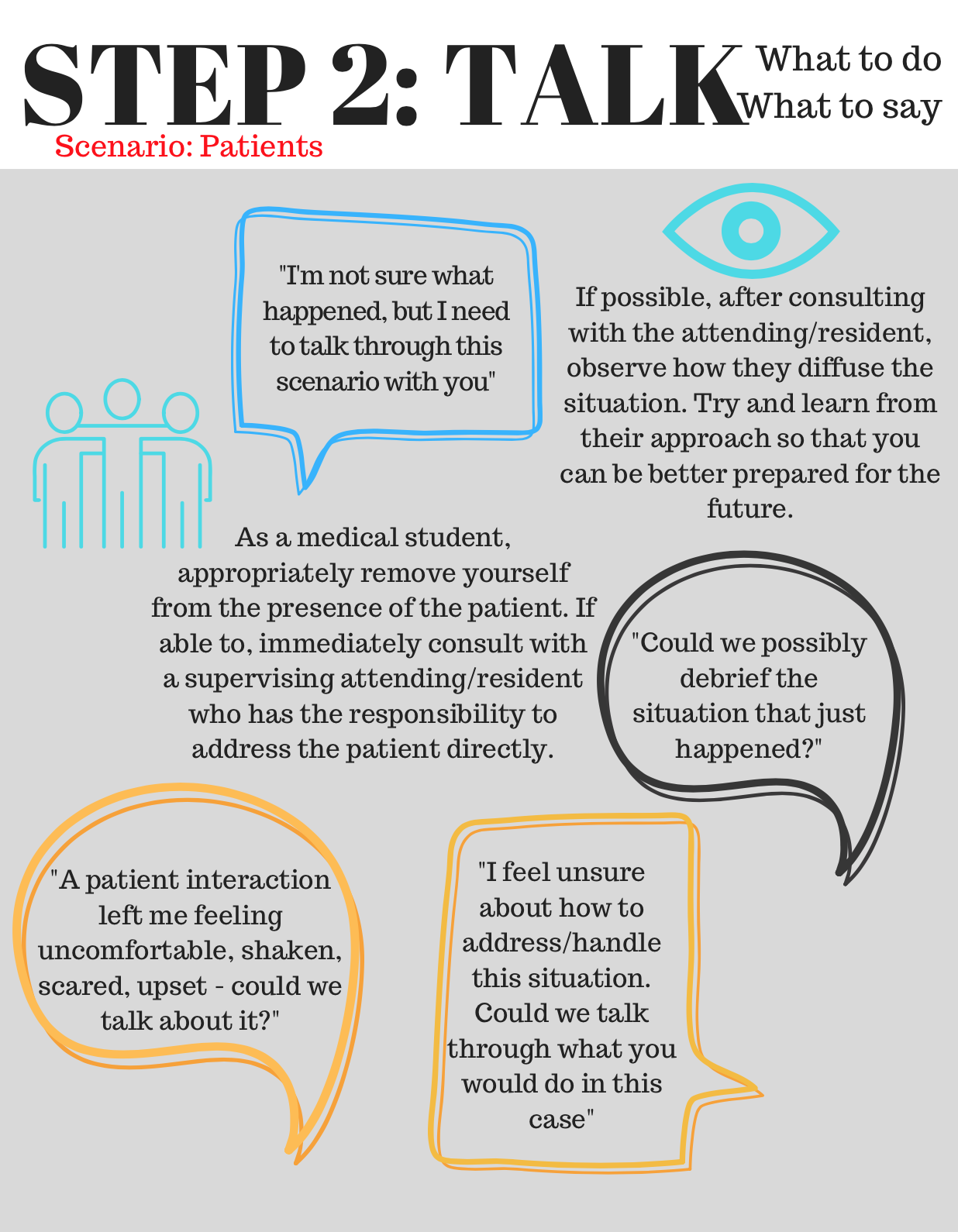
 Image by Cheng, S., used with permission


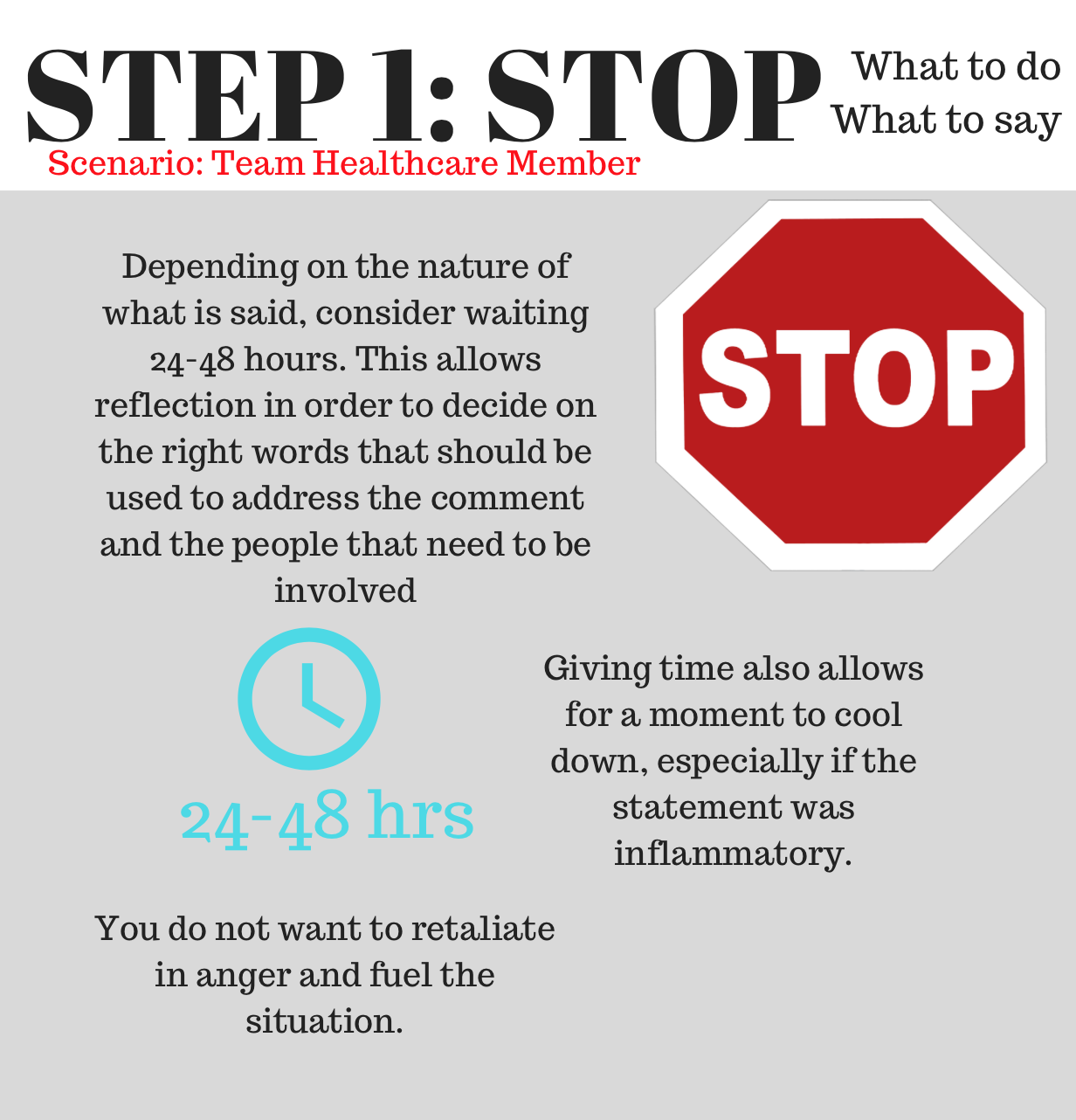
 Image by Cheng, S., used with permission


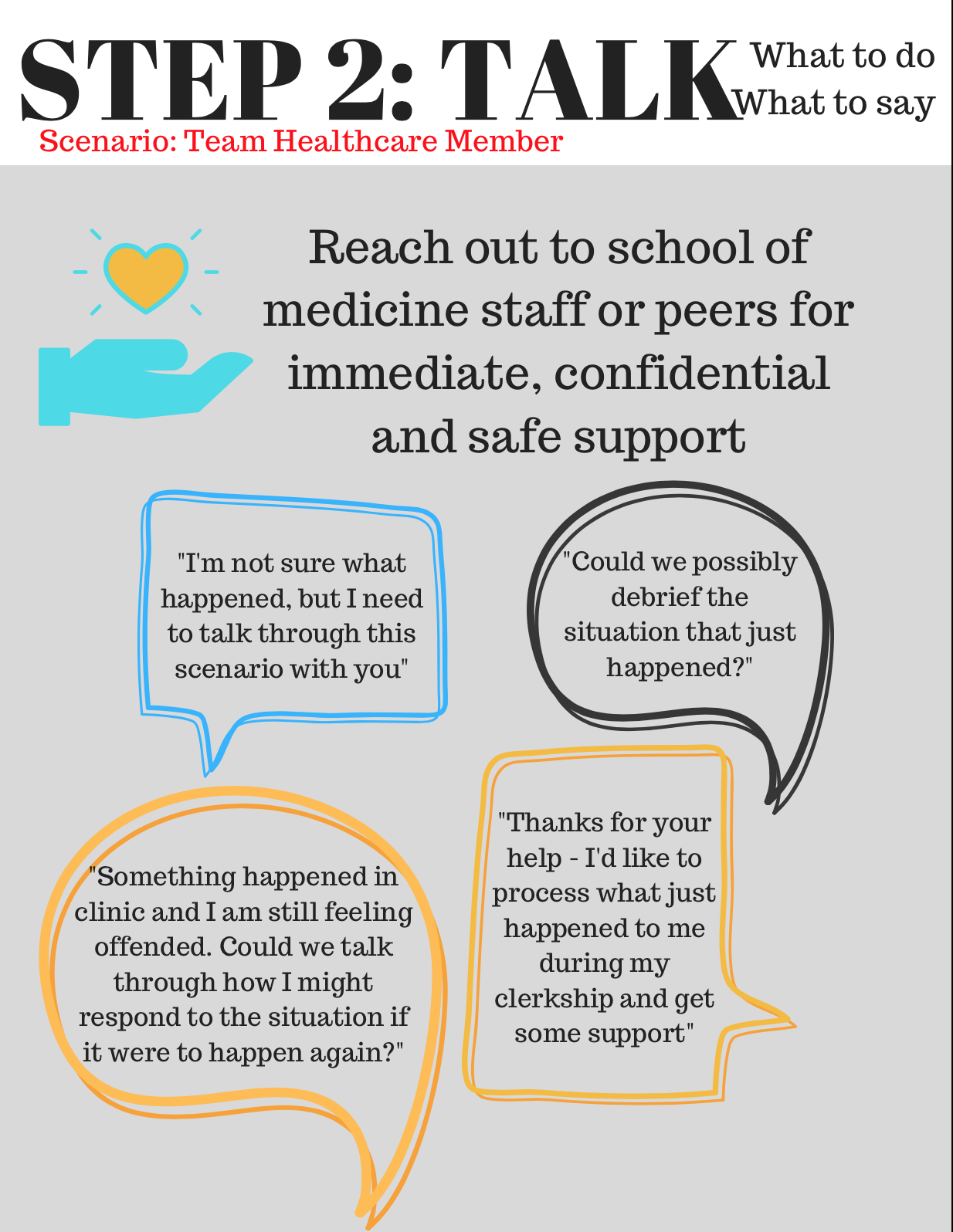
 Image by Cheng, S., used with permission


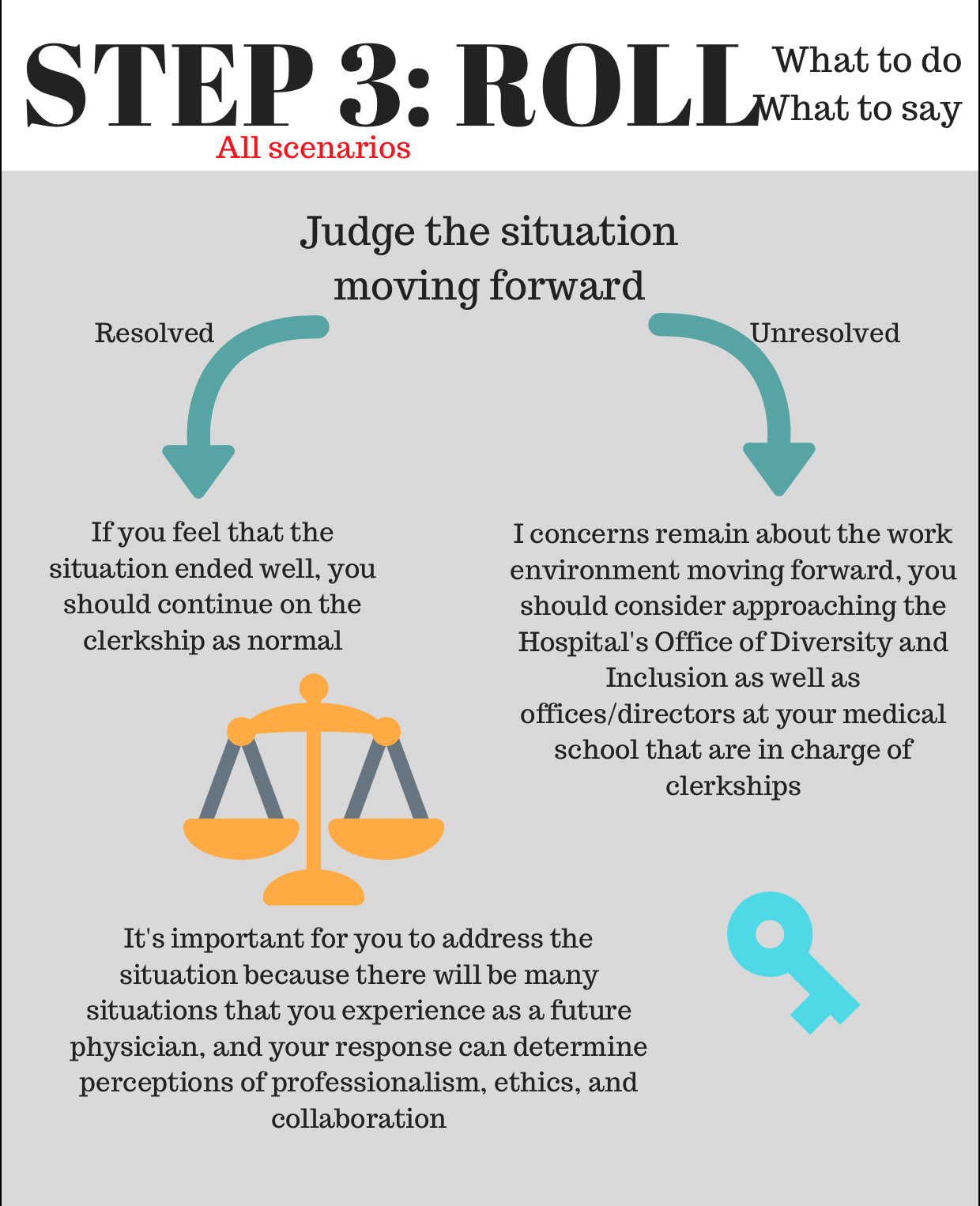
 Image by Cheng, S., used with permission
