# Supplementary material for: Building a Tool Kit for Medical and Dental Students: Addressing Microaggressions and Discrimination on the Wards
Source: MedEdPORTAL. 2020 Apr 3;16:10893. doi: 10.15766/mep_2374-8265.10893 (PMC7187912; doi:10.15766/mep_2374-8265.10893)
Supplement: Supplementary file 1 — PowerPoint Presentation.pptxCases.docxRole Cards.docxFramework Handout.docxFacilitator Guide.docxAbridged Facilitator Guide.docxPreworkshop Survey.docxPostworkshop Survey.docxText Exercise Criteria.docx [file mep-16-10893-s001.zip › E. Facilitator Guide.docx]

### **Session Guide for Small-Group Facilitators:**

### **Building a Toolkit:**

### **Addressing Microaggressions and Discrimination on the Wards**

When the workshop is happening

***when you need facilitators to check-in**

Where the workshop is happening

**Schedule:** **Schedule based on a 2hr session**

10am-10:30am: Didactic session

10:30am-10:45am: Transition to small groups

10:45am-10:55am: Introductions, ground rules

10:55am-11:25am: Case 1

11:25am-11:55am: Case 2

11:55am-12pm: Closing and post-workshop survey

**Objectives:**

Define and recognize a microaggression.

1. Identify and describe the impact of microaggressions on clinical care
2. Identify and explain challenges to confronting microaggressions in a clinical setting
3. Apply the Georgetown framework to clinical scenarios involving bias on the wards
4. Envision systems-level changes that might best support students in addressing bias on the wards.

**Group norms:**

**1. Be 100% present.** The success and value of this session depends on the full commitment and participation of every single student in the group. We expect everyone to be fully mentally and physically present. Please do not use any electronic devices during the discussion.
**2. Respect where everyone lives on the window of affective tolerance.** We’ll be discussing many sensitive issues, many of which are directly based on the lived experiences of your classmates. These issues will be deeply personal to some people. Please respect the emotions of everyone in the room - all emotions are valid.
**3. Demonstrate strength by being vulnerable.** Speak your truth. Do not be afraid to ask questions, push back, or share personal stories. This is how we will all grow.

**4. Keep everything confidential, Vegas style.** This is a safe space in which we would like everyone to feel comfortable expressing themselves. What’s discussed in the group stays in the group!
5. **Be conscious of power dynamics.** We are all entering this space with different levels of prior knowledge. Just as we do in the learning studios, please give others space to think and reflect before sharing your views. (Remember to W.A.I.T. - why am I talking? Or, why *aren’t* I talking?) Be aware of how you use your privilege, from taking up too much emotional space to disengaging.

**Frameworks:**

1. **Definition of Microaggressions Sue et al American Psychological Association Vol. 62, No. 4, 271–282, 2007.**

The aim of this framework is to develop a taxonomy for talking about microaggressions/discriminatory behavior and macro-aggressions. It also provides a language to explain why the more subtle types of microaggressions can also be extremely damaging. Three sub-groups

1. **Micro-assault:** These are often **conscious** and **explicit** and they are meant to hurt the victim through name calling, racial slurs, avoidant behavior or purposeful discriminatory actions. It is similar to one one would think of as blatant discrimination or a macro-aggression, e.g. referring to someone with racial epithets, using the comment “that’s so gay” to connote something bad or wierd. Others include describing someone as “Oriental” or displaying a swastika
2. **Microinsult:** These are often **unconscious** and can be verbal or non-verbal. They are behavioral or verbal remarks or comments that convey rudeness, insensitivity and demean a person’s heritage or identity. E.g. assigning a degree of intelligence to a person of color based on their race. People of Color being asked by others “How did you get your job?” or being told “You are so articulate.” Another is assuming that all Blacks are criminals or “All Asians are good in Science and Math”. Another example is commenting that a woman is “too pretty to be a lesbian.”
3. **Microinvalidation:** These are often **unconscious** are characterized by communications that exclude, negate or nullify the psychological thoughts, feelings or experiential reality of minorities e.g. people of color, LGBTQ etc.

For example when LGBTQ people are told that their perceptions of discrimination are unfounded or nonsensical negating the realities of heterosexism or transphobia in their lives. Another example are statements which assert that race plays a minor role in life’s success or denial of personal racism or one’s role in this perpetration. “Everyone can be successful if they work hard.”

1. **Response to Microaggressions**

There are at least four psychological dilemmas that arise from experiences with micro-aggressions.

1. **Clash of realities:** perpetrator thinks they are harmless while target thinks its a reflection of bias. e.g. use of incorrect prononous with gender nonconforming (GNC) people. GNC person is aggravated that this continually occurs but cisgender people who commit these view their behaviors as honest mistakes which are common or even accurate.
2. **Invisibility of unintentional bias:** people are socialized to learn biases due to systemic oppression and the superiority of dominant groups e.g. cisgender people may not realize there are no gender-neutral restrooms in certain public spaces because they have been socialized to believe that there are only two kinds of restrooms, for men and for women, are needed.
3. **Perceived minimal harm of microaggressions:** many people, particularly of privileged groups may view microaggressions as being unimportant or unworthy of discussion because the specific incidents are innocuous and minor but when they occur often it leads to an accumulation which can negatively affect mental health and wellbeing.
4. **Catch 22**- exemplifies why it is often difficult to respond to microaggressions because
   1. Not all individuals view the incident the same
   2. There usually are repercussions for confronting the perpetrator
   3. The target may not have the energy, time or mental energy to engage in such conversations

To counteract this difficulty with responding to microaggression we present a framework of how to respond to microaggressions based off of a [framework developed by Georgetown University School of Medicine](https://som.georgetown.edu/diversityandinclusion/stoptalkroll). The framework has 3 basic components: STOP, TALK, ROLL.

- STOP: Stop refers to hitting pause on the interaction and assessing the situation. This could mean deciding whether or not to address it right then and there, or realizing need time to process and decide what actions to take next.
- TALK: Talk refers to what could possibly be said in the context of the scenario right then and there, whether it is addressing what just happened or diffusing a situation until a safer opportunity to address it.
- ROLL: Roll refers to seeking out support in debriefing the situation and/or also how to respond to the situation or if it’s best to respond to the situation.
  - The resources available at your institution for students to access during XXX for support include:

**Cases for Small Group:**

The following cases will be covered with your small group. The framework for going through each case is:

1. **Underline the examples of microaggression and discrimination**
   1. Students will be given time to complete this by themselves
2. **How do the microaggressions and discriminations in the case affect team dynamics and patient care?**
   1. Groups will then go over the case together and talk about the different things students underlined. For each case, there will be an accompanying set of “answers” which are meant to be guides for potential things to discuss and by no means an exhaustive list.
   2. Throughout discussion also consider the additional questions as a way to further the discussion
   3. As the group is going through the different microaggressions and forms of discrimination they found in the case, it is important to have them consider how each instance could/does affect patient care.
3. **Apply/practice the frameworks for each case**
   1. **Students will role play by assuming the roles given on the notecards and as they do will consider the following questions:**
      1. What actions/words could you say in this scenario?
      2. What is the intended outcome of that?
      3. What are some limitations to doing that?
   2. Students will break up into smaller groups to do this according to the number of people to roleplay per case
   3. Have students report back to larger group what they came up with for each individual role.
4. **After both cases have been done, provide a debrief space for students to reflect about the workshop**
5. **Before the session ends, please ensure students have filled out the survey.**

**Case #1 10:55 -11:25 am**

**10:55-11:00:Students read case and underline microaggressions and instances of discrimination**

A 82 year-old male, Mr. RS, is admitted from the ED to general medical services with change in mental status. KP is an Asian-American 4th year on her medicine sub-I rotation and picks up Mr. RS on call. On morning rounds, she presents that his change in mental status was likely due to delirium. Upon entering the room, the team finds a somnolent Mr. RS dozing off while having his breakfast. He becomes more alert and looks around the room at the team: a male attending, a male senior resident, a male intern, and two female medical students (KP and a second-year student ST).

The attending asks Mr. RS how he is doing, but his response is difficult to apprehend. When asked to repeat himself, he points to ST, a female Black student, and states, loudly “I do not want her here!” The attending then asks ST to check on the next patient, and she leaves the room.

Changing the subject, the resident asks the patient if the team can examine him. “Of course! Where’s my favorite nurse?” he states with a smile, motioning to KP. While assessing Mr. RS’s pupillary reaction, the attending asks him to focus on a spot across the room. He makes eye contact with KP and asserts slyly,“I can focus on her all day! Where are you from anyway?”

The team concludes the visit and leaves the room, where they reconnect with ST, who has just visited another patient. While discussing the medical plan for Mr. RS, the attending states to the team he feels they “handled the interaction professionally.”

**Examples underlined and points of discussion:**

**Guide discussion based on the issues raised by students: plan to cover at least three of these points spending 5 mins each for a total of 15 mins from 11:00 am - 11:15 am**

1. **“I do not want her here!”**
   1. This is more akin to blatant discrimination or a microassault. Of course, the patient does not explicitly state that he does not want the student in the room *because* of her race. Students may question how we know that this was an incident of discrimination. This incident is based on something that happened to an HMS student, who questioned whether she was asked to leave not because of her race, but rather because the patient simply did not want a student in the room. In this case, the patient does not ask the other female student, who is Asian, to leave the room, increasing our suspicion that his request was discriminatory and based on race. We would like to stress that it is also not intent of the request, but how the intent is *perceived*by the recipient.
   2. This incident provides an opportunity to discuss the different perceptions between the Asian-American and Black students. The main difference between these two students that we would like to explore is their race, as the patient was unlikely to know that the Black student was more junior to the Asian student. The juxtaposition of the patient’s reaction to the two students provides an opportunity to discuss the role of Asian Americans as the “model minority” and how this ideology is used to discredit and alienate Black and Latinx Americans. How can students who benefit from this perception serve as allies to those subjected to more negative stereotypes?
2. **The attending then asks ST to check on the next patient, and she leaves the room.**
   1. This action should be addressed as a separate issue from the microaggression itself. The attending, as the leader of the team, is the most authorized person to respond to the patient’s request. The attending may have thought that refusing the patient’s request in the moment would have further escalated the situation and put ST in greater harm. By honoring the request without explaining to the team why, the attending simply validates the patient’s racism and sends the message to the team that this bigoted behavior is acceptable. The attending leaves the students to question not only why the patient made these comments, but also to hold the attending responsible for complying with the request in the first place.
   2. In terms of responding to the patient’s request, the attending has a few options. One option could be to ask ST to leave the room and facilitating a restorative conversation with the team afterward, as described above. A well-trained attending might employ a framework such as the one created by Kimani Paul-Emile et al. in “Dealing with Racist Patients.” According to this model, the attending would ask the patient to explain why he was requesting that ST leave the room. The patient may realize that his request cannot be adequately justified and concede. Or, the patient may proceed to justify his racist request, in which case the attending would need to respond. The attending could state that there is simply no tolerance of racism, sexism, or any other form of bigotry in the hospital. Regardless of how the attending chooses to respond in the room, it is still the attending’s responsibility to facilitate a conversation with each member of the team following the incident and to ensure that ST and KP are connected to supportive resources afterward.
   3. We intentionally do not focus on analyzing the attending’s response to the patient in this workshop because it is designed for medical students. However, it is important to note that the attending’s response to this patient is inappropriate and ultimately puts an undue burden on the students to navigate this difficult situation on their own.
3. **“Where’s my favorite nurse?”**
   1. The patient mistakes KP, an M4, for a nurse. Many women physicians have stories of being mistaken for nurses based on their gender, and in some cases, based on race and ethnicity as well. Though not directly addressed in this case, physicians and physicians-in-training who are not of the same background as traditional physicians (namely white and male) may be mistaken for other (usually “lower” on the hierarchical rank) members of the healthcare team.
   2. We should also interrogate why it is that we consider being confused for a nurse as problematic. On one hand, gender stereotypes are harmful and frustrating to navigate, especially when we feel that women’s rights have progressed extensively over the last few decades. On the other hand, this frustration may stem from internalized superiority and elitism. Women physicians and physicians-in-training may see themselves as being “better” than nurses and do not want to be confused for one. In fact, some female physicians wear white coats primarily to avoid this confusion.
   3. This statement should be considered in terms of race, as well as gender. The student physician in this case is Asian-American. Asians make up 3% of the U.S. population and about 12% of the physician population. We can imagine a situation in which members of racial groups that are underrepresented in medicine may have an even greater burden of having to justify their role on the healthcare team. For example, 12% of the American population is Black, yet only 4% of all physicians are Black.
   4. It can also be expanded to consider other members of the healthcare team, including patient care assistants and house staff, who are often people of color. These racial and gendered stereotypes may lead to distrust between the clinician and patients and the clinician and other members of the healthcare team. Jennifer Okwerekwu, a Black psychiatry resident at CHA, writes “All this time I spend explaining who I am is time I’m not spending being who I am” when describing the emotional exhaustion of having to continuously explain that she is a doctor.
   5. Assumptions of the roles in the healthcare setting based on anything other than fact can directly harm patient care. This racist and gendered skepticism can affect the ability of a healthcare provider to do their job. Dr. Tamika Cross made national news when a Delta flight attendant prevented her from assisting a patient during an emergency, prompting other physicians of color to share similar stories via the #WhatDoctorsLookLike campaign on social media. Similar phenomenons occur within the healthcare setting. Some physicians of color, especially women, describe being questioned more by nurses than their male counterparts, confirming feelings of self-doubt and inadequacy that contribute to burnout and compromise patient care.
4. **“Where are you from anyway?”**
   1. This microaggression is considered a micro*invalidation*because it demonstrates the assumption that non-white Americans are foreigners. Asian-Americans are often the targets of this “alien in your own land” perception. Other comments that illustrate this perception include “You speak good English” and “Where were you born?” These comments can be interpreted as benign small talk or even complementary; in fact, they are rarely meant to be malicious statements. Recall that the definition of microaggression that we present in the didactic session includes both intentional and unintentional slights. These statements can make the recipient feel that they do not belong and can contribute to the larger political xenophobic climate. It is interesting that the patient asked the Asian-American student where she is from but did not ask the same question to the other members of the team.
   2. The impact of this statement on patient care can take many forms. In some ways, it can be extremely exhausting to have to justify that you belong. It may feel begin to feel like the student is a foreigner not only in the country, but also in the healthcare setting or as a member of the care team. Students who have been asked this question many times may have already developed coping mechanisms, including ignoring these statements, responding with sly remarks, etc.
5. **“I can focus on her all day”**
   1. This comment has a sexual undertone and could be characterized as both a microaggression and blatant harassment on the basis of gender and race. Again, this is an example of a comment that was likely not made with the intent to harm; the patient likely thought he was being complementary and humorous. This sexist comment is directly harmful to women in the healthcare setting, who seek to be treated as equals to their male counterparts. Worse, women are blamed for attracting the male gaze, resulting in the policing of the female appearance with formal policies such as dress code and more informal codes of conduct.
   2. Harassment on the basis of gender especially has no place in the healthcare setting. It is worthwhile to note that this comment was adapted from a comment a preceptor made to a female member of this class of HMS students. The male preceptor asked a patient to focus on the “pretty medical student” and then corrected himself stating, “Oh I’m sorry, nowadays we’re supposed to say ‘strong, independent female.’” In another case, a female HMS student was subjected to harassment from a patient during a geriatrics visit in May. Her preceptor was observing the interview and neither intervened nor approached the student following the interview. Of note, some students have expressed that these incidences are bound to occur, and that it’s “just what comes with being a woman.” Our goal is to challenge this idea and to equip students with the tools to create a learning environment in which these incidences are rare and taken seriously.
   3. In this case, the patient is the one making this comment. Nonetheless, the implications of the male gaze from of other members of the healthcare team are worth noting. Specifically, what should students do if harrassed by another member of the team, especially if that member is of a higher rank?  Stories of female students being sexually harassed by male residents and attendings at HMS and beyond are not uncommon. These incidences are more likely to occur later in medical training when students have more clinical experience. Some of the targets of harassment express fear of retaliation when explaining why they did not report this incident. The national conversation surrounding the #metoo movement has begun to infiltrate the healthcare field. Alleged sexual abuse from Dr. Larry Nassar (Michigan State University and USA gymnastics team doctor), Dr. George Tyndall (USC student health center doctor), and Dr. Richard Strauss (Ohio State University wrestling team doctor) has made national news over the last year. In all of these cases, patients have come forward to describe incidences in which physicians have abused their power.
   4. This statement should be discussed in the context of the student’s Asian-American race. Coupled with the next statement (“Where are you from anyway?”), this is a clear case of exoticization. Asian women in particular are subject to fetishization, stemming from stereotypes of sexual subservience. (While Black women may also be fetishized, the patient’s reaction to the Black student fits more in line with the notion that Black women are undesirable).
6. **“Handled the interaction professionally”**
   1. The preceptor missed a crucial opportunity to address what had just occurred in a meaningful way that could have minimized the harm endured by his team. He not only missed this opportunity, but he also caused additional harm by imposing his expectations of professionalism on his team. “Professionalism” is often rooted in a white supremacist ideology of what behavior looks like, and what behavior is acceptable in professional spaces. These whitewashed codes of conduct leave little room for inclusion of people from different backgrounds and cultures. For instance, a gay student in our class has been told by preceptors that he is “unprofessional” due to his more feminine-presenting demeanor.
   2. It is telling that “professionalism” requires that Black and Latinx people to remain complacent in the face of blatant discrimination. ST was expected to leave the room amicably. It is crucial to recognize that professional behavior is of greater burden to people of color, especially in these situations.
   3. Not all faculty may be equipped with the tools to support learners through an experience like this. Whose role is it to help faculty develop this skill set? Hospital leadership? Learners?

**Additional questions to consider while going through the case to ask students:**

1. **How does the patient’s mental status impact your understanding of this case?Do altered mental states cause patients to express problematic views? Are these the views they truly hold deep down or are they completely foreign? Is this distinction important?**
   1. The patient’s mental status in this case is pertinent, and should be discussed explicitly if not brought up by students. Some students might use the patient’s mental status to justify his comments. Students may think and express that the patient was not in complete control of his own thoughts and actions due to his mental status. The case mentions that the patient is likely delirious and was admitted for a change in mental status. It is possible that the patient’s behavior was exacerbated by, but not *caused by*his change in mental status.
   2. We can debate whether these statements and behavior would or would not have occurred if the patient was in his normal state of mind. However, regardless of whether his mental status should be used as justification, the patient’s comments require a response because *these comments cause harm regardless of intent*(this is a point that should be stressed for all microaggressions).
2. **How do you think age of the patient played a role in the case? How does age in general play a role in what we tolerate?**
   1. This is an elderly patient. There is an assumption that older generations should not be held to the same standards of conduct, perhaps because they grew up in a very different social climate. For instance, this patient may very well not be used to seeing Asian-Americans and/or women in the role of a physician-in-training. This point may be worthy of discussion considering many of the patients students will be caring for are older.

**Role Play:(11:15-11:25)**

Students will break off into groups of 4 and begin from the end of the case when the attending says “handled the interaction perfectly”. Each student will pick a card which be their assigned role.

Roles:

-      KP, Asian American 4th year medical student, female

-      ST, Black 2nd year medical student, female

-      Bystander, 2nd year medical student

-      Attending (may be omitted if don’t have enough students)

-      The student playing this role will serve as the listener and recipient of what ST, KP, and bystander say. They can offer feedback/pushback but serve mainly as a way for students to practice key phrases according to the framework

-      The student who plays this role here should notplay this role again in the next case

Example of responses:

1. KP
   1. During the encounter: **“Mr. RS, we had such a nice conversation this morning - I’m so surprised you’re behaving this way”**
      1. Intended outcome:KP established a rapport with the patient when he was admitted, and he clearly views her favorably. She may be able to harness that connection to correct Mr. RS’s behavior. Note that the point is not to convince Mr. RS to change his racist behavior, as the harm has already been done. Rather, the goal is to *show support for her classmate*and diffuse a tense situation.
      2. Limitations: This is a pretty high-level response and may only be possible for a student who has had more clinical experience and feels comfortable making this somewhat provocative statement in front of the group. Also, as stated above, the harm has already been done to ST.
2. ST point of view
   1. To the attending: **“I feel like I was asked to leave the room because of my race. Can you help me understand this?”**
      1. Intended outcome: To debrief the interaction with the patient and bring it to the attention of the attending. The attending may actually be totally clueless and not realize the impact that Mr. RS had on his student. This also gives ST a chance to hear her attending’s perspective on the issue and to understand why it is that he did not address the statement in the room with the patient and/or outside the room with the rest of the team.
      2. Limitations: Confronting the attending, whose lack of action has contributed to the harm to this student, requires a substantial emotional burden. ST faces the possibility of the attending devaluing her feelings about what transpired either intentionally or simply due to the fact that he can not personally relate to her experience. The attending also has an evaluative role,and speaking to him may be seen as questioning his authority. This speaks to the additional stress that students in this situation, who want to succeed just as their other classmates do, may endure. One of the known limitations in addressing microaggressions and discrimination in the clinical setting is the substantial fear of retribution. Unfortunately, the students most vulnerable in this situation are the ones who face the greatest chance of retribution by being forced to respond as the targets of the microaggression and discrimination. ST must now weigh the emotional stress of ignoring the encounter and her attending’s compliance with the stress of discussing it with him and potentially facing the interpersonal consequences with her superior.
   2. To KP: **“Why do you think he asked me to leave? How did you interpret this situation?”**
      1. Intended outcome:To check in with a student and potential ally about the situation and to get her understanding of what happened. Similarly to discussing with the attending, making this statement to KP gives ST a chance to hear a different perspective on what occurred and understand why KP did not intervene.
      2. Limitations:ST is being very vulnerable in this situation by asking KP about this. She may not feel comfortable with KP or may not know if she can count on KP to be an ally. KP may not believe that anything was wrong with the patient’s request, leaving ST to wonder if she is the one who is overreacting or assuming the worst intentions in the patient. Furthermore, it is possible that there may be a competitive relationship between these two students. KP may believe that the patient’s favorable behavior toward her reflects her clinical competence and may not want to criticize him. Lastly, ST asking this is putting added work on the person who has received the micro-aggression, thus it is important to understand that the emphasis should not be on ST trying to identify allies but for the allies instead to step up and check-in with ST.

1. Bystander -> Upstander: Call it out and name it in a safe space after the event.
   1. To the attending: **“I felt uncomfortable earlier with how our patient treated my colleagues. Could we talk more about it?”**
      1. Intended outcome: Like ST and KP, the bystander has the ability to bring the incident to the attention of the attending. However, unlike ST and KP, the bystander was not directly affected by the patient’s actions. Facilitators should stress to students that it is not only the responsibility of those directly targeted by microaggressions and discrimination to respond. Rather, it is a collective responsibility to ensure a safe learning and working environment for all. This is an especially important role for several reasons. Because the bystander was not directly targeted, they are in a more favorable position within the window of affective tolerance. The bystander is less likely to be in an emotional state that would make reaching out to the attending more difficult. Also, many of the students who are participating in this workshop will likely not be targets of microaggressions, but may be bystanders. Therefore, it is important that students can practice talking points for the bystander role.
      2. Limitations: The fear of retribution is relevant here as well. The bystander may worry that the attending does not agree that the patient’s actions were inappropriate. By bringing this issue to the attending, the bystander might fear that they are challenging the attending’s authority. An additional point to keep in mind and remind students is that when the bystander brings up their concerns, it should come from a place where they are responding to the collective experience rather than speaking on behalf of ST or KP. If the bystander wishes to address specific things ST or KP experience/have concerns with, it is very important for the bystander to check in with ST/KP beforehand and to respect their desires to bring issues up or not.
   2. To ST and KP: **“Our patient’s behavior earlier was completely inappropriate. I can’t imagine how you must be feeling right now. Would you like to talk more about what happened with me?”**
      1. Intended outcome: To provide allyship, letting ST and KP know that the bystander noticed the patient’s inappropriate behavior. This also allows the bystander to empathically connect with ST and KP and provide a supportive listening ear if they would like to discuss what happened with the bystander.
      2. Limitations: While the bystander can provide a unique perspective as someone who was in the room during the incident and as a peer colleague, ST and KP would most benefit from institutional support and a formal review of the events that transpired. Furthermore, as a fellow student, the bystander likely is not qualified to fully debrief the incident. The bystander can and should offer their support to ST and KP, but ultimately, ST and KP should be encouraged to reach out to institutional resources to escalate the issue.

**Case #2 (11:25-11:55)**

**11:25-11:30Students read the case and underline the microaggressions and acts of discrimination**

PS, a Latinx 2nd year medical student, is on his first day of surgical rounds. The team--the attending, a resident, and two medical students (PS and AB, a white female 2nd year)--is moving through rooms quickly in order to get to the operating room. Their next patient is GG, a Spanish speaking woman status post hernia repair, whose 15 year old son is in the room with her. Neither the attending nor residents speak Spanish. Although they have called and are waiting for the interpreter, they are impatient to continue rounds. Finally the attending physician says to the boy, “Son, could you just ask your mom how she’s doing? How the surgical site is healing? Is she passing gas?”

Both mother and son look concerned. PS, who speaks Spanish steps in and says “no se preocupe - don’t worry” and reassures the patient that they are getting the care they need to be ok. The attending says, “PS, could you translate for us?”PS grew up speaking Spanish at home but doesn’t know a lot of medical terminology. Still, he says “yes” in order to be helpful.At that moment, the interpreter enters, and the visit continues.

Later that day, the resident asks PS if he will translate the discharge documents into Spanish for GG before the next morning--”The translating service takes a long timeand we want to get her discharged first thing.I know you’ll do a good job.”

PS spends over an hour that evening meticulously translating the documents that night, which cuts into his study time, but PS is eager to be helpful to the team and the family. He presents the documents to the resident the next morning. The resident doesn’t look up from the computer screen, saying, “Great! Just give them to the nurse.” During team rounds, the attending asks PS some pointed questions about management of post-surgical complications, one of which PS doesn’t know. “You need to spend time learning about your patients in order to take care of them,” the attending says to the team, which PS finds frustrating but acknowledges.

As the team is moving between rooms, the resident turns to the AB and says,“Wow, PS always seems unhappy. He’s not much of a team player, is he?”

**Examples underlined and points of discussion:11:30 -11:45- focus on  three of these points that students raise and discuss each for 5 mins for a total of 15 mins**

1. **“Finally the attending physician says to the boy, “Son, could you just ask your mom..”**
   1. This incident illustrates the difference between a microaggression and discrimination being rooted in **intentionality**. For example, this could be an example of a microaggression from the perspective of the attending as he is in his mind trying to deliver the best care, but regardless the family may experience this negatively as they may perceive it as only being done because they are non-english speaking.
   2. Asking the young boy to translate rather than waiting for the translator puts the young boy in a difficult situation where he has to interpret language he may not know or be aware of the ramifications. It also puts the patient in potential risk of misinformation and danger.
2. **“The attending says, “PS, could you translate for us?””**
   1. This incident also illustrates a microaggression.
   2. Asking the medical student to step in and translate is detrimental both for the medical student and for the patient, particularly as PS does not know medical terminology. In having to translate, PS becomes more concerned with making sure both the medical team and the patient are getting information, but spends no time actually engaging with the information and/or learning from it. Ultimately PS is robbed of the opportunity to work on their clinical reasoning as he has to act as both a cultural broker and a medical student.
3. **“Still, he says “yes” in order to be helpful.”**
   1. This example could reflect internalized bias as PS feels ok giving inadequate translation to the family as they perceive it to be better than nothing. This also highlights the hierarchy of power in the hospitals that may force members of marginalized groups to perpetuate and reinforce harmful/discriminatory practices toward other members of those same groups. Increasing representation in the healthcare field will not fix this unless we are willing to question and dismantle these systems in the first place.
4. **“The resident asks PS if he will translate the discharge documents into Spanish for GG before the next morning”**
   1. Same issue of asking someone with a lack of knowledge of medical terms in Spanish to interpret and now translate an important document for the patient’s care is an issue. Additionally, now there is an extra burden of work that is placed on PS to have to spend time working on this assignment in addition to all of their other clinical responsibilities. Exemplifies in many ways the **minority tax/burden**. Minority tax/burden is/are additional duties, expectations and challenges that accompany being an exception within white male-dominated institutional environments. Here, PS has to take on the additional burden of translating documentation which in most cases they have received no training or support to do.
5. **“The resident saying that the translating service takes a long time”**
   1. Here the workflow is taking precedence over the real work of communicating with the patient. This is seen as OK because of the patient is non-english speaking.
6. **“I know you’ll do a good job.”**
   1. This comment could be seen as passive or even reassuring. But on closer look one can consider how this statement puts a greater burden on PS to live up to expectations the resident has which is unconfirmed if it comes from anywhere credible. The resident is making the assumption that PS knows how to translate well simply because they are Latinx and speak spanish.
7. **“PS spends over an hour that evening meticulously translating the documents that night, which cuts into his study time, but PS is eager to be helpful to the team and the family.”**
   1. Here we clearly see how PS’s medical education is hampered due to having to take on additional responsibilities which fall outside the delineated responsibilities of a medical student. This not only affects PS currently, but if it continues to occur will hurt PS in the long run as they will systematically have less time and opportunity than peers to dedicate their time to improving their clinical knowledge and skills.
8. **“The resident doesn’t look up from the computer screen, saying, “Great! Just give them to the nurse”**
   1. Here the resident is making the assumption that the document is translated correctly which puts the patient in danger. Again, the question to think about is whether or not this behavior would be acceptable with a patient who was english speaking.
9. **“You need to spend time learning about your patients in order to take care of them”**
   1. This comment devalues much of the time and work that PS had to spend translating the documentation. It make the assumption that PS is choosing to not study and to not learn about the patient when in reality PS cared about the patient so much that they were willing to take on extra work they were not qualified to do in order to ensure the patient has better care.
10. **“Wow, PS always seems unhappy. He’s not much of a team player, is he?”**
    1. This is an example of a more classic micro-aggression in which an off hand comment is hurtful to PS because it devalues the time he spent doing extra work (minority tax) relating to his identity.

**Additional questions to consider while going through the case to ask students:**

**1)**   **What are the systems-level issues affecting communication with GG? Factors at the level of the individual interaction?**

**2)**   **Is it ever appropriate to use family members to interpret/translate? Students?**

                                          i)          It is generally unacceptable to use family members to interpret/translate. Hospitals may have protocols by which students can get approval to interpret/translate. Teams should be aware of hospital protocols. But if the student is asked to do so, recognition that this is outside of the role of the student and may affect their ability to do their other duties should be recognized.

3)   **When asked to interpret/translate, what were PS’s options?**

                                          i)         PS could have:

(1)  Stated that they were not trained to be an interpreter and did not want to deliver sub-optimal care

(2)  Expressed deep desire to provide best care and that would mean have a licensed interpreter

(3)  Talked about the situation with the site director in order to ensure that the patient did receive the correctly translated/interpreted information. Talking with the site director could also help PS determine how to best navigate that or future situations

**4)**   **Were there other ways in which PS or other team members could have worked to attain optimal communication?**

                                          i)        PS/other team members could have:

(1)  Called for a phone/video interpreter that gets connected in minimal time

(2)  Come back to round on the patient once the interpreter had arrived

(3)  Determined that the patient would need an interpreter from the start and so proactively request an interpreter any time the team knew they would be caring for the patient.

**5)**   **As the other medical student on the rotation, what are your options in this situation? Is there opportunity to demonstrate allyship?**

a)   The other medical student could have demonstrated allyship by:

                                          i)        Checking in with PS about how they felt in the situation and having the extra burden put on them

                                         ii)        Expressed doubt/concern from the start about the decision to have PS translate

                                        iii)        Discussing the situation later with a third party, such as the clerkship director

**6)**   **In this case, PS does a lot of work for the benefit of patients that is outside of the traditional medical student role and that PS is uniquely capable of doing due to their language and cultural background. In an ideal system, would there be ways that PS could be recognized for this work?**

a) First, please emphasize that PS should have the choice to take on an additional role for their patient, whereas in this case, they are coerced into taking on this role. And that regardless if they are given a choice and choose to do it, it still does not mitigate the impact of the minority tax which PS experiences.

b) Recognition could potentially look like:

                                          i)        Creating some kind of class credit/elective credit for actively serving as a translator/interpreter

                                         ii)        Compensating PS for the additional time spent translating/interpreting

**Role Play: 11:45-11:55 am**

Students will break off into groups of 4 and have two options, to either role play after the attending asks PS to interpret OR role play after the resident asks PS to translate the medical documents.

Roles:

-      PS, Latinx 2nd year medical student, male

-      AB, White 2nd year medical student, female

-      Bystander, 2nd year medical student

-      Attending (may omit this if not enough students)

-      Again, an observatory role. A different student should play the attending this time to give everyone the chance to participate

Example of responses:

Scenario of having to interpret:

1. From PS point of view:
   1. To the attending:**“ I am not sure my spanish is good enough to interpret all of the medical terminology”**
      1. Intended outcome:attending realizes problem and sees the need for the interpreter
      2. Limitations:attending may brush aside a comment like that, could then start viewing PS as not know medical terminology, could be angered with PS for making things “harder”
   2. To the attending: **“I am happy to talk to GG to comfort them, but I think we should wait for the interpreter before talking about any clinical terms”**
      1. Intended outcome: PS still feels like they can offer support and help while also allowing for more time for the interpreter to arrive
      2. Limitations:attending may brush aside a comment like that, could then start viewing PS as not know medical terminology, could be angered with PS for making things “harder”

1. From AB point of view
   1. To the attending: “**Interpreting and having to learn seems pretty difficult, maybe we should wait for the interpreter? I know I could never do both”**
      1. Intended outcome: question the situation a bit by being curious and mentioning the difficulty of the task and perhaps the attending will question own motives
      2. Limitations: speaking up to an attending like this can be scary especially since it involves inserting oneself into the situation

Scenario of having to translate:

1. From PS point of view:
   1. To the resident:**“ I am not sure my Spanish is good enough to translate all of the medical terminology”**
      1. Intended outcome:resident realizes the need to have a translator get the right documentation and does not put the extra burden on PS
      2. Limitations:resident may continue to ask PS to translate or may hold a grudge on PS for not being willing to do it
2. From AB point of view:
   1. To the resident: **“We have so much to prep for rounds tomorrow morning, I don’t know how anyone could add anything else like translating to their to-do list”**
      1. Intended outcome: suggestion helps the resident realize how much extra work that is for PS to do
      2. Limitations: resident may not react at all to comment and/or say that medicine is a lot of work in general and that it’s expected to be busy
