# Supplementary material for: Building a Tool Kit for Medical and Dental Students: Addressing Microaggressions and Discrimination on the Wards
Source: MedEdPORTAL. 2020 Apr 3;16:10893. doi: 10.15766/mep_2374-8265.10893 (PMC7187912; doi:10.15766/mep_2374-8265.10893)
Supplement: Supplementary file 1 — PowerPoint Presentation.pptxCases.docxRole Cards.docxFramework Handout.docxFacilitator Guide.docxAbridged Facilitator Guide.docxPreworkshop Survey.docxPostworkshop Survey.docxText Exercise Criteria.docx [file mep-16-10893-s001.zip › F. Abridged Facilitator Guide.docx]

**Building a Toolkit:**

**Addressing Micro-aggressions and Discrimination on the Wards**

**Sample Schedule for 2hr session:**

**30 minutes: Didactic session**

- XXX and XXX will be leading this session in the XXX. It will be similar to the slides that were presented for the faculty development session with some additional elements.
- The session will introduce the topic, give basic definitions, provide framework, and apply the framework to a relatively simple case so that students have a working understanding of what will happen in small group session

**15 minutes: Transition to small groups**

- At this time students will be entering your room for the small group portion
- Please have them grab a stack of papers
  - In each stack there should be:
    - A copy of case 1 and 2
    - Assigned role note card for case 1 and case 2

**10 minutes: Introduction to Small Group Session**

- **Introductions**
  - Introduce yourselves
  - Ask students to wear name tags, go around and state names
- **Give preview of session**
  - “The session will involve analyzing 2 different cases. For each case, you will underline the different forms of micro-aggressions, talk about how each affects patient care and finally role play how you would act/respond based on your assigned role”
- **Present ground rules** (feel free to add your own)
  - 1. Be 100% present.
  - 2. Respect where everyone lives on the window of affective tolerance.
  - 3. Demonstrate strength by being vulnerable.
  - 4. Keep everything confidential, Vegas style.
  - 5. Be conscious of power dynamics.

**30 minutes: Case 1**

- **5 minutes**: **Identifying micro-aggressions and discriminatory actions/statements**
  - Ask a student volunteer to read the case aloud
  - Direct students to underline the micro-aggressions
  - Direct students to fill out their assigned cards, answering how they believe the micro-aggressions that they identified affect clinical care and team dynamics
- **10 minutes: Group share out**
  - Students share out the microa-ggressions they found and how they think that their particular example impacts clinical care and team dynamics
  - *Most important things to cover:*
    - *Personal attacks that ST (being asked to leave) and KP (being called a nurse/objectified) receive*
    - *End comment from the attending stating how professionally the interactions were handled*
- **10 minutes**: **Role play**
  - Instruct students to turn the card over and look at their assigned role
  - Students will divide into their groups of 4 based on the card they picked up at the beginning of the session
  - Each group should have:
    - ST, Black American, M2
    - KP, Asian-American, M4
    - Bystander, M2 student in the room, not directly involved in case
    - Attending (may be omitted if don’t have enough students)
      - The student playing this role will serve as the listener and recipient of what ST, KP, and bystander say. They can offer feedback/pushback but serve mainly as a way for students to practice key phrases according to the framework
      - The student who plays this role here should not play this role again in the next case
  - Students will pick up the role play right when the case ends - immediately following the attending’s comment that they “handled the interaction professionally”
    - Students will discuss what they would say and do according to the framework (stop, roll, talk)
    - Students will discuss the following questions on their cards:
      - What is the intended outcome of saying/doing that?
      - What are some limitations to saying/doing that?
- **5 minutes**: **Wrap up - group share out**
  - Have a couple of students share out what some responses they came up with were for the roles of KP, ST, and bystander
  - Discuss responses to the questions regarding intended outcome and limitations

**30 minutes: Case 2**

- **You will follow the same format as you did for the first case. However, this case is more complex and students can role play different aspects**
- **5 minutes**: **Identifying micro-aggressions and discriminatory actions/statements**
  - Ask a student volunteer to read the case aloud
  - Direct students to reread the case and underline the micro-aggressions
  - Direct students to fill out their assigned cards, answering how they believe the micro-aggressions that they identified affect clinical care and team dynamics
- **10 minutes: Group share out**
  - Students share out the micro-aggressions they found and how they think that their particular example impacts clinical care and team dynamics
  - *Most important things to cover:*
    - *Minority tax experienced by PS*
    - *The resident’s comment about PS not being a team player and unhappy*
- **10 minutes**: **Role play**
  - Instruct students to turn the card over and look at their assigned role
  - Students will divide into their groups of 4 based on the card they picked up at the beginning of the session
  - Each group should have:
    - PS, Latinx, M2
    - AB, White American, M2
    - Bystander, M2 student in the room, not directly involved in case
    - Attending (may omit this if not enough students)
      - Again, an observatory role. A different student should play the attending this time to give everyone the chance to participate
  - Students will role play how they would respond either to PS being asked to interpret in the patient encounter **OR** PS being asked to translate the document
  - Students will discuss what they would say and do according to the framework (stop, roll, talk)
  - Students will discuss the following questions on their cards:
    - What is the intended outcome of saying/doing that?
    - What are some limitations to saying/doing that?
- **5 minutes**: **Wrap up - group shareout**
  - Have a couple students share out what some responses they came up with were for the roles of PS, AB and bystander
  - Share responses to questions regarding intended outcomes and limitations

**5 minutes: Closing & post-workshop survey**

- Prompt students to fill out the survey they received in their emails
- Provide the opportunity for space for the students to debrief the workshop and indicate that the society lunches are equally an opportunity to continue the conversation of what was learned, talked about, triggering for anyone, etc.
