# Supplementary material for: Building a Tool Kit for Medical and Dental Students: Addressing Microaggressions and Discrimination on the Wards
Source: MedEdPORTAL. 2020 Apr 3;16:10893. doi: 10.15766/mep_2374-8265.10893 (PMC7187912; doi:10.15766/mep_2374-8265.10893)
Supplement: Supplementary file 1 — PowerPoint Presentation.pptxCases.docxRole Cards.docxFramework Handout.docxFacilitator Guide.docxAbridged Facilitator Guide.docxPreworkshop Survey.docxPostworkshop Survey.docxText Exercise Criteria.docx [file mep-16-10893-s001.zip › G. Preworkshop Survey.docx]

Pre-workshop survey

**Building a Toolkit for Medical and Dental Students: Addressing Micro-Aggressions and Discrimination on the Wards**

Thank you very much for planning to attend our workshop! We look forward to working with you. Please complete this survey in preparation for the workshop. This survey will help us understand your needs and experiences and will help us improve the workshop quality for future students. 

This is an **anonymous** survey: we will not report any individual identifying information. The survey should take about 2-3 minutes to complete. We ask that you fill out the survey as completely as possible so we can understand the effectiveness of this workshop and where we need to improve or change it. Thank you for your input! If you have any questions or concerns, please contact XXX

Q1

**IMPORTANT!** In order to keep the survey anonymous, we will be creating a unique study number so we can link your pre and post surveys. Please fill in the following in the space below: (1) Write in the **first letter of your birth month** and (2) the **last 4 digits of your cell phone number.** For example, if you were born in February and the last 4 digits of your number are 9721, your ID number is F9721. 


After we have linked the pre and post surveys, we will replace this ID number with a different study ID number, so there will be no remaining links to your original ID.

________________________________________________________________

Q2
**This section asks about your current knowledge and experience related to the topics of the workshop.**
   Are you familiar with the term "micro-aggression"?

- Yes (1)
- No (2)

If answered YES to Q2 please fill out Q3 and Q4

Q3 Please write 1-2 sentences to explain how you would describe a "micro-aggression."

________________________________________________________________

________________________________________________________________

________________________________________________________________

________________________________________________________________

________________________________________________________________

Q4 In 1-2 sentences, please describe the impact of **micro-aggressions** in the clinical environment on patient care.

________________________________________________________________

________________________________________________________________

________________________________________________________________

________________________________________________________________

________________________________________________________________

Q5 Discrimination is unfair or unequal treatment of an individual based on the group, class, or category to which that individual is perceived to belong. In 1-2 sentences, please describe the impact of **discrimination** in the clinical environment on patient care.

________________________________________________________________

________________________________________________________________

________________________________________________________________

________________________________________________________________

________________________________________________________________

Q6 A micro-aggression is defined as: brief and commonplace daily verbal, behavioral, or environmental indignities, whether intentional or unintentional, that communicate hostile, derogatory, or negative slights and insults toward marginalized groups of people. 


Thinking about the time you have spent in inpatient or outpatient settings at HMS, have you experienced or witnessed a micro-agression directed towards:

|  | No, never (1) | Yes, once (2) | Yes, more than once (3) |
| --- | --- | --- | --- |
| Yourself? (1) |  |  |  |
| Another student or member of the health care team? (2) |  |  |  |
| A patient? (3) |  |  |  |

Q7 Overall, how well prepared do you feel to address micro-aggressions in the clinical learning environment?

- Not at all prepared (1)
- Somewhat well prepared (2)
- Moderately prepared (3)
- Very well prepared (4)
- Extremely well prepared (5)

Q8 Whether or not you have experienced or witnessed micro-aggression in the clinical environment, in your opinion, how challenging are each of the following for YOU in addressing **micro-aggressions**?

|  | Not at all challenging (1) | Slightly challenging (2) | Moderately challenging (3) | Very challenging (4) | Extremely challenging (5) |
| --- | --- | --- | --- | --- | --- |
| Fear of retribution (1) |  |  |  |  |  |
| Difficulty recognizing that a micro-aggression has occurred (2) |  |  |  |  |  |
| Lack of familiarity with what to say or do (3) |  |  |  |  |  |
| Lack of visible allies present who will support me if I speak up (4) |  |  |  |  |  |
| Lack of familiarity with support systems at HMS to address this issue (5) |  |  |  |  |  |
| Lack of certainty of its clinical relevance (6) |  |  |  |  |  |
| Other (7) |  |  |  |  |  |

Q9 How challenging are each of the following for YOU in addressing **discrimination** in the clinical learning environment?

|  | Not at all challenging (1) | Slightly challenging (2) | Moderately challenging (3) | Very challenging (4) | Extremely challenging (5) |
| --- | --- | --- | --- | --- | --- |
| Fear of retribution (1) |  |  |  |  |  |
| Difficulty recognizing that discrimination has occurred (2) |  |  |  |  |  |
| Lack of familiarity with what to say or do (3) |  |  |  |  |  |
| Lack of visible allies present who will support me if I speak up (4) |  |  |  |  |  |
| Lack of certainty of its clinical relevance (5) |  |  |  |  |  |
| Other (6) |  |  |  |  |  |

Q10
**The next two questions are about how you describe yourself.** Do you consider yourself to be a member of a group that is traditionally underrepresented in medicine? (E.g. LGBTQ, economically disadvantaged, African-American/Black, Hispanic/Latinx, Native American (American Indians, Alaska Natives, and Native Hawaiians), Pacific Islander, etc)

- Yes (1)
- No (2)

Q11 To which gender do you most identify?

- Male (1)
- Female (2)
- Non-binary (3)
- Other (4) ________________________________________________
- Prefer not to answer (5)

Thank you very much for taking our survey! Please bring your thoughts and questions to the session on XXX
