# Supplementary material for: Building a Tool Kit for Medical and Dental Students: Addressing Microaggressions and Discrimination on the Wards
Source: MedEdPORTAL. 2020 Apr 3;16:10893. doi: 10.15766/mep_2374-8265.10893 (PMC7187912; doi:10.15766/mep_2374-8265.10893)
Supplement: Supplementary file 1 — PowerPoint Presentation.pptxCases.docxRole Cards.docxFramework Handout.docxFacilitator Guide.docxAbridged Facilitator Guide.docxPreworkshop Survey.docxPostworkshop Survey.docxText Exercise Criteria.docx [file mep-16-10893-s001.zip › H. Postworkshop Survey.docx]

Post workshop survey

**Building a Toolkit for Medical and Dental Students: Addressing Micro-Aggressions and Discrimination on the Wards**
Thank you for attending the workshop! Your participation in the survey is greatly appreciated.

**IMPORTANT!** In order to keep the survey anonymous, we will ask you again to create a unique study number so we can link your pre and post surveys. In the box below, please write in the **first letter of your birth month** and the **last 4 digits of you cell phone number**. For example, if you were born in **F**ebruary and the last 4 digits of your number are **9721**, your ID number is **F9721**. 

As soon as we link your pre and post survey, we will delete this number and replace it with a random study ID number. *Please write in the first letter of your birth month and last 4 digits of your cell phone number here:*

________________________________________________________________

Q1 Now that you have completed the workshop, please write 1-2 sentences to explain how you would describe a micro-aggression.

________________________________________________________________

________________________________________________________________

________________________________________________________________

________________________________________________________________

________________________________________________________________

Medical students start medical school with varying levels of training and preparation to respond to "micro-aggressions" and discrimination in the clinical setting. 

For the item below please rate your skills: (a) **before the start** of the workshop and (b) **now** (after the workshop).

Q2 Ability to recognize a micro-aggression.

|  | Poor (1) | Fair (2) | Good (3) | Very Good (4) | Excellent (5) |
| --- | --- | --- | --- | --- | --- |
| a. How would you rate your skills at the start of your workshop? (1) |  |  |  |  |  |
| b. How would you rate them now? (2) |  |  |  |  |  |

Q3 Your overall preparedness to address micro-aggressions in the clinical learning environment

|  | Poor (1) | Fair (2) | Good (3) | Very Good (4) | Excellent (5) |
| --- | --- | --- | --- | --- | --- |
| a. How would you rate your preparedness at the start of your workshop? (1) |  |  |  |  |  |
| b. How would you rate your it now? (2) |  |  |  |  |  |

Q4 Please highlight the micro-aggressions and discriminatory statements in the case below.

The team intern is presenting a patient who came in to the emergency room last night.

“This is a 42-year-old Hispanic woman who presents for back and neck pain. She is Spanish-speaking so we used the interpreter. She comes in with neck and back pain that radiates to her arms, legs, and head. Basically TBD.” “TBD?” the medical student asks. “Yeah,” the resident replies. “Total Body Dolor.” The intern laughs along. The intern continues “She applied an over the counter cream she bought at a corner store, but without any relief.” The resident, looking at the screen: “Looks like she’s getting her frequent flyer miles in – it’s her third visit in three weeks.”

Q5 Please write 1-2 sentences explaining your reasoning for each selection.

________________________________________________________________

________________________________________________________________

________________________________________________________________

________________________________________________________________

________________________________________________________________

Q6 Please describe in 1-2 sentences the impact of these micro-aggressions and or discrimination on the patient's care.

________________________________________________________________

________________________________________________________________

________________________________________________________________

________________________________________________________________

________________________________________________________________

Q7 Reflecting on the content of this workshop, as the medical student caring for this patient, please describe in 1-4 sentences how would you address this incident.

________________________________________________________________

________________________________________________________________

________________________________________________________________

________________________________________________________________

________________________________________________________________

Q8 Thinking about the time you have spent in inpatient or outpatient settings in medical school, have you experienced or witnessed a micro-agression directed towards:

|  | No, never (1) | Yes, once (2) | Yes, more than once (3) |
| --- | --- | --- | --- |
| Yourself? (1) |  |  |  |
| Another student or member of the health care team? (2) |  |  |  |
| A patient? (3) |  |  |  |

If you answered YES (once or more than once), please answer Q9 and Q10

Q9 Please select the person or persons responsible for the micro-aggression. Please select all that apply.

- Attending (1)
- Fellow (2)
- Intern/Resident (3)
- Medical student (4)
- Nurse (5)
- Social worker (6)
- Patient (7)
- Other (8) ________________________________________________

Q10 Please describe briefly the microaggression(s) that you witnessed.

________________________________________________________________

________________________________________________________________

________________________________________________________________

________________________________________________________________

________________________________________________________________

Q11 In your opinion, after completing the workshop today, how challenging do you think each of the following  may be for YOU in addressing **micro-aggressions**?

|  | Not at all challenging (1) | Slightly challenging (2) | Moderately challenging (3) | Very Challenging (4) | Extremely challenging (5) |
| --- | --- | --- | --- | --- | --- |
| Fear of retribution (1) |  |  |  |  |  |
| Difficulty recognizing that a microaggression has occurred (2) |  |  |  |  |  |
| Lack of familiarity with what to say or do (3) |  |  |  |  |  |
| Lack of visible allies present who will support me if I speak up (4) |  |  |  |  |  |
| Lack of familiarity with support systems at HMS to address this issue (5) |  |  |  |  |  |
| Being unsure of its clinical relevance (6) |  |  |  |  |  |
| Other (7) |  |  |  |  |  |

Q12 How challenging do you think each of the following will be for you in addressing **discrimination** in the clinical learning environment?

|  | Not at all challenging (1) | Slightly challenging (2) | Moderately challenging (3) | Very Challenging (4) | Extremely challenging (5) |
| --- | --- | --- | --- | --- | --- |
| Fear of retribution (1) |  |  |  |  |  |
| Difficulty recognizing that discrimination has occurred (2) |  |  |  |  |  |
| Lack of familiarity with what to say or do (3) |  |  |  |  |  |
| Lack of visible allies present who will support me if I speak up (4) |  |  |  |  |  |
| Lack of familiarity with support systems at HMS to address this issue (5) |  |  |  |  |  |
| Being unsure of its clinical relevance (6) |  |  |  |  |  |
| Other (7) |  |  |  |  |  |

Q13 How could this session be improved in the future? Please be as specific as possible.

________________________________________________________________

________________________________________________________________

________________________________________________________________

________________________________________________________________

________________________________________________________________

Q14 Do you have any other thoughts, comments or suggestions?

________________________________________________________________

________________________________________________________________

________________________________________________________________

________________________________________________________________

________________________________________________________________

Thank you for taking our survey! Your input will help us improve our workshop and better understand what is helpful to you and your peers.
