# Supplementary material for: Building a Tool Kit for Medical and Dental Students: Addressing Microaggressions and Discrimination on the Wards
Source: MedEdPORTAL. 2020 Apr 3;16:10893. doi: 10.15766/mep_2374-8265.10893 (PMC7187912; doi:10.15766/mep_2374-8265.10893)
Supplement: Supplementary file 1 — PowerPoint Presentation.pptxCases.docxRole Cards.docxFramework Handout.docxFacilitator Guide.docxAbridged Facilitator Guide.docxPreworkshop Survey.docxPostworkshop Survey.docxText Exercise Criteria.docx [file mep-16-10893-s001.zip › I. Text Exercise Criteria.docx]

**Microaggressions Post-Survey Text Exercise Criteria**

**TEXT:**

The team intern is presenting a patient who came in to the emergency room last night:. This is a 42 year old Hispanic woman who presents for back and neck pain. She is Spanish-speaking so we used the interpreter. She comes in with neck and back pain that radiate to her arms, legs, and head.

"Basically TBD."

"TBD?" the student asks.

"Yeah," the resident replies. "Total Body Dolor." The intern laughs along.

The intern continues: "She applied an over the counter cream she bought at a corner store, but without any relief." The resident, looking at the screen: "Looks like she's getting her frequent flyer miles in--it's her third visit in three weeks."

**Two micro-aggression comments**

· "TBD" comments

· "frequent flyer" comment

**Proposed criteria**

**0:** Blank/No answer

**1:** Only non-microaggression portions of text highlighted

**2:** 1 or both microaggressions highlighted, AND one or more additional comments highlighted

**3:** Both microaggression comments highlighted AND no additional comments highlighted
